# Evidence that nuclear receptors are related to terpene synthases

**DOI:** 10.1530/JME-21-0156

**Published:** 2022-02-03

**Authors:** Douglas R Houston, Jane G Hanna, J Constance Lathe, Stephen G Hillier, Richard Lathe

**Affiliations:** 1 Institute of Quantitative Biology, Biochemistry, and Biotechnology, School of Biological Sciences, University of Edinburgh, Edinburgh, UK; 2 Program in Neuroscience, University of Glasgow, Glasgow, UK; 3 Medical Research Council Centre for Reproductive Health, University of Edinburgh, Edinburgh, UK; 4 Division of Infection Medicine, University of Edinburgh, Edinburgh, UK

**Keywords:** terpene, sterol, retinoid, nuclear receptor

## Abstract

Ligand-activated nuclear receptors (NRs) orchestrate development, growth, and reproduction across all animal lifeforms – the Metazoa – but how NRs evolved remains mysterious. Given the NR ligands including steroids and retinoids are predominantly terpenoids, we asked whether NRs might have evolved from enzymes that catalyze terpene synthesis and metabolism. We provide evidence suggesting that NRs may be related to the terpene synthase (TS) enzyme superfamily. Based on over 10,000 3D structural comparisons, we report that the NR ligand-binding domain and TS enzymes share a conserved core of seven α-helical segments. In addition, the 3D locations of the major ligand-contacting residues are also conserved between the two protein classes. Primary sequence comparisons reveal suggestive similarities specifically between NRs and the subfamily of *cis*-isoprene transferases, notably with dehydrodolichyl pyrophosphate synthase and its obligate partner, NUS1/NOGOB receptor. Pharmacological overlaps between NRs and TS enzymes add weight to the contention that they share a distant evolutionary origin, and the combined data raise the possibility that a ligand-gated receptor may have arisen from an enzyme antecedent. However, our findings do not formally exclude other interpretations such as convergent evolution, and further analysis will be necessary to confirm the inferred relationship between the two protein classes.

## Introduction

Nuclear receptors (NRs) are essential to animal life, but their evolutionary origins are unknown. NRs are present in Metazoan species including sponges, insects, and vertebrates, but not in Archaea, bacteria, fungi, or plants. NRs are also present in the simplest basal Metazoans including Orthonectida (*Intoshia*) and Porifera (*Trichoplax*), as well as in Desmospongiea (*Amphimedon*). Recent genome sequencing confirms the presence of NRs in Rhombozoa (*Dicyema*), as well as in Ctenophora (*Mnemiopsis*, *Pleurobrachia*, and *Beroe*; not presented). By contrast, conventional NRs are absent from the Choanoflagellida that branched off before the Metazoan radiation. The yeast Pip2/Oaf1 transcription factors reportedly share some similarity to NRs, but they are embedded in an entirely different architecture (Phelps*et al.* 2006). Although sequences resembling the DNA-binding domain have been reported in Choanoflagellida, the crucial ligand-binding domain (LBD) is absent (López-Escardó*et al.* 2019), confirming that true NRs are a Metazoan innovation.

Few new protein domains were acquired at the pre-Metazoa to Metazoa transition (780–540 Mya; Erwin*et al.* 2011), and the majority of innovations were instead generated through rearrangement of pre-existing components (López-Escardó*et al.* 2019). A precursor to the characteristic structure of NRs was therefore probably present before the first multicellular animal species emerged.

NRs are ligand-activated transcription factors that generally comprise two functional domains, the N-terminal DNA-binding domain (DBD) and a C-terminal LBD. In type I NRs, the receptor is sequestered in the cytoplasm by chaperones; ligand binding to the LBD results in receptor release, nuclear translocation, DBD binding to response elements in target genes, and modulation of transcription. By contrast, in type II–IV NRs, the receptor is constitutively present in the nucleus; ligand binding leads to release from a repressor complex, coactivator binding, and modulation of transcription (Mangelsdorf*et al.* 1995, Weikum*et al.* 2018). Of note, the LBD could have arisen independently from the DBD because some NRs lack the DBD or contain an unrelated sequence (Zanaria*et al.* 1994, Seol*et al.* 1996, Reitzel*et al.* 2011), but later loss of the DBD is also plausible. The presence of potential DBD-related sequences in Choanoflagellida, but the absence of LBDs (López-Escardó*et al.* 2019), also suggests that the two domains could have independent origins.

In seeking potential antecedents to the NR LBD, we considered that the inferred earliest NRs are most similar to the NR2 group that includes HNF4, COUP-TF, and the retinoid X receptor (RXR) (Bridgham*et al.* 2010, Holzer*et al.* 2017). These NRs heterodimerize with RXR, bind to response elements for the C30 terpenoid 9-*cis*-retinoic acid in target genes, and modulate their transcription (Nakshatri & Chambon 1994). Hence the positioning of RXR at the epicenter of the ‘Big Bang’ of molecular endocrinology (Evans & Mangelsdorf 2014). In addition to retinoic acid, most other natural NR ligands – including sterols, steroids, vitamin D, and bile acids – are also terpenoids (Moore 1990), suggesting that terpenoids may have been the earliest NR ligands.

**Figure 1:**
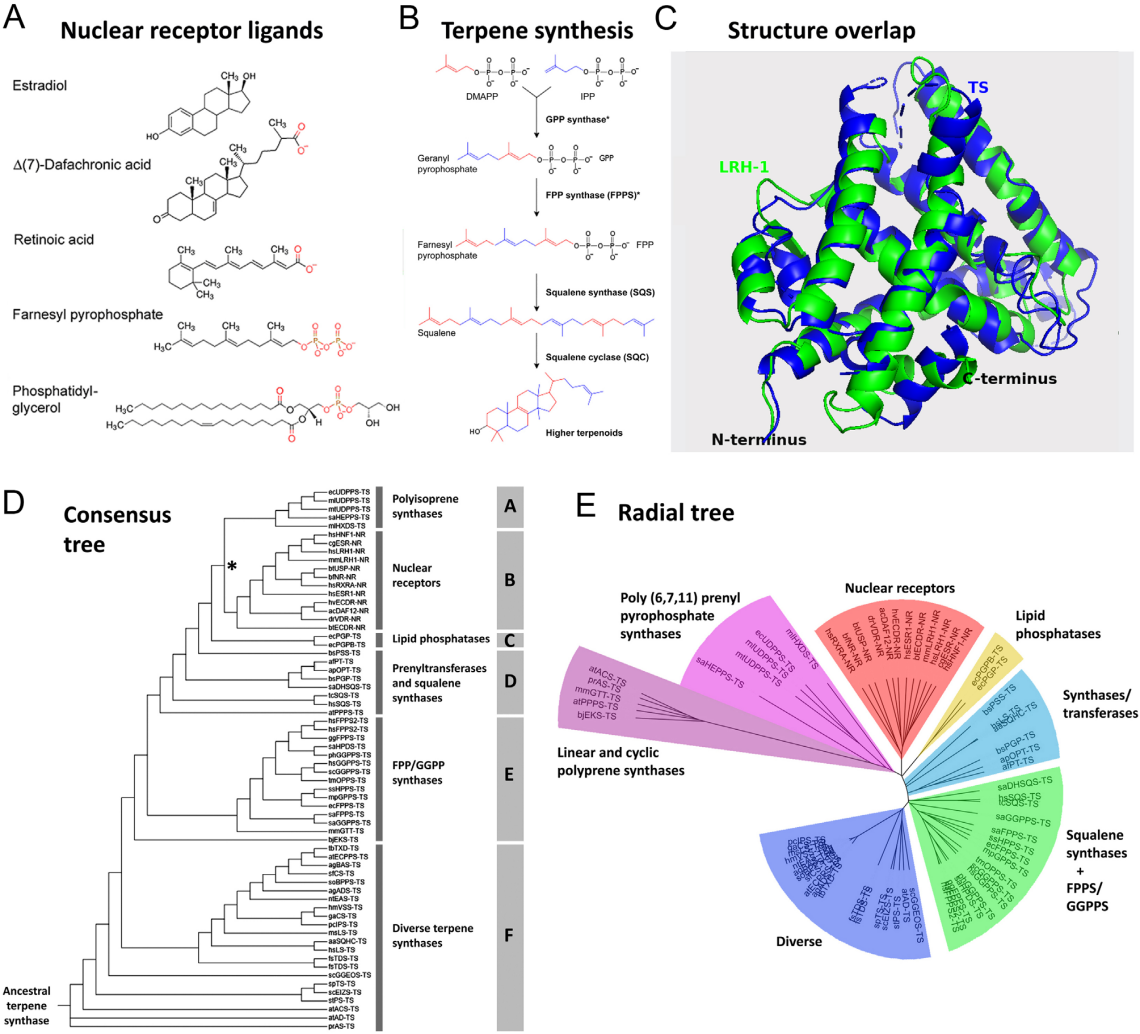
The ligand-binding domains (LBDs) of nuclear receptors (NRs) are similar in 3D structure to terpene synthase (TS) enzymes. (A) Representative ligands for NRs; terminal charged groups are shown in red. (B) The TS pathway; the asterisk indicates that a single enzyme can catalyze the synthesis of both geranyl pyrophosphate (GPP) and farnesyl pyrophosphate (FPP) from dimethylallyl diphosphate (DMAP) and its isomer, isopentenyl pyrophosphate (IPP). (C) Example of 3D overlap of TS monoterpene synthase from the Greek sage plant, *Salvia fruticosa* (PDB: 2J5C) with the LBD of *Mus muscus* NR LRH1 (PDB: 1PK5). The overlap was performed using FATCATflexible (‘Materials and methods’ section), and the combined 3D overlap (*S. fruticosa*monoterpene synthase in blue; mouse LRH1 in green) was imaged using PyMol. The two structures depicted are significantly similar with a raw FATCAT score of 324.37 and a *P* value of 1.05e−04 (structure pairs with a *P* value <0.05 are significantly similar). The two structures have 182 equivalent positions with an RMSD of 3 Å: an RMSD of around 3 Å or lower is generally considered to constitute close homology (‘Materials and methods’ section). The similarity of the sequence alignment computed by FATCAT is 13.20%. Note that protein 3D comparison programs systematically omit ligand; ligand overlap is analyzed in Supplementary Fig. 6. (D) Consensus phylogenetic tree for NRs and TS enzymes established from a 64 × 64 matrix is analyzed using three different 3D structure comparison programs. The NR and TS structures analyzed are listed in Supplementary Table 1. The tree is midpoint-rooted that falls within the TS group; branch lengths are for illustration only. The different polypeptide groups are designated A–F to facilitate comparison with trees drawn using different programs (Supplementary Figs 1, 2, 3, 4, and 5). (E) Radial tree with branch lengths. This midpoint-rooted tree is based on the mean similarity scores obtained with three comparison programs but differs from the consensus tree in (D) because it was computed using a single algorithm (Fitch–Margolaish). Both (D) and (E) locate NRs between polyisoprene synthases and lipid phosphatases.

Terpenoids are a vast superfamily of organic chemicals with unified structures based on the C5 isoprene repeat unit. Their biological properties underpin all of life’s sustainability and communication processes from the formation of the first cell membranes through to steroid signaling and beyond (Summons*et al.* 2006, Nes 2011, Jiang*et al.* 2019). Terpenoid NR ligands including steroids and retinoids (Fig. 1A) are built from C5 isoprene units in a conserved sequence of steps (Fig. 1B) generally involving pyrophosphate as the leaving group (Christianson 2017). These include (i) generation of a pyrophosphate-activated C15 trimer, farnesyl pyrophosphate (FPP); (ii) head-to-head linkage of two FPP to generate C30 squalene; followed by (iii) cyclization to generate basic C30 sterols and related molecules (Hillier & Lathe 2019). The key enzymes involved – FPP synthase (FPPS), squalene synthase (SQS), and squalene cyclase (SQC) – belong to the diverse TS clan of enzymes (https://pfam.xfam.org/clan/Terp_synthase) whose representatives are already present in Archaea and that are conserved between bacteria, yeast, plants, insects, and vertebrates (Ourisson & Nakatani 1994, Jiang*et al.* 2019, Rudolf & Chang 2020).

Given the ubiquity of terpene biosynthesis via conserved TSs that generate NR ligands such as retinoids and steroids (Hillier & Lathe 2019), we postulated that the LBD of NRs might have evolved from a TS enzyme that bound a structurally similar polyisoprene (steroid-like) substrate (or product). We report here suggestive structural and sequence similarities between TSs and the LBDs of NRs that could point to terpenoid forerunners of NR signaling at the Metazoan dawn.

## Materials and methods

### 3D comparisons

A 64 × 64 matrix of representative TS enzymes (*n*  = 52) and NRs (*n*  = 12) was assembled. Because the structure of NR LBDs is relatively well conserved between different NRs, we selected a shortlist of LBDs ranging from nematodes to humans (for which structures are available) to emphasize the diversity of ligand binding. TS enzymes were selected from the Protein Family (PFAM) Database to represent the diversity of the TS clan of enzymes (https://pfam.xfam.org/clan/Terp_synthase). The enzyme and receptor structures and their access codes are presented in Supplementary Table 1 (see section on supplementary materials given at the end of this article). Structures were accessed from the Research Collaboratory for Structural Bioinformatics (RCSB) Protein Databank (PDB; https://www.rcsb.org/). Visualization of protein structures and 3D overlaps employed PyMol (https://pymol.org/2/). Pairwise comparisons (4068 in each case) were performed in triplicate using the downloaded Linux command-line versions of FATCAT2 (flexible structure alignment by chaining aligned fragment pairs (AFPs) allowing twists) flexible version (FATCATflexible) and FATCATrigid (Ye & Godzik 2003), and also Java rigid-body superposition combinatorial extension (jCE) (Shindyalov & Bourne 1998). The FATCAT tool is available online at RCSB PDB (https://www.rcsb.org/alignment) and at the University of California (http://fatcat.godziklab.org). The output consists of (i) a raw score, (iii) a *P* value, and (iii) an RMSD differential between each of the two structures. The raw score calculation is based on the minimum number of transformations required to optimize the alignment between the two structures and the number of equivalent positions found in the alignment. The equivalent positions are those regions of the proteins that can be identified as AFPs, in other words, homologous segments (Ye & Godzik 2003). The RMSD is a measure of the average distance between the Cα atoms of the two structures and is often quoted as a measure of alignment quality (Jewett*et al.* 2003); an RMSD of around 3 Å or lower is generally considered to constitute close homology (Reva*et al.* 1998). However, because the raw score takes into account both the degree and the extent of similarity, multiple comparisons were based on this metric. We refer to the other parameters where appropriate, and the correspondence between the similarity score and the *P* value of each alignment is given in Supplementary Data 1. In all three cases, the results of each comparison were expressed as a similarity score that was typically in the range 0–1000 (96.9% of values; FATCATflexible). The mean score for NR vs NR (FATCATflexible) was 486, and for TS vs TS, it was 431 (some TS/TS values scores fell to under 100; Supplementary Data 1); because outlier (>1000) values were likely to bias the comparison, values >1000 (3.1% of all comparisons) were converted to 1000 before further analysis (Supplementary Data 1). To determine the extent of distortion required to superimpose a terpene synthase (FPPS) onto an NR (ESR1), the combinatorial extension (CE) structural alignment algorithm (Shindyalov & Bourne 1998) was used to align the first three helices from the FPPS core onto the first three helices of the ESR core, and the rotation involved in this movement relative to other helices was measured within PyMol.

### Tree drawing

Similarity scores (0–1000) were first converted to distance values (1.0–0.0) by division by 10^3^, subtraction of 1.0, and rectification. The pairwise values (full spreadsheets are given in Supplementary Table 2) were separately submitted as matrices to PHYLIP (phylogeny inference package) version 3.57c by Joseph Felsenstein (University of Toronto; http://bar.utoronto.ca/webphylip/) under phylogeny methods/distance matrix for analysis by (i) the Fitch–Margoliash method (Fitch & Margoliash 1967); (ii) neighbor joining (Saitou & Nei 1987); (iii) UPGMA (unweighted pair group method with arithmetic mean) (Sokal & Michener 1958). Trees were drawn using http://bar.utoronto.ca/webphylip/ (plot trees/draw cladograms and phenograms) with output style = phenogram; tree grows = vertically; use branch lengths = yes; angle = 90°; ancestral nodes = weighted average. The same trees were generated following randomization of the input sequences. All three comparison methods and all three tree-drawing programs generated comparable results (Supplementary Figs 1, 2, 3, 4, and 5), notably with regard to the TS–NR branchpoint, and a ‘consensus’ tree was assembled that amalgamates all comparisons. Because TS enzymes precede NRs in the evolutionary timeline (TS enzymes are present in Archaea and bacteria, but both taxa lack NRs), the tree is rooted in the weighted average that falls within the TS clan of enzymes. For the radial tree, the mean similarity score was calculated for the three different comparison programs; the tree was computed using Fitch–Margoliash at the University of Toronto, and drawn using Interactive Tree of Life (iTOL) at https://itol.embl.de/itol.cgi.

### Molecular docking

Ligands dafachronic acid (DAFA), estradiol (E2), FPP, and phosphatidylglycerol (PG) were docked into the crystal structures of the following protein receptors, excluding the physiological ligand: human estrogen receptor (ESR1; PDB: 1QKU), human liver receptor homolog-1 (LRH1; PDB: 1YOK), *Caenorhabditis elegans* NR (DAF-12; PDB: 3GYT), and chicken (*Gallus gallus*) farnesyl pyrophosphate synthase (FPPS; PDB: 1FPS), using the program PSOVina2 (Tai*et al.* 2018). Structures were downloaded from PDB, water molecules and other heteroatoms were removed, and the program PDB2PQR 2.1.1 (Dolinsky*et al.* 2007) was used to assign position-optimized hydrogen atoms utilizing the additional PropKa2 algorithm (Li*et al.* 2005) with a pH of 7.4 to predict protonation states. The MGLTools 1.5.6 (Morris*et al.* 2009) utility prepare_receptor4.py was used to assign Gasteiger charges to atoms. Hydrogen atoms were assigned to compound structures using OpenBabel 2.4.1 (O’Boyle*et al.* 2011), utilizing the -p option to predict the protonation states of functional groups at pH 7.4. The MGLTools utility prepare_ligand4.py was used to assign Gasteiger charges and rotatable bonds. PSOVina2 was used to automatically dock the compounds into the crystal structures and calculate a predicted binding pose and free energy. A grid box that encompassed the maximum dimensions of the ligand plus 12 Å in each direction was used; all other parameters were set to default. PyMol (PyMOL Molecular Graphics System, Version 2.0 Schrödinger LLC) was used to visualize the results.

### Ligand overlap in TS vs NR 3D structural alignment

3D structure overlaps combined with conservation of the locations of contact residues between TS enzymes and NR LBDs indicate that the positions of their respective ligands overlap in 3D. However, protein structure comparison programs systematically omit ligands. To formally address ligand overlap, we selected two representative NR structures (*Escherichia coli* OPPS, PDB: 3WJN and *Homo sapiens* ESR, PDB: 1QKU). These were first overlapped using FATCATflexible. The molecular structures of each polypeptide were then separately extracted from the 3D overlap file, and the cognate ligands (FPPS and estradiol, respectively) were separately docked (‘Materials and methods’ section above) into the central cavities of these modified structures. Finally, the docked structures (now with the same molecular coordinates relative to each other) were combined into a single file for visualization using PyMol (Supplementary Fig. 6).

### Primary/secondary sequence alignments

Primary sequences for NR LBDs and TS enzymes were downloaded from the RCSB PDB. The positions of α-helices were manually registered from the PDB structures. For *Amphimedon queenslandica* NR2, no crystal structure is available, and the extents of the α-helices were predicted using three prediction programs: AGADIR (Centro de Regulacióò Genòmica, Barcelona, Spain; http://agadir.crg.es) (Muñoz & Serrano 1997), Jpred4 (University of Dundee, UK; http://www.compbio.dundee.ac.uk/jpred4) (Drozdetskiy*et al.* 2015), and PredictProtein (Technical University of Munich, Germany; https://open.predictprotein.org/) (Yachdav*et al.* 2014). All three programs gave essentially the same result. NR sequences were aligned using Clustal Omega (Sievers*et al.* 2011) at the European Bioinformatics Institute (EBI, Cambridge, UK; https://www.ebi.ac.uk/Tools/msa/clustalo/) and COBALT (constraint-based multiple alignment tool) (Papadopoulos & Agarwala 2007) (NCBI; https://www.ncbi.nlm.nih.gov/tools/cobalt/re_cobalt.cgi). TS sequences were aligned using the same tools, guided by the detailed alignments presented in the NCBI Conserved Domain Database (https://www.ncbi.nlm.nih.gov/Structure/cdd/cdd.shtml) and in the EMBL-EBI Protein Family (PFAM) database (https://pfam.xfam.org/clan/Terp_synthase). For more distant primary sequence relationships, we employed PRRN (progressive pairwise alignment with iterative refinement) (Gotoh 1982, 1996) (Kyoto University; https://www.genome.jp/tools-bin/prrn); all alignments were checked by secondary structure matching and by 3D matching using the sequence display option of FATCATflexible to reach a consensus.

### Primary sequence homologies to the DHDPPS subfamily

We used a large compendium of NR LBD sequences to screen the genomes of species that mark the pre-Metazoa to Metazoa transition, including Chanoflagellida, Ctenophora, Porifera, and Mesozoa (Orthonectida and Rhombozoa). No significant matches were found in Choanoflagellida that diverged before the Metazoan radiation, but sequences were detected as expected for the Porifera *Amphimedon queenslandica* and the Placozoa *Trichoplax aderens*. This analysis confirmed that aqNR1 is most similar to RXR, whereas aqNR2 is most similar to HNF4 (not presented). In addition, matches were found in the Cnidaria *Orbicella faveolata* and in the Orthonectida (Rhopaluridae) *Intoshia linei* (Supplementary Figs 7 and 8). These generated a set of 18 ‘early’ NR LBDs (protein sequences are given in Supplementary Fig. 8) that were used to search (tBLASTn at NCBI) for primary sequence homologies to diverse TS enzymes; this identified the DHDPPS subfamily, including its obligate partner proteins NUS1 and NOGOBR, as being particularly closely related. A selection of representative DHDPPS, NUS1, and NOGOBR protein sequences were retrieved from NCBI and compared against the primary sequences of the 18 early NRs using the program PRRN (default settings) as implemented on the GenomeNet website (Tokyo, Japan; https://www.genome.jp/tools-bin/prrn); a center-rooted tree was generated using the UPGMA algorithm, and drawn using iTOL at https://itol.embl.de/itol.cgi. To refine the tree, alignment and phylogenetic reconstructions were performed using the function ‘build’ of ETE3 v3.1.1 (Huerta-Cepas*et al.* 2016) implemented on the GenomeNet website (https://www.genome.jp/tools-bin/ete), and the ML tree was inferred using RAxML v8.1.20 using the model PROTGAMMAJTT at the same site and default parameters (Stamatakis 2014). Branch supports were computed with 100 bootstrap trees (Supplementary Fig. 7). For a closer inspection of primary sequence alignments, we first used TCoffee (Notredame*et al.* 2000, Garriga*et al.* 2021) to separately align (i) the sequences of the ‘early’ NR LBDs and (ii) the sequences of a selection of NUS1 sequences (NOGOBR was omitted because, although NUS1 and NOGOBR are held to be orthologs, Data presented herein (see Results’ section) reveal that they may fall into two distinct subgroups). The separate alignments are presented in Supplementary Fig. 8 (left). The same approach was then used to jointly align the NR LBDs with NUS1 sequences (Supplementary Fig. 8, right). As shown in the figure, both NRs and NUS1 sequences comprise three conserved regions (numbered 1–3), where red coloration is considered to represent ‘good’ alignment (Supplementary Fig. 8 legend); these same three domains match in the joint NR/NUS1 alignment. The full consensus alignment is provided in Supplementary Fig. 9.

**Figure 3:**
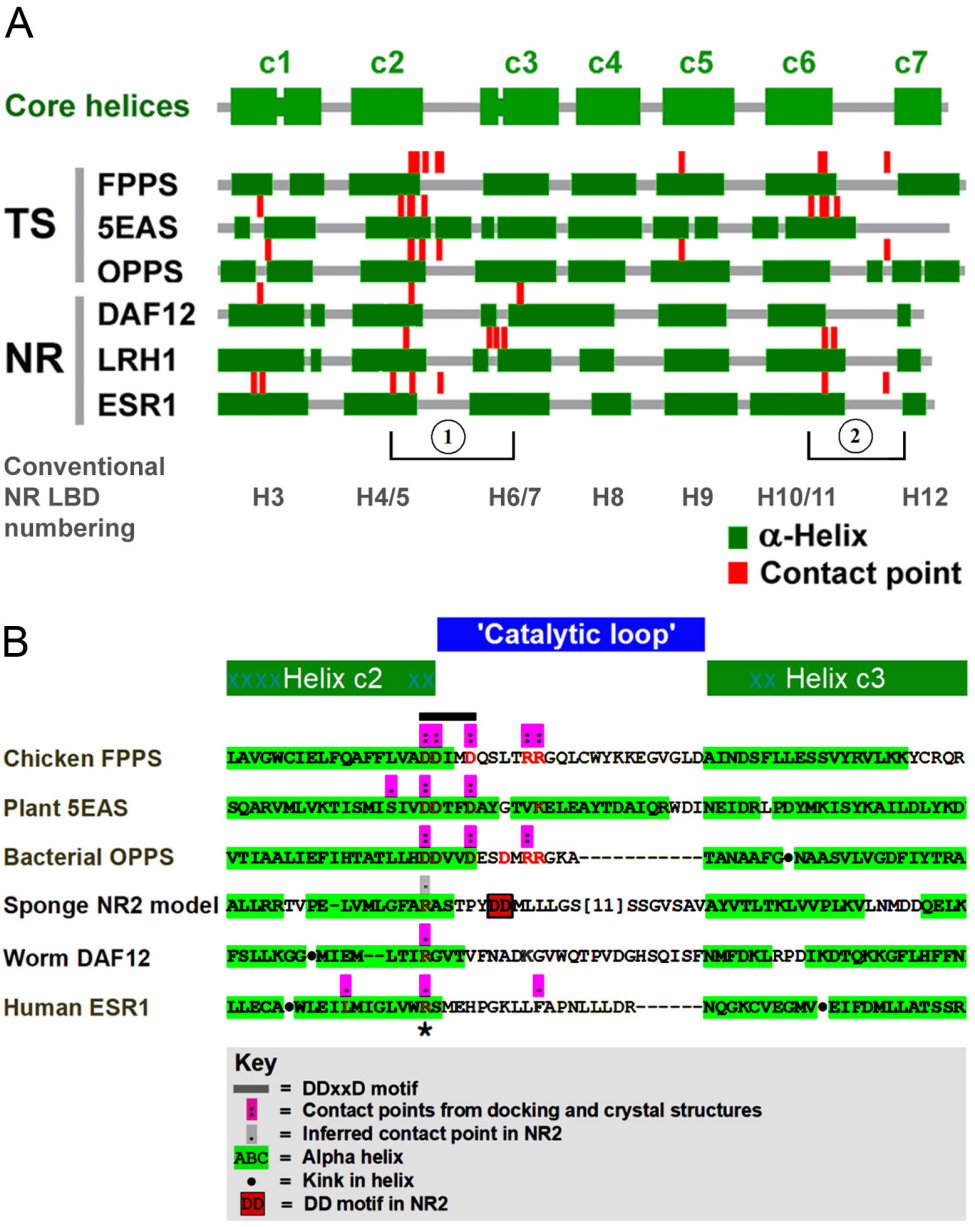
Ligand-binding domains (LBDs) of ‘early’ nuclear receptors (NRs) are most similar to the dehydrodolichyl pyrophosphate synthase (DHDPPS)/NUS1/NOGOB receptor (NOGOBR) subfamily of terpene synthase enzymes. A group of early NRs was compiled from the sponge *Amphimedon queenslandica*(aq), Placozoan *Trichoplax adherens* (ta), stony coral *Orbicella faveolata* (of), and the marine Orthonectid *Intoshia linei*(of). Homology searching revealed that these are similar to the DHDPPS/NUS1/NOGOBR subfamily; panel (A) shows a midpoint-rooted phylogenetic tree constructed using PRRN (Kyoto University Bioinformatics Center, Kyoto, Japan) and the UPGMA algorithm. Branch lengths are to scale, indicating that the early NRs are similar to both NUS1/NOGOBR and their binding partner DHDPPS that are known to be evolutionarily related. Asterisks indicate the positions of aqNR1 and aqNR2. A detailed tree is presented in Supplementary Fig. 7. (B) Primary sequence homologies detected by the T-Coffee multiple sequence alignment (MSI) program M-Coffee (Center for Genomic Regulation, Barcelona, Spain) that combines the results of different alignment programs. The human estrogen receptor hsESR1 has been added for reference; WAKR and WRS represent conserved motifs in ESR1, where WRS is a primary ligand contact site ligand. An extended alignment and further details are given in Supplementary Fig. 8, where amino acid sequence similarities according to functionally exchangeable amino acids in the key regions ranged from 23% to 48%. Prefixes for the aligned sequences are: aq, *Amphimedon queenslandia*; cb, *Caenorhabditis brenneri*; dr,* Danio rerio*; dv, *Drosophila virilis*; hs, *Homo sapiens*; il, *Intoshia linei*; of, *Orbicella faveolata*; sc, *Saccharomyces cerevisiae*; ta, *Trichoplax adherens*; ts, *Trichoplax*sp. H2; xt, *Xenopus tropicalis*.

**Figure 2:**
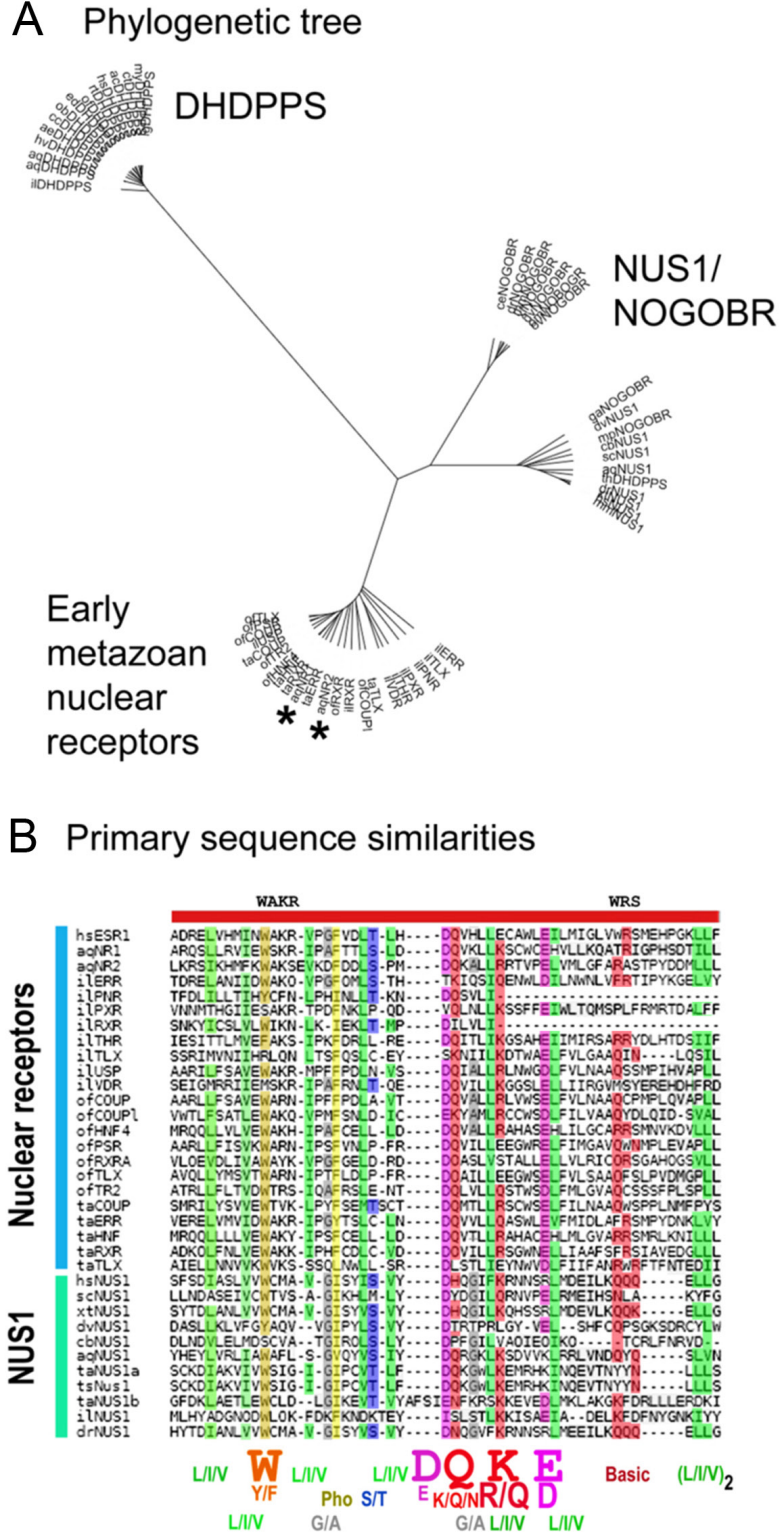
Alignment of terpene synthases (TSs) and nuclear receptors (NRs) showing conservation of α-helices and clustering of ligand contact points in two subregions (1 and 2). (A) Overall alignment of core α-helices c1–c7 between NRs and TSs (the full version of the alignment is given in Supplementary Fig. 9), showing conservation of the α-helical framework and ligand contact points between the two groups. Core helices c1–c7 correspond to NR helices H3, H4/5, H6/H7, H8, H9, H10/11, and H12 as defined by Markov*et al.* (2017), noting that the precise extents of the α-helical segments differ between different crystal structures of the same protein. Full numbering of residues within the crystal structure of each protein is given in Supplementary Fig. 9. (B) Detailed map of the c2–c3 junction (encompassing region 1 in A) highlighting replacement of the DDxxD motif in TS enzymes by a single arginine residue (asterisk) in NRs. Protein structures depicted are DAF12, *Strongyloides stercoralis* (nematode) DAF-12 nuclear receptor (PDB: 3GYT); 5EAS, *Nicotiana tabacum* (tobacco) 5-epi-aristolochene synthase (PDB: 5EAS and 5EAT); ESR1, human estrogen receptor α (PDB: 1QKU, 2OCF, other); FPPS, *Gallus gallus* (chicken) farnesyl pyrophosphate synthase (PDB: 1FPS); NR2, *Amphimedon queenslandica* (sponge) NR2 model (see ‘Materials and methods’ section); LRH1, human liver receptor homolog 1 (PDB: 1YOK); OPPS, *Escherichia coli* octaprenyl pyrophosphate synthase (PDB: 3WJN).

## Results

A molecular precursor to the signature LBD of NRs is likely to have been present before the Metazoa emerged. In seeking a potential antecedent, we considered that protein structure is far more conserved than the primary sequence (Yang & Honig 2000, Illergård*et al.* 2009). We therefore performed 3D structure comparisons between NR LBDs and a range of potential candidate terpenoid-binding enzymes that could have provided the framework for the NR LBD. These included enzymes such as retinoid- and steroid-metabolizing hydroxysteroid dehydrogenases and cytochromes P450. All such groups examined, with the exception of TS enzymes, failed to reveal significant structural similarities; we therefore focused on the clan of TS enzymes.

### 3D structure overlaps suggest that NRs may be related to TS enzymes

To systematically address structural similarities between NRs and TS enzymes, we assembled a 64 × 64 matrix of representative TS enzymes and NR LBDs (‘Materials and methods’ section and Supplementary Table 1) and performed pairwise comparisons using three different 3D structure comparison programs. This revealed that the overall structures of NR LBDs are similar to those of TS enzymes (pairwise *P* values for NR vs TS comparisons were in the range 0.02 to <0.001 for closest matches; Supplementary Data 1). A typical overlap between the 3D structures of a TS and an NR generated by the 3D comparison program FATCATflexible is given in Fig. 1C (ligand overlap is addressed in the next section). The 12,288 comparisons not only emphasized the structural diversity of the TS clan but also revealed that some TS structures are more closely related to NR LBDs (higher similarity score) than to other TS enzymes (distance scores are given in Supplementary Table 2).

To validate our 3D comparison approach, we separately compared the trees generated by structure/structure vs sequence/sequence comparisons for TS enzymes and NRs. The two types of analysis gave very similar and often identical trees (not presented), confirming that 3D structure comparisons are a valuable adjunct to sequence-based comparisons, particularly when distant protein families are being compared.

Phylogenetic tree drawing based on structure placed NRs as a sub-branch of the TS clan most closely related to the polyisoprene synthases and lipid phosphatases (Fig. 1D). The same finding was reiterated in all three 3D comparison methods and with all phylogenetic tree-drawing programs (Supplementary Figs 1, 2, 3, 4, and 5), notably regarding the branch point between TS enzymes and NRs. Figure 1E presents a midpoint-rooted radial tree with branch lengths, also indicating that NRs may be more closely related to polyisoprene synthases and lipid phosphatases. These data therefore argue that NRs could potentially have arisen as a sub-branch of TSs.

### A conserved seven-helix core

Because some TS enzymes contain tandem duplications and can contain over 30 α-helical segments, and some NR LBDs harbor long extensions (e.g., NR2 from *Amphimedon queenslandica*), to identify a conserved core of α-helical segments that characterizes both NRs and TSs, we made pairwise 3D comparisons after stripping away extraneous N- and C-terminal extensions in both TS enzymes and NRs. This analysis revealed that the crucial LBD of both TSs and NRs is constituted by a minimal contiguous (no large insertions or deletions) core of seven α-helices that overlap in their primary through tertiary structures (Fig. 2). Because standard numbering for helices differs between TSs and NRs, we number these helices in both groups core (c) helices c1–c7; correspondence with conventional NR helix numbering is shown in Fig. 2.

This allowed us unambiguously to align the core α-helical segments of representative NRs to their TS counterparts (Fig. 2 and Supplementary Fig. 9) based on the positions of the seven core α-helices, 3D structure overlaps, and primary sequence comparisons. Because residue numbering differs between species, isoforms within a single species, and even between different crystal structures of the same protein, in all cases, we provide the primary sequence of human ESR1 isoform A as a reference point (Fig. 2).

### Ligand-binding site overlap between NR LBDs and TS enzymes

Recognizing that similar structures could have arisen fortuitously, we asked whether the ligand-binding pockets of TS enzymes and NR LBDs overlap in 3D, as reflected by overlaps in the positioning of TS substrates/products vs NR ligands in the overlapped structures. Because protein 3D structure comparison programs systematically omit ligands, we mapped ligand contact sites to give a picture of the overall disposition of ligand within the binding pockets of NR LBDs and TS enzymes.

To map contact sites, we (i) built on known ligand-contacting residues in both NR LBDs and TS enzymes (RCSB PDB; ‘Materials and methods’ section) and also (ii) performed reciprocal docking studies (computer-based docking simulations; ‘Materials and methods’ section) of a key TS enzyme ligand (FPP, both TS ligand and product, and also a known NR ligand) and representative NR ligands (estradiol, PG, and DAFA; Fig. 1A) into key TS enzyme and NR structures. Docking was performed on the complete native structures rather than on the core helices.

In all cases where crystal structures were available containing the corresponding ligand, docking accurately reiterated the crystal ligand pose (Supplementary Table 3). This, combined with further docking studies, revealed that the locations of contact sites in both TS enzymes and NRs are conserved, as mapped to primary, secondary, and tertiary structures of the proteins (Fig. 2, also Supplementary Fig. 9 and Supplementary Table 3), demonstrating that the ligand-binding pockets overlap in 3D. Of note, the primary ligand contact site in TS enzymes (specifically the Asp-rich DDxxD catalytic motif) accurately aligns in 3D with the major contact site in NRs (exemplified by the W**R**S motif in ESR1) in the overlapped structures.

To formally address ligand overlap, we used FATCAT in flexible mode to compare *E. coli*OPPS to *H. sapiens* ESR1. The FATCAT alignment has a *P* value of 9.49e−04 and a raw score of 349.82. The 3D structures of the overlapped polypeptides were extracted and used for molecular docking with their respective ligands (farnesyl *S*-thiol-pyrophosphate, FSPP, and E2, respectively), the docked structures were then combined and visualized with PyMol. As shown in Supplementary Fig. 6, the placements of FSPP and E2 overlap in 3D within the combined structures. Importantly, both ligands are predicted to interact with the major contact sites DDXXD and WRS. Hence, in addition to (i) primary through tertiary structural similarities, the conservation of (ii) contact sites in 3D (and by inference the placement of ligand within the ligand-binding pocket), as well as (iii) direct evidence of ligand overlap, argues that both the overall structures and the ligand-binding sites are conserved between NR LBDs and TS enzymes, adding weight to the suggestion that TS enzymes and NRs may be evolutionarily related.

Interestingly, structural alignments provided evidence for an ancestral internal duplication in TS enzymes. The primary ligand-binding site in TS enzymes (‘site 1’) is defined by the catalytic DDxxD motif (Christianson 2017) at the c2/c3 junction. However, many TS enzymes (e.g. chicken FPPS) contain a second DDxxD motif at the c6/c7 junction (‘site 2’). Site 2 appears to be a relic of an ancestral duplication (Supplementary Data 2) that is present even in Archaeal enzymes. In the 3D protein structures, the two sites are in close proximity. In some TS enzymes, site 2 contributes to catalysis, whereas in others it represents an allosteric site that modulates enzyme activity (Supplementary Data 2). Although the evidence for NRs is much weaker, the same duplication may also be present (Supplementary Data 2). In NR LBDs, site 1 is the principal ligand-binding site, but some large ligands extend beyond site 1 into the inferred site 2, with implications for NR pharmacology (Supplementary Data 2).

In subsequent experiments, we docked the classical bisphosphonate FPPS inhibitor, zoledronic acid (ZA), into ESR1 (and also the estrogen-related receptor ERRG). This revealed that ZA binds into the same ligand-binding cavity that is occupied by estradiol, and probably also binds to the estrogen-related receptor ERRG (Supplementary Data 3), providing further evidence that the mode of ligand binding is conserved between TS enzymes and NR LBDs.

### NRs are most similar to the TS dehydrodolichyl pyrophosphate synthase subfamily

Structural and docking analyses implicate polyisoprene synthases as relatives of NR LBDs. However, structural homologies alone may be misleading and could be generated by convergent evolution of protein structures adapted to binding structurally similar ligands. We therefore sought confirmation-based primary sequence homologies. We argued that NRs at the base of the Metazoan radiation would be most informative. In addition to the two receptors aqNR1 and aqNR2 from the sponge *Amphimedon queenslandica* (Bridgham*et al.* 2010) and four receptors from the free-living Placozoan *Trichoplax adherens* (Baker 2008), we retrieved NR LBD sequences for the stony coral *Orbicella faveolata* (Prada*et al.* 2016) (five NR sequences) and the free-living marine Orthonectid *Intoshia linei* (Mikhailov*et al.* 2016) (seven sequences) (‘Materials and methods’ section).

Sequence comparisons using this collection of 18 ‘early’ NRs suggested that NR LBDs are most similar to the *cis*-isoprene transferases (Fig. 3A, an extended phylogenetic tree is given in Supplementary Fig. 7). These atypical TS enzymes include DHDPPS and its evolutionarily related obligate partner NUS1, also known as NOGOB receptor (NOGOBR) (Harrison*et al.* 2011, see also Grabinska*et al.* 2017, Ma*et al.* 2019, Edani*et al.* 2020); indeed, there are potential primary structure similarities between NUS1 and NR LBDs (Fig. 3B and Supplementary Fig. 8). Of note, this subgroup is also built around a seven-helix core (Bar-El*et al.* 2020), and the regions of similarity overlap accurately with the conserved seven-helix core identified in NRs (compare Supplementary Figs 8 and 9); NR LBDs may therefore be evolutionarily related to this specific TS subfamily. Nevertheless, given that NRs probably had a single, unique, evolutionary origin (‘Discussion’ section), this similarity does not necessarily pinpoint the exact forerunner to the LBD, although a potential relationship to the DHDPPS/NUS1/NOGOBR group would appear to be consistent with these data.

### Structural changes between a TS enzyme and an NR LBD

**Figure 4:**
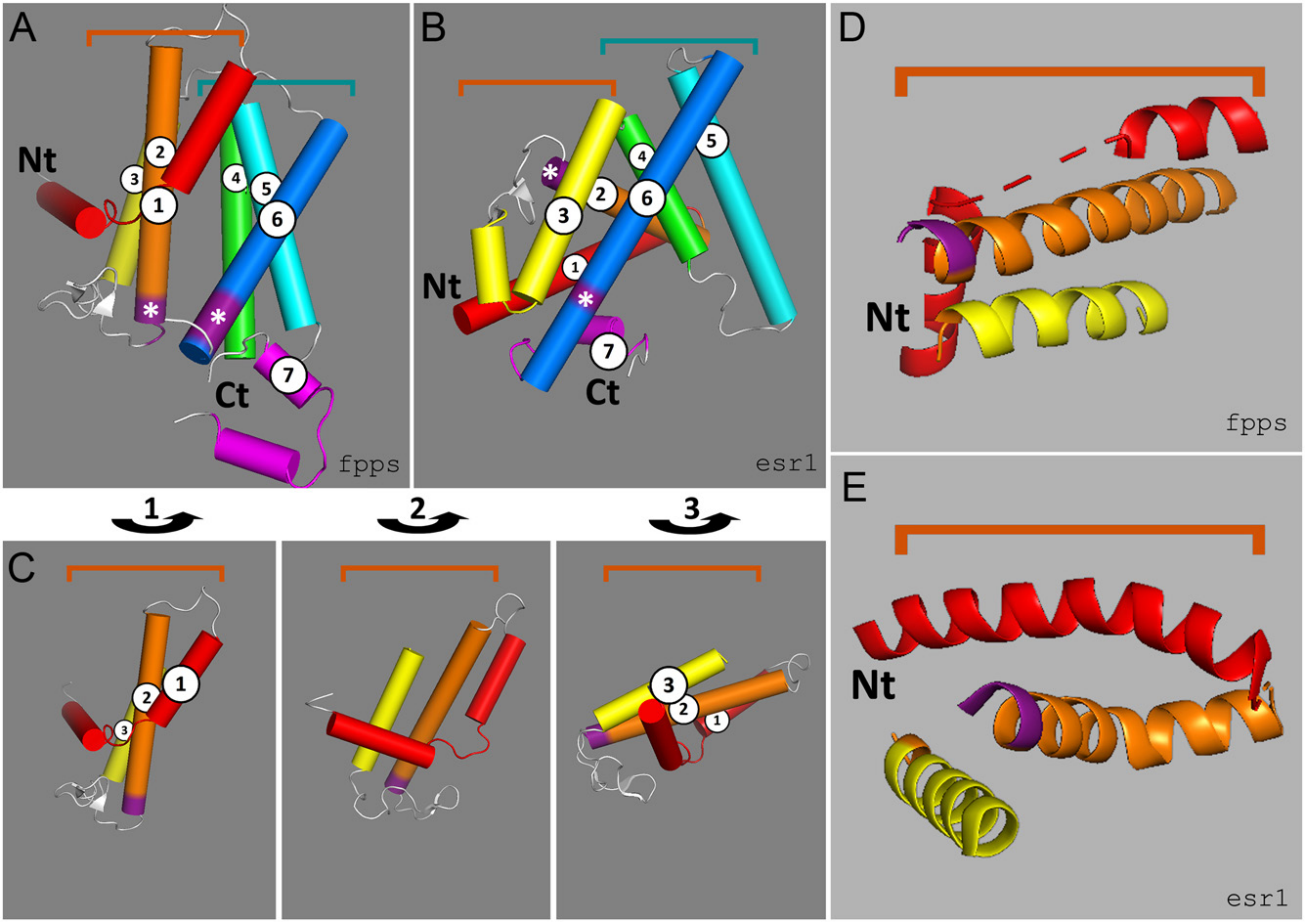
Structural distortion required to generate a nuclear receptor (NR) framework from a terpene synthase (TS) framework. (A, B, and C) The native core helices c1–c7 of *Gallus gallus* (chicken) farnesyl pyrophosphate synthase (FPPS, panel A) and human estrogen receptor α (ESR1, panel B) (structure details are given in Supplementary Table 1) colored sequentially from the N-terminus (Nt) to the C-terminus (Ct) red, orange, yellow, green, cyan, blue, magenta. Major contact points (asterisks) are colored deep violet. Helices c4–6 (green/blue) adopt a similar configuration in FPPS and ESR1 (A and B), whereas TS helices c1–c3 (red/orange/yellow) require (i) rotation through 115° (C) to generate a 3D structure similar to that of NRs (D and E); in addition, (ii) the c1 helix that is ‘broken’ in FPPS (D) is contiguous in ESR1 (E). Rotation of the TS N-terminus may be essential to accommodate (or may have been generated by) fusion to the DNA-binding domain (that may have been independently acquired).

Given suggestive evidence that NR LBDs might be related to TS enzymes, we addressed the degree of deformation required to overlap the structures of the two protein groups. Because few detailed structures are available for the ‘early’ NRs or for the NUS1/NOGOBR/DHDPPS subgroup of TS enzymes, we compared the archetypical TS, chicken FPPS, to human ESR1. This revealed that, although the core helices c4–c6 adopt a similar geometry in the two molecules, the positioning of helices c1–c3 is somewhat different (Fig. 4), and rotation of the N-terminal c1–c3 block by 115° around the *z* axis (as defined by the coordinate system) relative to the rest of the protein was necessary to maximize the 3D similarities between the two proteins without other distortion. Similar rotations were necessary for other TS/NR pairs examined (not presented), indicating that this is a general feature of the TS–NR transition. Further work will be necessary to determine whether this structural rotation is ‘NR-specific’ or represents a transient structure adopted by TS enzymes during substrate binding, catalysis, product ejection, and return to the ground state that has been exploited by NR LBDs.

We then compared primary sequence motifs within key structures. All TS enzymes contain a deep hydrophobic pocket generated by the cluster of α-helices that accommodates the (C5–C30 or more) hydrophobic terpene chain (Christianson 2017, Fujihashi*et al.* 2018) where the catalytic site at the ‘mouth’ of this pocket comprises the aspartate-rich (DDxxD) motif at the end of helix c2, followed by a flexible loop containing paired arginine (RR) residues (termed here the ‘catalytic loop’). These charged residues together generate the primary metal-binding active center of the enzyme.

Comparison of NR LBD and TS 3D structures, assisted by primary/secondary sequence alignment, confirmed that the same primary pocket is also present in NRs, but the DDxxD motif at the mouth has been replaced by one or more basic residues (Arg, Lys, or Gln) corresponding to Arg397 within the WRS motif in human ESR1 (Fig. 2 and Supplementary Fig. 9) – a major contact point for both steroids (e.g. estradiol) and TS ligands (e.g. FPP), as well as for charged residues in other NR ligands such as DAFA (Fig. 2 and Supplementary Fig. 9). In the DHDPPS subgroup, the DDxxD motif has also been replaced by a basic residue (compare Fig. 2 and Supplementary Fig. 9).

We speculate that one of the key primary sequence alterations in the transition between typical TS enzymes and NRs is replacement of the DDxxD catalytic motif at the end of helix c2 in TSs by a basic residue in NRs. This may tend to lock the ligand in the pocket. This could have taken place in two steps because some TS-related molecules, notably DHDPPS/NUS1/NOGOBR, contain a basic residue at this position (like NRs), noting that NUS1/NOGOBR has little enzymatic activity. Of interest, the sponge *Amphimedon queenslandica* has only two NRs (aqNR1 and aqNR2) (Bridgham*et al.* 2010); aqNR2 might represent a hybrid form because it contains both the key arginine and an adjacent DD motif (Bridgham*et al.* 2010) that is reminiscent of the adjacent aspartates in the DDxxD motif of typical TS enzymes (Fig. 2 and Supplementary Fig. 9).

## Discussion

These data potentially provide a new perspective on the evolution of NRs. It was previously conjectured that NR LBDs at the base of the Metazoa had no ligand (Escriva*et al.* 1997, 2000), or that terpenoids (Moore 1990) or fatty acids (Bridgham*et al.* 2010) were the ancestral ligands. However, our new evidence suggests that the prototypic NR could have been a modified TS enzyme that subsequently acquired a DBD, with terpenoid substrate or metabolite assuming the role of activating ligand. Of note, given extensive sequence and structural homologies that are shared by all members of the class, NRs are likely to have had a single evolutionary origin. We therefore infer that an ancestral TS enzyme (or close relative) may have acquired a DBD through fusion with another protein; this unique event probably occurred only once and then radiated through diverse processes including mutational changes to maximize function, species divergence, and possibly horizontal gene transfer. For these reasons, the parental form that provided the first NR LBD may have been an atypical or even mutant TS enzyme. Although our findings do not identify the exact forerunner to the NR LBD, they could argue that it was perhaps more closely related to the DHDPPS/NUS1/NOGOBR sub-branch of *cis*-isoprene transferase TS enzymes than to other members of the clan.

Although we contend that NRs are related to, and might potentially have descended from, the TS clan of enzymes, a legitimate counterargument could be that any protein with an appropriate number of α-helices would give a positive match using 3D comparison programs (FATCAT and jCE) that allow helices to be rearranged. However, this may not be valid. First, the scores generated take into account the degree of rearrangement required: although structure searching on the RCSB Protein Databank using FATCAT did detect some ‘similar’ structures, these were rated far lower than TS vs NR comparisons. Other enzyme groups built around a cluster of α-helices (e.g. cytochrome P450 enzymes and hydroxysteroid dehydrogenases) scored low in comparisons with NR LBDs.

In addition, although inferring similarities between distant protein families is fraught with uncertainties, FATCAT comparisons are carefully benchmarked by reference to comparisons of similar vs dissimilar structures to generate a probability function (*P* value) that accurately reflects the true likelihood that two protein structures might overlap fortuitously (Ye & Godzik 2004). The *P* values (that in our TS vs NR comparisons are in the range *P* = 0.015 to *P* = 0.004 for closest matches) thus provide an indication that the observed structural similarities are unlikely to occur by chance. Furthermore, some TS enzymes were rated to be closer to NR LBDs than to other members of the TS clan. Indeed, 3D comparisons are often employed to establish relatedness in cases where primary sequence similarities are insufficient (Jiang & Blouin 2007, Modi & Dunbrack Jr 2019, Leitão & Enguita 2021), and we found that separate 3D comparisons for TS enzymes and NR LBDs generated the same phylogenetic trees as those based on primary sequences.

Second, primary through tertiary structure comparisons, combined with crystal data and docking, point to close conservation of contact site locations and ligand placement within the polypeptide, which would be unexpected in unrelated proteins. Third, the observation that TS ligands such as FPP have robust interactions with NRs argues in favor of our hypothesis. Fourth, potential primary sequence similarities between ‘early’ NRs and the DHDPPS group of *cis*-isoprene transferases could argue for an evolutionary relationship, although convergent evolution remains a potential confounding factor in all such comparisons.

Finally, biological plausibility – steroids (the classic NR ligands) are polyterpenes, and it is reasonable to suspect that the protein framework that first evolved to metabolize terpenes could have given rise to a receptor for the same molecules. A different interpretation would require that NRs assembled from an entirely different protein structure, perhaps by chance. However, this would require simultaneous (instead of stepwise) acquisition of a DNA-binding domain; Ockham’s razor militates against this alternative hypothesis.

Interestingly, many NRs, including so-called orphan NRs, are reported to respond to the terpenoid precursor and TS substrate FPP (Das*et al.* 2007). Human ESR1 maintains the key Arg397 binding residue for FPP in the LBD, even though mutation of this residue to inhibit FPP binding did not abolish receptor activation by estradiol (Goyanka*et al.* 2010). Why has it not been lost? We speculate that basal low-level estrogen receptor activation may be maintained by FPP in states of physiological estrogen deficiency (e.g. in neonates).

In addition to evolutionary implications, the structural similarities we have identified between TSs and NRs could have pharmacological ramifications because molecules that target terpene biosynthesis potentially have collateral effects on NRs, and vice versa. A prime example is the bone-sparing TS inhibitor ZA that targets FPPS (Guo*et al.* 2007, Tsoumpra*et al.* 2015, Center*et al.* 2020) and, as shown here, crossreacts with ESR1, potentially explaining a secondary inhibitory effect of ZA on metastatic breast cancer (Winter*et al.* 2008, George*et al.* 2020). This could be an important factor to be considered in developing the next generation of terpenoid-based clinical drugs.

From a broader perspective, this work supports the concept that an enzyme group evolved and diversified to become a ligand-modulated transcriptional regulator. There are several examples in bacteria, yeast, and vertebrates where metabolic enzymes evolved to regulate transcription (Citron*et al.* 1992, Hall*et al.* 2004, Levati*et al.* 2016), but possible gating by substrate/product was not addressed. Indeed, if a TS to NR transition can be confirmed by other lines of evidence, this could represent the tip of an evolutionary iceberg in which enzymes more generally provided the primary ligand-binding site that subsequently evolved to become a ligand-regulated receptor. Previous analysis suggested that steroid-activated NRs coevolved *with* enzymes involved in ligand synthesis/metabolism (Baker 2011), whereas our analysis suggests that NRs could have evolved *from* such an enzyme. The distinction is more than semantic, given the centrality of terpene biosynthesis to NR biology.

Nevertheless, despite suggestive structural similarities between NR LBDs and *cis*-isoprene transferases in particular – that are unlikely to occur by chance – these do not unambiguously demonstrate a direct/close evolutionary relationship between the two protein classes. For example, it remains the case that our results do not formally exclude convergent evolution, underscoring the complexity of inferring relationships among distantly related proteins. We also speculate that a so far uncharacterized protein family could remain to be uncovered in pre-Metazoans that is intermediate in form and function between TS enzymes and NRs LBDs; our analysis may provide a framework for identifying such intermediate molecules.

In addition, other lines of evidence may need to be invoked. For example, as potential confirmation of a TS–NR transition, the question arises of whether some NRs might have residual catalytic activity. We have been unable to address this issue, but it is possible that LBDs of some extant NRs, notably aqNR2, might retain TS-like enzymatic activity. Further experiments will be necessary to address this question.

In conclusion, the data suggest that NR LBDs could potentially share an evolutionary origin, albeit distant, with the *cis*-isoprene transferase family of TS enzymes. If this can be confirmed, the emergence of NRs from a subclass of TS enzymes at the base of the Metazoan radiation would reframe the involvement of ancestral terpenoid molecules in morphogenesis, early development, and vertebrate evolution

## Supplementary Material

supplementary Table S1

Table S2. Compendium of distance scores from structural comparisons

supplementary Table S3

Sup fig 1: Phylogenetic tree generated by FATCATflexible + Fitch–Margoliash.

Sup fig 2: Tree generated by FATCATflexible + UPGMA.

Sup fig 3: Tree generated by FATCATflexible + Neighbor-Joining.

Sup fig 4: Tree generated by FATCATrigid + UPGMA.

Sup fig 5: Tree generated by jCE + UPGMA

Sup fig 6: Ligand overlap between Escherichia coli OPPS, PDB 3WJN; and Homo sapiens ESR, PDB 1QKU analyzed by (i) structure overlap, (ii) separate extraction of the overlapped polypeptides, (iii) docking of their respective ligands, and (iv) combining into a single file for visualization

Sup Figure 7. Detailed phylogenetic tree of cis-isoprene transferases and nuclear receptors

Sup Figure 8. Primary sequence alignments of NUS1 proteins and ligand-binding domains (LBDs) using M-coffee. NR LBDs (left), NUS1 sequences (center), and both (right) were aligned using Mcoffee; the three regions of putative conservation (1–3) are highlighted in red, green, and blue, respectively. Pink coloration on the alignments in the figure is generally considered to represent 'good' conservation, whereas yellow denotes 'average' conservation: the specificity of M-Coffee is that, instead of computing a multiple sequence alignment on its own, it uses other packages to compute the alignments, and then uses T-Coffee to combine all these alignments into one unique final alignment. The combined alignments are on average better than the initial alignments, and the regions where they agree tend to be correctly aligned (http://www.tcoffee.org/Projects/mcoffee/). The extent of amino acid 'similarity' over the three regions (1–3) between NR LBDs and NUS1 can be estimated to be (ranges: strict equivalent to flexible) 48–58%, 23–58%, and 26–44%, respectively, based on whether functionally similar amino acids are present (i.e., are permitted) at any given position, but this estimate depends on what constitutes functional similarity, and does not take into consideration the fact that NR LBDs had a unique origin, and possibly arose from a mutant or otherwise atypical protein whose sequence could have diverged from that of conventional NUS1 and related proteins. Prefixes for the aligned sequences are: aq, Amphimedon queenslandia; cb, Caenorhabditis brenneri; dr, Danio rerio; dv, Drosophila virilis; hs, Homo sapiens; il, Intoshia linei; of, Orbicella faveolata; sc, Saccharomyces cerevisiae; ta, Trichoplax adherens; ts, Trichoplax sp. H2; xt, Xenopus tropicalis.

Sup fig 9: Primary/secondary sequence alignment of key terpene sythase enzymes and nuclear receptors with docking interactions (Sup Table 3).

Supplementary Data S1

Supplementary Data S2

Supplementary Data S3

## Declaration of interest

The authors declare that there is no conflict of interest that could be perceived as prejudicing the impartiality of the research reported.

## Funding

This study did not receive any specific grant from any funding agency in the public, commercial, or not-for-profit sector.
