# Supplementary material for: Evidence that nuclear receptors are related to terpene synthases
Source: J Mol Endocrinol. 2022 Feb 3;68(3):153–66. doi: 10.1530/JME-21-0156 (PMC8942334; doi:10.1530/JME-21-0156)
Supplement: supplementary Table S1 [file supplementary_table_1.pdf]

Table S1. List of Terpene Synthases and Nuclear Receptors Protein Structures for 64 x 64 Matrix Comparison

**Enzyme/Receptor**	**Species**	**PDB**	**Short name for tree drawing**	TS/NR
Aristolochene synthases (I – sesquiterpene synthases)	P. roqueforti (fungi)	1DGP.A	prAS-TS	TS
α-Bisabolene synthase (I – sesquiterpene synthases)	A. grandis (plant)	3SAE.A	agBAS-TS	TS
(+)-D-Cadinene synthase (I – sesquiterpene synthases)	G. arboretum (plant)	3G4D.A	gaCS-TS	TS
5-Epi-aristolochene synthase (I – sesquiterpene synthases)	N. tabacum (plant)	5EAS.A	ntEAS-TS	TS
Abietadiene synthase (I – diterpene synthases/II – diterpene cyclases)	A. grandis (plant)	3S9V.A	agADS-TS	TS
Aristolochene synthase	Aspergillus terreus	4KWD.A	atACS-TS	TS
Aristolochene synthases (I – sesquiterpene synthases)	A. terreus (fungi)	2E4O.A	atAD-TS	TS
Bornyl diphosphate synthase (I – monoterpene synthases)	S. officinalis (plant)	1N1B.A	soBPPS-TS	TS
Cineole synthase (I – monoterpene synthases)	S. fruticosa (plant)	2J5C.A	sfCS-TS	TS
DAF-12	Ancylostoma ceylanicum	3UP3.A	acDAF12-NR	NR
Dehydrosqualene synthase	Staphylococcus aureus	3VJD.A	saDHSQS-TS	TS
Ecdysone receptor	Heliothis virescens	1R1K.D	hvECDR-NR	NR
Ecdysone receptor	Bemisia tabaci	1Z5X.E	btECDR-NR	NR
Ent-copalyl diphosphate synthase (II – diterpene cyclases)	A. thaliana (plant)	3PYA.A	atECPPS-TS	TS
Ent-kaurene synthase	Bradyrhizobium japonicum	4W4R.A	bjEKS-TS	TS
Epi-isozizaene synthase	Streptomyces coelilcolor	3KB9.A	scEIZS-TS	TS
ESR	Crassostrea gigas	4N1Y.A	cgESR-NR	NR
ESR1	Homo sapiens	2OCF.A	hsESR1-NR	NR
Farnesyl diphosphate synthetase	Homo sapiens	2VF6.A	hsFPPS1-TS	TS
FPP synthase (I – trans-IDS)	G. gallus (animal)	1FPS.A	ggFPPS-TS	TS
FPP synthase (I – trans-IDS)	E. coli (bacteria)	1RQI.A	ecFPPS-TS	TS
FPP synthase (I – trans-IDS)	S. aureus (bacteria)	1RTR.A	saFPPS-TS	TS
FPP synthase (I – trans-IDS)	H. sapiens (animal)	1YV5.A	hsFPPS2-TS	TS
Geranyltranstransferase	Methanosarcina mazei	3IPI.A	mmGTT-TS	TS
Germacradienol/geosmin synthase	Streptomyces coelicolor	5DZ2.A	scGGEOS-TS	TS
GGPP synthase (I – trans-IDS)	S. cerevisiae (fungi)	2DH4.A	scGGPPS-TS	TS
GGPP synthase (I – trans-IDS)	S. alba (plant)	2J1O.A	saGGPPS-TS	TS
GGPPS	Pyrococcus horikoshii	1WY0.A	phGGPPS-TS	TS
GGPPS	Homo sapiens	2Q80.A	hsGGPPS-TS	TS
GPP synthase (I – trans-IDS)	M. piperita (plant)	3KRA.A	mpGPPS-TS	TS
Hepatocyte nuclear factor HNF-1	H. sapiens	4IQR.A	hsHNF1-NR	NR
Heptaprenyl diphosphate synthase	Staphylococcus aureus	5H9D.A	saHPDS-TS	TS
Heptaprenyl diphosphate synthase (	Staphylococcus aureus	5H9D.C	saHEPPS-TS	TS
Hexaprenyl diphosphate synthase	Micrococcus luteus	3AQB.A	mlHXDS-TS	TS
Hexaprenyl pyrophosphate synthase (I – trans-IDS)	S. solfataricus (archaea)	2AZJ.A	ssHPPS-TS	TS
