# Supplementary material for: Evidence that nuclear receptors are related to terpene synthases
Source: J Mol Endocrinol. 2022 Feb 3;68(3):153–66. doi: 10.1530/JME-21-0156 (PMC8942334; doi:10.1530/JME-21-0156)
Supplement: Table S2. Compendium of distance scores from structural comparisons [file supplementary_table_2.pdf]

).

1)

x 64 Distance Matrices for

The data have been formatted to facilitate analysis by phylogenetic

The first column in each case gives the abridged name of the protein structure (TS, terpene
Correspondence to the Protein Database (PDB) structure reference numbers
In each case the identities of the 64 structures in the vertical column (top to bottom) are
horizontal direction (left
Tree-drawing programs generally require require that the horizontal

64

The three matrices give the distance values for
FL FATCATflex
RIG FATCATri
jC jCE
ME Mean values for FLEX,

In each case the primary output of the program was as a similarity index (raw score) that takes into
of
% of values were under 1000, and ~3% were over 1000 (very close matches): values >1000 were
Similarity values 0 to 1000 were first converted to values 0 to 1, and then inverted to distance scores
to 0
For typical phylogenetic analysis, the matrices plus identities (regions in color) should be entered
on a separate line by the number of categories (64)

(

Table S2. Compendium of distance scores from structural comparisons

…71	0.61	0.61	0.61	0.64	0.61	0.57	0.41	0.62	0.61	0.60	0.63	0.61	0.71	0.59	0.94	0.78	0.60	0.52	0.68	0.52	0.70	0.69	0.69	0.74	0.65	0.61	0.69	0.40	0.69	0.67	0.59	0.72	0.58	0.59	0.68	0.53	0.37	0.54	0.54	0.61	0.75	0.83	0.82	0.80	0.51	0.69
…59	0.52	0.51	0.56	0.58	0.50	0.54	0.44	0.53	0.59	0.53	0.54	0.56	0.68	0.53	0.94	0.77	0.57	0.00	0.36	0.00	0.67	0.67	0.66	0.72	0.60	0.59	0.65	0.49	0.65	0.67	0.57	0.68	0.57	0.56	0.35	0.00	0.44	0.49	0.48	0.58	0.76	0.81	0.81	0.78	0.00	0.70
…59	0.55	0.54	0.56	0.61	0.55	0.56	0.49	0.57	0.60	0.53	0.55	0.58	0.68	0.55	0.95	0.77	0.58	0.00	0.46	0.00	0.66	0.68	0.66	0.73	0.61	0.59	0.68	0.52	0.63	0.68	0.57	0.69	0.59	0.56	0.49	0.00	0.48	0.51	0.50	0.57	0.76	0.82	0.81	0.79	0.00	0.70
…59	0.53	0.54	0.56	0.61	0.54	0.55	0.45	0.57	0.60	0.53	0.55	0.58	0.68	0.55	0.94	0.76	0.57	0.00	0.49	0.00	0.65	0.66	0.64	0.73	0.61	0.59	0.67	0.47	0.62	0.69	0.57	0.69	0.58	0.57	0.50	0.00	0.44	0.48	0.48	0.59	0.76	0.81	0.81	0.79	0.00	0.69
…59	0.54	0.52	0.56	0.59	0.53	0.55	0.45	0.53	0.59	0.53	0.53	0.56	0.67	0.53	0.94	0.77	0.56	0.00	0.37	0.00	0.68	0.67	0.66	0.74	0.59	0.58	0.66	0.48	0.65	0.66	0.57	0.70	0.57	0.56	0.37	0.00	0.45	0.49	0.49	0.60	0.75	0.80	0.80	0.78	0.00	0.69
…71	0.59	0.60	0.60	0.62	0.59	0.58	0.38	0.61	0.61	0.58	0.62	0.61	0.70	0.58	0.94	0.78	0.59	0.51	0.67	0.49	0.70	0.67	0.68	0.74	0.63	0.60	0.67	0.38	0.67	0.66	0.58	0.71	0.57	0.58	0.67	0.52	0.34	0.53	0.52	0.62	0.76	0.83	0.83	0.79	0.47	0.69
…71	0.60	0.61	0.61	0.62	0.60	0.58	0.40	0.62	0.62	0.60	0.63	0.60	0.69	0.58	0.94	0.78	0.59	0.52	0.67	0.51	0.69	0.68	0.68	0.74	0.64	0.61	0.68	0.40	0.68	0.67	0.59	0.71	0.58	0.60	0.69	0.53	0.37	0.54	0.53	0.62	0.76	0.83	0.82	0.80	0.51	0.70
…59	0.54	0.53	0.57	0.62	0.53	0.57	0.49	0.57	0.60	0.53	0.56	0.59	0.69	0.58	0.94	0.77	0.58	0.00	0.48	0.00	0.67	0.68	0.67	0.73	0.62	0.58	0.65	0.52	0.66	0.68	0.58	0.70	0.59	0.57	0.49	0.00	0.45	0.51	0.50	0.58	0.76	0.81	0.79	0.78	0.00	0.70
…59	0.56	0.53	0.57	0.61	0.54	0.57	0.49	0.57	0.61	0.54	0.56	0.58	0.69	0.56	0.94	0.78	0.57	0.00	0.49	0.00	0.68	0.66	0.65	0.73	0.61	0.58	0.65	0.54	0.65	0.66	0.57	0.69	0.60	0.58	0.52	0.00	0.48	0.53	0.53	0.58	0.75	0.81	0.81	0.78	0.00	0.71
…61	0.72	0.71	0.70	0.71	0.72	0.71	0.69	0.72	0.70	0.71	0.72	0.69	0.62	0.70	0.94	0.80	0.70	0.70	0.74	0.70	0.72	0.59	0.59	0.66	0.72	0.70	0.73	0.70	0.71	0.74	0.69	0.62	0.70	0.71	0.76	0.71	0.69	0.70	0.70	0.70	0.67	0.83	0.83	0.81	0.69	0.54
…73	0.63	0.64	0.61	0.65	0.63	0.63	0.57	0.65	0.63	0.60	0.65	0.62	0.74	0.62	0.94	0.77	0.64	0.60	0.67	0.59	0.71	0.71	0.72	0.76	0.66	0.61	0.71	0.60	0.70	0.40	0.61	0.74	0.47	0.46	0.71	0.60	0.58	0.58	0.58	0.60	0.76	0.84	0.84	0.82	0.62	0.72
…63	0.68	0.68	0.68	0.68	0.68	0.69	0.67	0.68	0.70	0.68	0.67	0.68	0.53	0.69	0.94	0.80	0.69	0.67	0.71	0.68	0.71	0.53	0.52	0.58	0.71	0.68	0.73	0.70	0.71	0.72	0.70	0.50	0.70	0.69	0.74	0.68	0.70	0.68	0.68	0.68	0.59	0.83	0.84	0.82	0.67	0.46
…62	0.67	0.68	0.68	0.67	0.67	0.69	0.68	0.68	0.70	0.68	0.68	0.68	0.52	0.69	0.94	0.79	0.68	0.67	0.73	0.68	0.71	0.53	0.52	0.56	0.70	0.67	0.71	0.70	0.70	0.74	0.69	0.48	0.71	0.71	0.74	0.66	0.69	0.69	0.69	0.69	0.58	0.84	0.84	0.82	0.66	0.47
…71	0.58	0.57	0.60	0.63	0.57	0.62	0.60	0.57	0.63	0.55	0.61	0.62	0.69	0.58	0.94	0.79	0.62	0.12	0.37	0.11	0.68	0.69	0.69	0.76	0.64	0.61	0.68	0.60	0.65	0.66	0.61	0.71	0.62	0.60	0.37	0.00	0.60	0.58	0.57	0.62	0.76	0.81	0.81	0.81	0.16	0.68
…72	0.61	0.60	0.62	0.63	0.61	0.58	0.52	0.61	0.64	0.57	0.56	0.63	0.72	0.57	0.94	0.78	0.60	0.48	0.68	0.49	0.71	0.70	0.68	0.75	0.59	0.64	0.71	0.54	0.70	0.71	0.60	0.73	0.64	0.64	0.69	0.51	0.50	0.58	0.58	0.63	0.77	0.82	0.82	0.81	0.49	0.72
…59	0.56	0.58	0.57	0.61	0.56	0.54	0.22	0.54	0.61	0.53	0.55	0.60	0.68	0.51	0.94	0.77	0.59	0.42	0.64	0.42	0.69	0.66	0.65	0.73	0.59	0.59	0.68	0.31	0.67	0.68	0.54	0.68	0.51	0.53	0.64	0.45	0.26	0.47	0.47	0.60	0.74	0.82	0.81	0.80	0.42	0.69
…61	0.71	0.71	0.71	0.70	0.71	0.71	0.69	0.71	0.72	0.70	0.71	0.70	0.51	0.71	0.94	0.80	0.70	0.69	0.75	0.70	0.73	0.54	0.52	0.55	0.72	0.71	0.72	0.72	0.72	0.73	0.71	0.48	0.71	0.71	0.76	0.69	0.71	0.72	0.72	0.71	0.56	0.83	0.83	0.81	0.69	0.52
…60	0.71	0.70	0.71	0.70	0.70	0.70	0.69	0.71	0.71	0.71	0.70	0.70	0.52	0.69	0.93	0.81	0.71	0.68	0.73	0.69	0.72	0.55	0.53	0.59	0.72	0.70	0.72	0.71	0.72	0.74	0.70	0.51	0.72	0.71	0.74	0.69	0.71	0.71	0.71	0.70	0.59	0.83	0.82	0.79	0.69	0.54
…71	0.00	0.07	0.40	0.46	0.02	0.49	0.58	0.38	0.49	0.27	0.37	0.43	0.69	0.32	0.92	0.77	0.46	0.52	0.62	0.54	0.69	0.69	0.68	0.73	0.44	0.59	0.67	0.60	0.68	0.69	0.42	0.72	0.61	0.61	0.65	0.54	0.59	0.61	0.61	0.60	0.75	0.82	0.82	0.81	0.53	0.70
…70	0.07	0.00	0.41	0.44	0.07	0.48	0.58	0.39	0.48	0.29	0.40	0.41	0.70	0.35	0.92	0.76	0.44	0.52	0.62	0.52	0.68	0.69	0.68	0.73	0.44	0.59	0.67	0.60	0.66	0.68	0.43	0.72	0.62	0.60	0.64	0.51	0.59	0.60	0.60	0.61	0.75	0.82	0.82	0.81	0.54	0.71
…71	0.40	0.41	0.11	0.31	0.40	0.49	0.61	0.45	0.39	0.29	0.45	0.28	0.71	0.36	0.93	0.78	0.42	0.56	0.66	0.57	0.68	0.69	0.67	0.74	0.47	0.59	0.68	0.60	0.64	0.68	0.49	0.71	0.62	0.62	0.69	0.57	0.58	0.61	0.61	0.59	0.76	0.82	0.82	0.81	0.56	0.69
…70	0.46	0.44	0.31	0.18	0.46	0.44	0.63	0.46	0.39	0.36	0.51	0.29	0.69	0.41	0.94	0.77	0.44	0.59	0.69	0.61	0.69	0.69	0.69	0.75	0.48	0.61	0.70	0.64	0.66	0.68	0.52	0.69	0.65	0.65	0.70	0.59	0.63	0.64	0.64	0.62	0.75	0.83	0.83	0.80	0.61	0.70
…70	0.02	0.07	0.40	0.46	0.02	0.49	0.58	0.37	0.49	0.27	0.37	0.43	0.69	0.32	0.92	0.77	0.46	0.52	0.62	0.53	0.69	0.69	0.68	0.74	0.43	0.59	0.67	0.60	0.68	0.69	0.43	0.72	0.62	0.61	0.65	0.53	0.59	0.60	0.60	0.60	0.75	0.82	0.82	0.81	0.53	0.70
…70	0.49	0.48	0.49	0.44	0.49	0.21	0.57	0.52	0.44	0.38	0.52	0.43	0.71	0.39	0.94	0.75	0.43	0.54	0.66	0.55	0.68	0.68	0.67	0.75	0.51	0.55	0.68	0.58	0.66	0.68	0.46	0.71	0.60	0.59	0.67	0.55	0.55	0.55	0.55	0.58	0.76	0.81	0.81	0.79	0.54	0.70
…59	0.58	0.58	0.61	0.63	0.58	0.57	0.04	0.59	0.62	0.58	0.58	0.62	0.69	0.57	0.94	0.77	0.60	0.45	0.66	0.44	0.69	0.65	0.65	0.74	0.61	0.60	0.68	0.26	0.68	0.68	0.58	0.68	0.56	0.55	0.65	0.47	0.29	0.45	0.45	0.61	0.76	0.82	0.81	0.78	0.45	0.68
…71	0.38	0.39	0.45	0.46	0.37	0.52	0.59	0.04	0.56	0.35	0.29	0.46	0.69	0.41	0.94	0.77	0.49	0.56	0.62	0.56	0.70	0.69	0.69	0.75	0.43	0.58	0.68	0.62	0.67	0.69	0.49	0.72	0.61	0.60	0.67	0.57	0.59	0.61	0.61	0.59	0.76	0.83	0.83	0.79	0.56	0.71
…71	0.49	0.48	0.39	0.39	0.49	0.44	0.62	0.56	0.26	0.36	0.57	0.30	0.70	0.43	0.94	0.77	0.49	0.59	0.69	0.60	0.68	0.71	0.71	0.76	0.54	0.61	0.69	0.62	0.67	0.68	0.50	0.72	0.63	0.61	0.69	0.59	0.61	0.61	0.61	0.62	0.76	0.82	0.81	0.80	0.59	0.70
…71	0.27	0.29	0.29	0.36	0.27	0.38	0.58	0.35	0.36	0.04	0.35	0.28	0.69	0.20	0.92	0.78	0.38	0.52	0.63	0.52	0.68	0.68	0.69	0.75	0.39	0.57	0.68	0.59	0.65	0.67	0.40	0.71	0.61	0.60	0.66	0.53	0.56	0.59	0.58	0.60	0.75	0.81	0.81	0.78	0.52	0.69
…70	0.37	0.40	0.45	0.51	0.37	0.52	0.58	0.29	0.57	0.35	0.16	0.48	0.71	0.36	0.93	0.77	0.51	0.54	0.65	0.55	0.71	0.67	0.67	0.75	0.43	0.58	0.68	0.61	0.69	0.70	0.44	0.72	0.62	0.63	0.69	0.54	0.58	0.61	0.61	0.58	0.75	0.82	0.82	0.80	0.55	0.70
…70	0.43	0.41	0.28	0.29	0.43	0.43	0.62	0.46	0.30	0.28	0.48	0.18	0.70	0.36	0.94	0.77	0.42	0.56	0.67	0.56	0.67	0.70	0.68	0.74	0.46	0.61	0.68	0.62	0.64	0.67	0.48	0.71	0.62	0.61	0.68	0.58	0.60	0.60	0.60	0.59	0.75	0.82	0.81	0.79	0.57	0.70
…62	0.69	0.70	0.71	0.69	0.69	0.71	0.68	0.70	0.70	0.69	0.71	0.70	0.11	0.70	0.93	0.80	0.71	0.68	0.72	0.68	0.73	0.53	0.50	0.54	0.71	0.70	0.71	0.70	0.73	0.74	0.71	0.29	0.72	0.72	0.73	0.69	0.70	0.71	0.72	0.71	0.55	0.84	0.85	0.83	0.68	0.54
…59	0.32	0.35	0.36	0.41	0.32	0.39	0.57	0.41	0.43	0.20	0.36	0.36	0.70	0.06	0.94	0.78	0.41	0.54	0.65	0.54	0.71	0.69	0.69	0.73	0.38	0.57	0.68	0.57	0.69	0.67	0.35	0.71	0.57	0.58	0.67	0.54	0.56	0.60	0.60	0.60	0.75	0.82	0.80	0.79	0.54	0.70
…93	0.92	0.92	0.93	0.94	0.92	0.94	0.94	0.94	0.94	0.92	0.93	0.94	0.93	0.94	0.76	0.95	0.94	0.94	0.95	0.94	0.94	0.93	0.92	0.93	0.94	0.94	0.94	0.93	0.94	0.94	0.94	0.93	0.94	0.93	0.95	0.94	0.93	0.93	0.93	0.94	0.93	0.95	0.95	0.95	0.94	0.94
…81	0.77	0.76	0.78	0.77	0.77	0.75	0.77	0.77	0.77	0.78	0.77	0.77	0.80	0.78	0.95	0.62	0.76	0.77	0.80	0.77	0.80	0.80	0.79	0.81	0.75	0.77	0.79	0.78	0.79	0.78	0.77	0.82	0.77	0.78	0.82	0.77	0.78	0.78	0.78	0.77	0.83	0.86	0.86	0.83	0.77	0.81
…71	0.46	0.44	0.42	0.44	0.46	0.43	0.60	0.49	0.49	0.38	0.51	0.42	0.71	0.41	0.94	0.76	0.18	0.57	0.65	0.56	0.67	0.68	0.67	0.75	0.47	0.57	0.69	0.60	0.66	0.67	0.52	0.71	0.61	0.61	0.70	0.57	0.58	0.60	0.60	0.58	0.74	0.81	0.81	0.79	0.57	0.70
…68	0.52	0.52	0.56	0.59	0.52	0.54	0.45	0.56	0.59	0.52	0.54	0.56	0.68	0.54	0.94	0.77	0.57	0.00	0.45	0.00	0.66	0.65	0.64	0.73	0.58	0.56	0.65	0.50	0.63	0.67	0.56	0.68	0.58	0.57	0.49	0.00	0.45	0.49	0.49	0.57	0.75	0.81	0.80	0.78	0.00	0.69
…73	0.62	0.62	0.66	0.69	0.62	0.66	0.66	0.62	0.69	0.63	0.65	0.67	0.72	0.65	0.95	0.80	0.65	0.45	0.00	0.50	0.71	0.72	0.72	0.78	0.69	0.69	0.73	0.68	0.69	0.71	0.66	0.75	0.66	0.63	0.00	0.39	0.66	0.63	0.63	0.66	0.78	0.83	0.83	0.82	0.48	0.73
…59	0.54	0.52	0.57	0.61	0.53	0.55	0.44	0.56	0.60	0.52	0.55	0.56	0.68	0.54	0.94	0.77	0.56	0.00	0.50	0.00	0.66	0.67	0.65	0.74	0.59	0.57	0.64	0.51	0.64	0.67	0.56	0.69	0.58	0.57	0.51	0.00	0.43	0.49	0.49	0.57	0.74	0.81	0.81	0.78	0.00	0.70
…72	0.69	0.67	0.68	0.69	0.69	0.68	0.69	0.70	0.68	0.68	0.71	0.67	0.73	0.71	0.94	0.80	0.67	0.66	0.71	0.66	0.26	0.72	0.70	0.76	0.72	0.67	0.67	0.69	0.29	0.73	0.71	0.75	0.71	0.70	0.73	0.67	0.69	0.65	0.65	0.67	0.77	0.82	0.82	0.80	0.65	0.71
…55	0.69	0.69	0.69	0.69	0.69	0.68	0.65	0.69	0.71	0.68	0.67	0.70	0.53	0.69	0.93	0.80	0.68	0.65	0.72	0.67	0.72	0.30	0.34	0.58	0.70	0.69	0.71	0.68	0.71	0.75	0.70	0.51	0.71	0.70	0.75	0.66	0.68	0.69	0.69	0.66	0.59	0.84	0.83	0.82	0.66	0.54
…53	0.68	0.68	0.67	0.69	0.68	0.67	0.65	0.69	0.71	0.69	0.67	0.68	0.50	0.69	0.92	0.79	0.67	0.64	0.72	0.65	0.70	0.34	0.28	0.57	0.69	0.69	0.71	0.68	0.70	0.74	0.69	0.49	0.70	0.70	0.75	0.66	0.67	0.69	0.69	0.67	0.56	0.83	0.83	0.82	0.65	0.53
…59	0.73	0.73	0.74	0.75	0.74	0.75	0.74	0.75	0.76	0.75	0.75	0.74	0.54	0.73	0.93	0.81	0.75	0.73	0.78	0.74	0.76	0.58	0.57	0.42	0.76	0.74	0.74	0.76	0.75	0.77	0.74	0.50	0.75	0.74	0.81	0.71	0.74	0.74	0.74	0.72	0.54	0.85	0.85	0.84	0.73	0.58
…72	0.44	0.44	0.47	0.48	0.43	0.51	0.61	0.43	0.54	0.39	0.43	0.46	0.71	0.38	0.94	0.75	0.47	0.58	0.69	0.59	0.72	0.70	0.69	0.76	0.18	0.61	0.70	0.63	0.71	0.69	0.47	0.71	0.64	0.64	0.71	0.62	0.61	0.65	0.65	0.63	0.76	0.82	0.82	0.81	0.59	0.71
…70	0.59	0.59	0.59	0.61	0.59	0.55	0.60	0.58	0.61	0.57	0.58	0.61	0.70	0.57	0.94	0.77	0.57	0.56	0.69	0.57	0.67	0.69	0.69	0.74	0.61	0.18	0.68	0.60	0.65	0.69	0.56	0.71	0.62	0.62	0.71	0.61	0.60	0.57	0.57	0.43	0.75	0.82	0.82	0.78	0.58	0.71
…72	0.67	0.67	0.68	0.70	0.67	0.68	0.68	0.68	0.69	0.68	0.68	0.68	0.71	0.68	0.94	0.79	0.69	0.65	0.73	0.64	0.67	0.71	0.71	0.74	0.70	0.68	0.40	0.69	0.67	0.74	0.69	0.72	0.71	0.72	0.76	0.66	0.68	0.70	0.70	0.69	0.75	0.84	0.85	0.83	0.68	0.73
…71	0.60	0.60	0.60	0.64	0.60	0.58	0.26	0.62	0.62	0.59	0.61	0.62	0.70	0.57	0.93	0.78	0.60	0.50	0.68	0.51	0.69	0.68	0.68	0.76	0.63	0.60	0.69	0.11	0.68	0.69	0.56	0.72	0.59	0.57	0.70	0.52	0.31	0.49	0.49	0.62	0.76	0.82	0.81	0.81	0.48	0.72
…72	0.68	0.66	0.64	0.66	0.68	0.66	0.68	0.67	0.67	0.65	0.69	0.64	0.73	0.69	0.94	0.79	0.66	0.63	0.69	0.64	0.29	0.71	0.70	0.75	0.71	0.65	0.67	0.68	0.23	0.73	0.68	0.73	0.69	0.69	0.70	0.65	0.69	0.64	0.64	0.67	0.76	0.82	0.81	0.79	0.63	0.71
…74	0.69	0.68	0.68	0.68	0.69	0.68	0.68	0.69	0.68	0.67	0.70	0.67	0.74	0.67	0.94	0.78	0.67	0.67	0.71	0.67	0.73	0.75	0.74	0.77	0.69	0.69	0.74	0.69	0.73	0.28	0.67	0.75	0.60	0.57	0.74	0.67	0.68	0.66	0.66	0.69	0.78	0.84	0.83	0.81	0.67	0.75
…70	0.42	0.43	0.49	0.52	0.43	0.46	0.58	0.49	0.50	0.40	0.44	0.48	0.71	0.35	0.94	0.77	0.52	0.56	0.66	0.56	0.71	0.70	0.69	0.74	0.47	0.56	0.69	0.56	0.68	0.67	0.11	0.73	0.57	0.59	0.67	0.58	0.56	0.59	0.59	0.59	0.75	0.82	0.82	0.81	0.56	0.72
…61	0.72	0.72	0.71	0.69	0.72	0.71	0.68	0.72	0.72	0.71	0.72	0.71	0.29	0.71	0.93	0.82	0.71	0.68	0.75	0.69	0.75	0.51	0.49	0.50	0.71	0.71	0.72	0.71	0.73	0.75	0.73	0.14	0.71	0.72	0.76	0.68	0.70	0.72	0.72	0.71	0.53	0.84	0.84	0.83	0.69	0.52
…72	0.61	0.62	0.62	0.65	0.62	0.60	0.56	0.61	0.63	0.61	0.62	0.62	0.72	0.57	0.94	0.77	0.61	0.58	0.66	0.58	0.71	0.71	0.70	0.75	0.64	0.61	0.71	0.59	0.69	0.60	0.57	0.71	0.04	0.09	0.69	0.60	0.57	0.57	0.57	0.58	0.76	0.81	0.82	0.81	0.59	0.72
…71	0.61	0.60	0.62	0.65	0.61	0.59	0.55	0.60	0.61	0.61	0.63	0.61	0.73	0.58	0.93	0.78	0.61	0.57	0.63	0.57	0.70	0.70	0.70	0.74	0.64	0.62	0.72	0.57	0.69	0.57	0.59	0.72	0.09	0.00	0.66	0.58	0.55	0.57	0.57	0.59	0.76	0.81	0.80	0.80	0.56	0.71
…74	0.65	0.64	0.69	0.70	0.65	0.67	0.65	0.67	0.69	0.66	0.69	0.68	0.73	0.67	0.95	0.82	0.70	0.49	0.00	0.51	0.73	0.75	0.75	0.81	0.71	0.71	0.76	0.70	0.70	0.74	0.67	0.76	0.69	0.66	0.00	0.38	0.69	0.66	0.66	0.70	0.82	0.83	0.83	0.82	0.49	0.76
…59	0.54	0.51	0.57	0.59	0.53	0.55	0.47	0.57	0.59	0.53	0.54	0.58	0.69	0.54	0.94	0.77	0.57	0.00	0.39	0.00	0.67	0.66	0.66	0.71	0.62	0.61	0.66	0.52	0.65	0.67	0.58	0.68	0.60	0.58	0.38	0.00	0.47	0.53	0.53	0.61	0.76	0.81	0.80	0.80	0.00	0.69
…71	0.59	0.59	0.58	0.63	0.59	0.55	0.29	0.59	0.61	0.56	0.58	0.60	0.71	0.56	0.93	0.78	0.58	0.45	0.66	0.43	0.69	0.68	0.67	0.74	0.61	0.60	0.68	0.31	0.69	0.68	0.56	0.70	0.57	0.55	0.69	0.47	0.00	0.47	0.47	0.61	0.76	0.83	0.82	0.81	0.44	0.70
…71	0.61	0.60	0.61	0.64	0.60	0.55	0.45	0.61	0.61	0.59	0.61	0.60	0.71	0.60	0.93	0.78	0.60	0.49	0.63	0.49	0.65	0.69	0.69	0.74	0.65	0.57	0.70	0.49	0.64	0.66	0.59	0.72	0.57	0.57	0.66	0.53	0.47	0.00	0.00	0.60	0.74	0.82	0.82	0.81	0.48	0.69
…71	0.61	0.60	0.61	0.64	0.60	0.55	0.45	0.61	0.61	0.58	0.61	0.60	0.72	0.60	0.93	0.78	0.60	0.49	0.63	0.49	0.65	0.69	0.69	0.74	0.65	0.57	0.70	0.49	0.64	0.66	0.59	0.72	0.57	0.57	0.66	0.53	0.47	0.00	0.00	0.60	0.74	0.82	0.82	0.81	0.48	0.69
…70	0.60	0.61	0.59	0.62	0.60	0.58	0.61	0.59	0.62	0.60	0.58	0.59	0.71	0.60	0.95	0.77	0.58	0.58	0.66	0.57	0.67	0.66	0.67	0.72	0.63	0.43	0.69	0.62	0.67	0.69	0.59	0.71	0.58	0.59	0.70	0.61	0.61	0.60	0.60	0.18	0.74	0.81	0.81	0.80	0.58	0.69
…59	0.75	0.75	0.76	0.75	0.75	0.76	0.76	0.76	0.76	0.75	0.75	0.75	0.55	0.75	0.93	0.83	0.74	0.75	0.78	0.74	0.77	0.59	0.56	0.54	0.76	0.75	0.75	0.76	0.76	0.78	0.75	0.53	0.76	0.76	0.82	0.76	0.76	0.74	0.74	0.74	0.42	0.85	0.85	0.84	0.76	0.58
…83	0.82	0.82	0.82	0.83	0.82	0.81	0.82	0.83	0.82	0.81	0.82	0.82	0.84	0.82	0.95	0.86	0.81	0.81	0.83	0.81	0.82	0.84	0.83	0.85	0.82	0.82	0.84	0.83	0.82	0.84	0.82	0.84	0.81	0.81	0.83	0.81	0.83	0.82	0.82	0.81	0.85	0.35	0.42	0.49	0.81	0.84
…82	0.82	0.82	0.82	0.83	0.82	0.81	0.81	0.83	0.81	0.81	0.82	0.81	0.85	0.80	0.95	0.86	0.81	0.80	0.83	0.81	0.82	0.83	0.83	0.85	0.82	0.82	0.85	0.81	0.80	0.83	0.82	0.84	0.82	0.80	0.83	0.80	0.82	0.82	0.82	0.81	0.85	0.42	0.38	0.51	0.81	0.84
…79	0.81	0.81	0.81	0.80	0.81	0.79	0.78	0.79	0.80	0.79	0.80	0.79	0.83	0.79	0.95	0.83	0.79	0.77	0.82	0.78	0.80	0.82	0.82	0.84	0.81	0.78	0.83	0.81	0.79	0.81	0.81	0.83	0.81	0.80	0.82	0.80	0.81	0.81	0.81	0.80	0.84	0.49	0.51	0.33	0.79	0.82
…59	0.53	0.54	0.56	0.61	0.53	0.54	0.45	0.56	0.59	0.52	0.55	0.57	0.68	0.54	0.94	0.77	0.57	0.00	0.48	0.00	0.65	0.66	0.65	0.73	0.59	0.58	0.68	0.48	0.63	0.67	0.56	0.69	0.58	0.56	0.49	0.00	0.44	0.48	0.48	0.58	0.76	0.81	0.81	0.79	0.00	0.69
…54	0.70	0.71	0.69	0.70	0.70	0.70	0.68	0.71	0.70	0.69	0.70	0.70	0.54	0.70	0.94	0.81	0.70	0.69	0.73	0.70	0.71	0.54	0.53	0.58	0.71	0.71	0.73	0.72	0.71	0.75	0.72	0.52	0.72	0.71	0.76	0.70	0.70	0.69	0.69	0.69	0.58	0.84	0.84	0.82	0.69	0.30

[illegible]	0.62	0.63	0.62	0.64	0.62	0.57	0.41	0.66	0.61	0.60	0.63	0.61	0.73	0.59	0.94	0.80	0.61	0.52	0.70	0.52	0.72	0.70	0.70	0.76	0.66	0.62	0.70	0.40	0.70	0.68	0.59	0.74	0.60	0.60	0.72	0.54	0.37	0.54	0.54	0.62	0.76	0.84	0.83	0.81	0.51	0.71
[illegible]	0.52	0.53	0.56	0.59	0.52	0.55	0.44	0.54	0.60	0.53	0.54	0.57	0.70	0.53	0.94	0.78	0.58	0.00	0.40	0.00	0.69	0.68	0.67	0.73	0.60	0.61	0.65	0.50	0.67	0.67	0.57	0.74	0.57	0.56	0.36	0.00	0.44	0.49	0.49	0.60	0.77	0.84	0.83	0.80	0.00	0.71
[illegible]	0.56	0.56	0.57	0.62	0.56	0.56	0.49	0.57	0.61	0.55	0.55	0.58	0.71	0.56	0.95	0.77	0.58	0.00	0.52	0.00	0.69	0.69	0.67	0.73	0.61	0.60	0.69	0.53	0.67	0.69	0.59	0.72	0.60	0.56	0.52	0.00	0.48	0.51	0.50	0.58	0.77	0.83	0.83	0.81	0.00	0.70
[illegible]	0.55	0.56	0.56	0.61	0.55	0.56	0.45	0.57	0.61	0.55	0.56	0.59	0.70	0.56	0.94	0.77	0.58	0.00	0.53	0.00	0.68	0.66	0.65	0.73	0.61	0.60	0.68	0.47	0.66	0.69	0.57	0.73	0.58	0.58	0.54	0.00	0.44	0.48	0.48	0.59	0.76	0.82	0.82	0.81	0.00	0.70
[illegible]	0.55	0.55	0.56	0.60	0.54	0.56	0.45	0.54	0.60	0.54	0.54	0.57	0.68	0.53	0.94	0.77	0.56	0.00	0.40	0.00	0.70	0.68	0.68	0.74	0.59	0.59	0.67	0.49	0.67	0.66	0.57	0.73	0.57	0.56	0.39	0.00	0.45	0.50	0.49	0.60	0.76	0.83	0.83	0.82	0.00	0.70
[illegible]	0.60	0.60	0.60	0.62	0.60	0.58	0.38	0.64	0.61	0.58	0.62	0.61	0.73	0.58	0.94	0.78	0.60	0.51	0.70	0.49	0.72	0.67	0.68	0.75	0.63	0.61	0.69	0.38	0.70	0.67	0.58	0.72	0.58	0.59	0.73	0.53	0.34	0.53	0.53	0.62	0.76	0.84	0.84	0.80	0.47	0.69
[illegible]	0.61	0.62	0.61	0.62	0.62	0.58	0.40	0.66	0.62	0.60	0.63	0.61	0.74	0.58	0.94	0.80	0.61	0.53	0.70	0.51	0.70	0.68	0.69	0.75	0.65	0.62	0.69	0.41	0.68	0.68	0.59	0.74	0.60	0.61	0.73	0.54	0.37	0.54	0.54	0.62	0.77	0.84	0.84	0.82	0.51	0.71
[illegible]	0.55	0.55	0.57	0.63	0.55	0.58	0.49	0.57	0.62	0.55	0.56	0.59	0.72	0.58	0.94	0.77	0.58	0.00	0.53	0.00	0.70	0.68	0.68	0.73	0.62	0.60	0.65	0.52	0.67	0.69	0.59	0.73	0.60	0.58	0.54	0.00	0.45	0.51	0.51	0.59	0.76	0.83	0.81	0.80	0.00	0.71
[illegible]	0.56	0.56	0.57	0.61	0.55	0.58	0.49	0.57	0.62	0.54	0.56	0.58	0.71	0.57	0.94	0.78	0.58	0.00	0.54	0.00	0.70	0.66	0.65	0.73	0.61	0.59	0.66	0.55	0.68	0.66	0.57	0.72	0.61	0.59	0.56	0.00	0.48	0.54	0.54	0.58	0.77	0.82	0.82	0.81	0.00	0.71
[illegible]	0.72	0.71	0.71	0.72	0.72	0.72	0.69	0.73	0.71	0.72	0.73	0.70	0.62	0.72	0.94	0.81	0.71	0.71	0.77	0.72	0.74	0.60	0.59	0.66	0.74	0.71	0.74	0.72	0.73	0.75	0.72	0.62	0.73	0.72	0.79	0.72	0.71	0.73	0.73	0.70	0.67	0.85	0.85	0.84	0.71	0.54
[illegible]	0.63	0.64	0.62	0.65	0.63	0.63	0.57	0.66	0.63	0.61	0.66	0.62	0.75	0.62	0.94	0.77	0.64	0.61	0.71	0.60	0.73	0.72	0.72	0.76	0.67	0.62	0.72	0.61	0.71	0.40	0.62	0.75	0.49	0.46	0.72	0.60	0.58	0.59	0.59	0.60	0.76	0.85	0.86	0.84	0.62	0.73
[illegible]	0.69	0.69	0.68	0.68	0.69	0.69	0.68	0.68	0.70	0.68	0.69	0.69	0.53	0.70	0.94	0.81	0.69	0.67	0.75	0.68	0.72	0.53	0.53	0.58	0.73	0.71	0.73	0.70	0.71	0.75	0.71	0.50	0.72	0.72	0.76	0.69	0.71	0.69	0.69	0.68	0.59	0.86	0.87	0.84	0.67	0.46
[illegible]	0.67	0.69	0.69	0.69	0.67	0.69	0.69	0.69	0.70	0.69	0.69	0.69	0.52	0.70	0.94	0.79	0.69	0.67	0.75	0.68	0.72	0.53	0.52	0.56	0.71	0.69	0.71	0.70	0.71	0.75	0.72	0.48	0.73	0.73	0.76	0.67	0.71	0.70	0.70	0.69	0.58	0.85	0.86	0.84	0.67	0.47
[illegible]	0.60	0.60	0.62	0.64	0.59	0.64	0.60	0.59	0.66	0.58	0.64	0.64	0.70	0.62	0.94	0.80	0.63	0.13	0.41	0.11	0.69	0.69	0.69	0.76	0.65	0.63	0.70	0.60	0.67	0.70	0.65	0.74	0.63	0.61	0.40	0.00	0.60	0.60	0.60	0.63	0.76	0.84	0.84	0.84	0.17	0.71
[illegible]	0.61	0.61	0.62	0.63	0.61	0.58	0.52	0.63	0.64	0.57	0.57	0.63	0.74	0.58	0.94	0.78	0.60	0.49	0.70	0.50	0.73	0.70	0.69	0.76	0.59	0.64	0.71	0.54	0.72	0.71	0.60	0.74	0.64	0.64	0.72	0.52	0.50	0.60	0.60	0.64	0.77	0.84	0.82	0.82	0.50	0.73
[illegible]	0.56	0.58	0.57	0.61	0.56	0.54	0.22	0.57	0.61	0.54	0.55	0.60	0.72	0.51	0.94	0.78	0.60	0.42	0.68	0.42	0.72	0.66	0.65	0.73	0.59	0.59	0.69	0.31	0.70	0.68	0.54	0.72	0.51	0.54	0.69	0.45	0.26	0.47	0.47	0.60	0.75	0.83	0.83	0.80	0.43	0.69
[illegible]	0.71	0.71	0.71	0.70	0.71	0.71	0.71	0.71	0.73	0.71	0.71	0.71	0.51	0.71	0.94	0.81	0.70	0.70	0.77	0.71	0.75	0.54	0.52	0.55	0.73	0.71	0.72	0.73	0.74	0.75	0.73	0.48	0.72	0.72	0.79	0.71	0.71	0.72	0.72	0.71	0.56	0.84	0.85	0.84	0.71	0.52
[illegible]	0.71	0.71	0.72	0.71	0.72	0.70	0.70	0.72	0.73	0.71	0.71	0.72	0.53	0.71	0.93	0.81	0.71	0.71	0.76	0.72	0.73	0.55	0.53	0.59	0.73	0.71	0.73	0.72	0.73	0.75	0.72	0.51	0.73	0.72	0.76	0.71	0.71	0.72	0.72	0.71	0.59	0.84	0.85	0.82	0.70	0.54
[illegible]	0.00	0.07	0.40	0.46	0.02	0.49	0.58	0.40	0.49	0.27	0.37	0.43	0.72	0.32	0.92	0.77	0.46	0.53	0.67	0.54	0.71	0.70	0.68	0.74	0.44	0.60	0.67	0.61	0.69	0.70	0.42	0.73	0.63	0.63	0.69	0.54	0.60	0.62	0.62	0.61	0.75	0.86	0.85	0.82	0.54	0.71
[illegible]	0.07	0.00	0.41	0.44	0.07	0.48	0.58	0.40	0.48	0.29	0.40	0.41	0.73	0.35	0.93	0.77	0.45	0.54	0.67	0.54	0.70	0.70	0.68	0.74	0.44	0.61	0.67	0.61	0.68	0.68	0.43	0.73	0.64	0.62	0.69	0.52	0.59	0.61	0.61	0.62	0.75	0.85	0.84	0.83	0.56	0.71
[illegible]	0.40	0.41	0.11	0.31	0.40	0.49	0.61	0.45	0.39	0.29	0.45	0.28	0.72	0.36	0.93	0.78	0.42	0.56	0.70	0.57	0.71	0.69	0.67	0.74	0.47	0.59	0.68	0.60	0.67	0.68	0.49	0.73	0.63	0.62	0.72	0.59	0.58	0.61	0.62	0.59	0.76	0.85	0.84	0.83	0.57	0.70
[illegible]	0.46	0.44	0.31	0.18	0.46	0.44	0.63	0.49	0.39	0.36	0.51	0.29	0.72	0.41	0.94	0.77	0.44	0.59	0.72	0.61	0.71	0.70	0.69	0.75	0.48	0.62	0.71	0.65	0.68	0.69	0.52	0.73	0.66	0.66	0.73	0.60	0.63	0.65	0.65	0.62	0.75	0.86	0.86	0.83	0.61	0.71
[illegible]	0.02	0.07	0.40	0.46	0.02	0.49	0.58	0.38	0.49	0.27	0.37	0.43	0.72	0.32	0.92	0.77	0.47	0.53	0.67	0.54	0.71	0.70	0.68	0.74	0.43	0.60	0.67	0.61	0.70	0.70	0.43	0.73	0.63	0.63	0.69	0.53	0.60	0.62	0.61	0.61	0.75	0.86	0.85	0.83	0.54	0.71
[illegible]	0.49	0.48	0.49	0.44	0.49	0.21	0.58	0.55	0.44	0.38	0.52	0.43	0.72	0.39	0.95	0.75	0.43	0.55	0.69	0.57	0.73	0.69	0.68	0.75	0.51	0.56	0.68	0.58	0.71	0.68	0.46	0.72	0.60	0.59	0.71	0.56	0.56	0.55	0.55	0.58	0.76	0.83	0.82	0.82	0.55	0.71
[illegible]	0.58	0.58	0.61	0.63	0.58	0.58	0.04	0.61	0.62	0.59	0.59	0.62	0.71	0.58	0.94	0.78	0.61	0.45	0.68	0.44	0.71	0.65	0.65	0.74	0.61	0.61	0.70	0.26	0.70	0.69	0.58	0.71	0.56	0.55	0.70	0.47	0.29	0.45	0.45	0.61	0.76	0.83	0.84	0.79	0.45	0.69
[illegible]	0.40	0.40	0.45	0.49	0.38	0.55	0.61	0.04	0.57	0.35	0.29	0.49	0.71	0.43	0.94	0.78	0.50	0.57	0.65	0.57	0.71	0.70	0.70	0.76	0.45	0.58	0.68	0.65	0.68	0.71	0.50	0.73	0.64	0.63	0.70	0.57	0.61	0.64	0.64	0.59	0.76	0.84	0.84	0.82	0.56	0.71
[illegible]	0.49	0.48	0.39	0.39	0.49	0.44	0.62	0.57	0.26	0.36	0.57	0.30	0.74	0.43	0.94	0.77	0.49	0.60	0.72	0.61	0.72	0.72	0.72	0.77	0.54	0.61	0.71	0.64	0.70	0.68	0.50	0.75	0.64	0.62	0.73	0.61	0.61	0.61	0.62	0.62	0.77	0.84	0.84	0.83	0.60	0.71
[illegible]	0.27	0.29	0.29	0.36	0.27	0.38	0.59	0.35	0.36	0.04	0.35	0.28	0.72	0.20	0.93	0.78	0.39	0.54	0.65	0.54	0.70	0.69	0.69	0.75	0.39	0.59	0.69	0.60	0.67	0.67	0.40	0.73	0.62	0.61	0.69	0.54	0.56	0.59	0.58	0.60	0.75	0.84	0.84	0.80	0.54	0.71
[illegible]	0.37	0.40	0.45	0.51	0.37	0.52	0.59	0.29	0.57	0.35	0.16	0.48	0.73	0.36	0.94	0.78	0.51	0.54	0.68	0.55	0.74	0.68	0.68	0.76	0.44	0.58	0.68	0.62	0.71	0.70	0.45	0.74	0.63	0.63	0.72	0.55	0.59	0.61	0.61	0.59	0.75	0.84	0.84	0.82	0.55	0.71
[illegible]	0.43	0.41	0.28	0.29	0.43	0.43	0.62	0.49	0.30	0.28	0.48	0.18	0.73	0.36	0.94	0.77	0.42	0.56	0.69	0.58	0.69	0.70	0.68	0.75	0.46	0.61	0.69	0.63	0.67	0.67	0.48	0.73	0.63	0.62	0.71	0.59	0.60	0.60	0.60	0.60	0.75	0.85	0.85	0.82	0.58	0.71
[illegible]	0.72	0.73	0.72	0.72	0.72	0.72	0.71	0.71	0.74	0.72	0.73	0.73	0.11	0.72	0.93	0.81	0.71	0.70	0.76	0.70	0.75	0.54	0.50	0.54	0.73	0.73	0.73	0.73	0.75	0.76	0.75	0.38	0.74	0.73	0.78	0.70	0.73	0.72	0.72	0.73	0.55	0.86	0.86	0.85	0.70	0.54
[illegible]	0.32	0.35	0.36	0.41	0.32	0.39	0.58	0.43	0.43	0.20	0.36	0.36	0.72	0.06	0.94	0.78	0.42	0.55	0.68	0.55	0.74	0.69	0.70	0.73	0.38	0.57	0.69	0.58	0.72	0.68	0.35	0.73	0.58	0.59	0.69	0.55	0.56	0.60	0.60	0.60	0.75	0.83	0.83	0.81	0.55	0.70
[illegible]	0.92	0.92	0.93	0.94	0.92	0.94	0.94	0.94	0.94	0.92	0.94	0.94	0.93	0.94	0.76	0.95	0.94	0.94	0.95	0.94	0.95	0.93	0.92	0.93	0.94	0.94	0.94	0.93	0.95	0.94	0.94	0.93	0.94	0.93	0.95	0.94	0.93	0.93	0.93	0.94	0.93	0.95	0.95	0.95	0.95	0.94
[illegible]	0.77	0.77	0.78	0.77	0.77	0.75	0.78	0.78	0.77	0.78	0.78	0.77	0.81	0.78	0.95	0.62	0.76	0.77	0.80	0.77	0.83	0.81	0.81	0.82	0.75	0.77	0.79	0.79	0.82	0.78	0.77	0.82	0.79	0.79	0.83	0.78	0.78	0.79	0.79	0.78	0.83	0.88	0.87	0.86	0.77	0.82
[illegible]	0.46	0.45	0.42	0.44	0.47	0.43	0.61	0.50	0.49	0.39	0.51	0.42	0.71	0.42	0.94	0.76	0.18	0.57	0.68	0.57	0.71	0.69	0.67	0.75	0.47	0.57	0.69	0.60	0.70	0.67	0.52	0.71	0.62	0.62	0.73	0.60	0.58	0.60	0.60	0.59	0.74	0.81	0.83	0.80	0.57	0.70
[illegible]	0.53	0.54	0.56	0.59	0.53	0.55	0.45	0.57	0.60	0.54	0.54	0.56	0.70	0.55	0.94	0.77	0.57	0.00	0.50	0.00	0.68	0.65	0.65	0.73	0.58	0.58	0.65	0.50	0.65	0.68	0.57	0.72	0.58	0.58	0.52	0.00	0.45	0.50	0.50	0.58	0.75	0.82	0.82	0.80	0.00	0.70
[illegible]	0.67	0.67	0.69	0.72	0.67	0.69	0.68	0.65	0.72	0.65	0.68	0.69	0.76	0.68	0.95	0.80	0.68	0.50	0.00	0.53	0.75	0.74	0.75	0.80	0.71	0.70	0.74	0.69	0.72	0.74	0.69	0.79	0.69	0.66	0.00	0.41	0.68	0.65	0.65	0.70	0.80	0.86	0.86	0.84	0.54	0.75
[illegible]	0.54	0.54	0.57	0.61	0.54	0.57	0.44	0.57	0.61	0.54	0.55	0.58	0.71	0.55	0.94	0.77	0.57	0.00	0.53	0.00	0.69	0.67	0.66	0.74	0.60	0.58	0.66	0.52	0.66	0.68	0.56	0.73	0.59	0.57	0.55	0.00	0.43	0.50	0.49	0.59	0.75	0.83	0.83	0.80	0.00	0.70
[illegible]	0.71	0.70	0.71	0.71	0.71	0.73	0.71	0.71	0.72	0.70	0.74	0.69	0.75	0.74	0.95	0.83	0.71	0.68	0.75	0.69	0.26	0.74	0.73	0.77	0.74	0.70	0.68	0.73	0.29	0.76	0.74	0.76	0.74	0.73	0.75	0.70	0.72	0.68	0.68	0.70	0.78	0.86	0.85	0.83	0.67	0.74
[illegible]	0.70	0.70	0.69	0.70	0.70	0.69	0.65	0.70	0.72	0.69	0.68	0.70	0.54	0.69	0.93	0.81	0.69	0.65	0.74	0.67	0.74	0.30	0.34	0.58	0.72	0.70	0.72	0.69	0.73	0.76	0.72	0.51	0.72	0.71	0.77	0.68	0.68	0.70	0.70	0.66	0.59	0.84	0.85	0.83	0.66	0.54
[illegible]	0.68	0.68	0.67	0.69	0.68	0.68	0.65	0.70	0.72	0.69	0.68	0.68	0.50	0.70	0.92	0.81	0.67	0.65	0.75	0.66	0.73	0.34	0.28	0.57	0.70	0.69	0.71	0.69	0.72	0.75	0.71	0.49	0.72	0.70	0.77	0.67	0.67	0.69	0.69	0.67	0.56	0.83	0.84	0.82	0.65	0.54
[illegible]	0.74	0.74	0.74	0.75	0.74	0.75	0.74	0.76	0.77	0.75	0.76	0.75	0.54	0.73	0.93	0.82	0.75	0.73	0.80	0.74	0.77	0.58	0.57	0.42	0.76	0.75	0.74	0.77	0.76	0.77	0.75	0.50	0.76	0.75	0.82	0.73	0.74	0.74	0.74	0.72	0.54	0.86	0.86	0.86	0.73	0.58
[illegible]	0.44	0.44	0.47	0.48	0.43	0.51	0.61	0.45	0.54	0.39	0.44	0.46	0.73	0.38	0.94	0.75	0.47	0.58	0.71	0.60	0.74	0.72	0.70	0.76	0.18	0.61	0.70	0.64	0.73	0.70	0.47	0.75	0.64	0.64	0.71	0.62	0.61	0.65	0.66	0.64	0.76	0.84	0.84	0.83	0.59	0.72
[illegible]	0.60	0.61	0.59	0.62	0.60	0.56	0.61	0.58	0.61	0.59	0.58	0.61	0.73	0.57	0.94	0.77	0.57	0.58	0.70	0.59	0.70	0.70	0.69	0.75	0.61	0.18	0.68	0.61	0.67	0.69	0.56	0.73	0.63	0.63	0.73	0.61	0.61	0.62	0.62	0.43	0.75	0.84	0.83	0.80	0.59	0.73
[illegible]	0.67	0.67	0.68	0.71	0.67	0.68	0.70	0.68	0.71	0.69	0.68	0.69	0.73	0.69	0.94	0.79	0.69	0.65	0.74	0.66	0.68	0.72	0.71	0.74	0.70	0.68	0.40	0.70	0.68	0.74	0.69	0.72	0.72	0.72	0.76	0.67	0.70	0.72	0.72	0.69	0.75	0.87	0.86	0.85	0.68	0.73
[illegible]	0.61	0.61	0.60	0.65	0.61	0.58	0.26	0.65	0.64	0.60	0.62	0.63	0.73	0.58	0.93	0.79	0.60	0.50	0.69	0.52	0.73	0.69	0.69	0.77	0.64	0.61	0.70	0.11	0.72	0.70	0.57	0.73	0.59	0.58	0.72	0.52	0.31	0.49	0.49	0.63	0.77	0.86	0.85	0.83	0.49	0.72
[illegible]	0.69	0.68	0.67	0.68	0.70	0.71	0.70	0.68	0.70	0.67	0.71	0.67	0.75	0.72	0.95	0.82	0.70	0.65	0.72	0.66	0.29	0.73	0.72	0.76	0.73	0.67	0.68	0.72	0.23	0.75	0.72	0.75	0.74	0.72	0.73	0.67	0.72	0.66	0.66	0.69	0.77	0.85	0.84	0.83	0.64	0.72
[illegible]	0.70	0.68	0.68	0.69	0.70	0.68	0.69	0.71	0.68	0.67	0.70	0.67	0.76	0.68	0.94	0.78	0.67	0.68	0.74	0.68	0.76	0.76	0.75	0.77	0.70	0.69	0.74	0.70	0.75	0.28	0.67	0.77	0.60	0.57	0.76	0.69	0.68	0.67	0.66	0.69	0.78	0.86	0.85	0.86	0.68	0.76
[illegible]	0.42	0.43	0.49	0.52	0.43	0.46	0.58	0.50	0.50	0.40	0.45	0.48	0.75	0.35	0.94	0.77	0.52	0.57	0.69	0.56	0.74	0.72	0.71	0.75	0.47	0.56	0.69	0.57	0.72	0.67	0.11	0.74	0.59	0.60	0.70	0.58	0.56	0.59	0.59	0.59	0.75	0.85	0.84	0.84	0.58	0.72
[illegible]	0.73	0.73	0.73	0.73	0.73	0.72	0.71	0.73	0.75	0.73	0.74	0.73	0.38	0.73	0.93	0.82	0.71	0.72	0.79	0.73	0.76	0.51	0.49	0.50	0.75	0.73	0.72	0.73	0.75	0.77	0.74	0.14	0.74	0.74	0.78	0.72	0.74	0.74	0.73	0.72	0.53	0.85	0.85	0.85	0.73	0.52
[illegible]	0.63	0.64	0.63	0.66	0.63	0.60	0.56	0.64	0.64	0.62	0.63	0.63	0.74	0.58	0.94	0.79	0.62	0.58	0.69	0.59	0.74	0.72	0.72	0.76	0.64	0.63	0.72	0.59	0.74	0.60	0.59	0.74	0.04	0.09	0.72	0.60	0.57	0.57	0.57	0.58	0.77	0.85	0.85	0.81	0.60	0.72
[illegible]	0.63	0.62	0.62	0.66	0.63	0.59	0.55	0.63	0.62	0.61	0.63	0.62	0.73	0.59	0.93	0.79	0.62	0.58	0.66	0.57	0.73	0.71	0.70	0.75	0.64	0.63	0.72	0.58	0.72	0.57	0.60	0.74	0.09	0.00	0.69	0.58	0.55	0.57	0.57	0.59	0.76	0.84	0.82	0.81	0.57	0.71
[illegible]	0.69	0.69	0.72	0.73	0.69	0.71	0.70	0.70	0.73	0.69	0.72	0.71	0.78	0.69	0.95	0.83	0.73	0.52	0.00	0.55	0.74	0.77	0.77	0.82	0.71	0.73	0.76	0.72	0.73	0.76	0.70	0.78	0.72	0.69	0.00	0.39	0.72	0.67	0.67	0.74	0.82	0.86	0.85	0.85	0.54	0.77
[illegible]	0.54	0.52	0.59	0.60	0.53	0.56	0.47	0.57	0.61	0.54	0.55	0.59	0.71	0.55	0.94	0.78	0.60	0.00	0.41	0.00	0.70	0.68	0.67	0.73	0.62	0.61	0.67	0.52	0.67	0.69	0.58	0.72	0.60	0.58	0.39	0.00	0.47	0.53	0.53	0.61	0.77	0.83	0.83	0.83	0.00	0.71
[illegible]	0.60	0.59	0.58	0.63	0.60	0.56	0.29	0.61	0.61	0.56	0.59	0.60	0.73	0.56	0.93	0.78	0.58	0.45	0.68	0.43	0.72	0.68	0.67	0.74	0.61	0.61	0.70	0.31	0.72	0.68	0.56	0.74	0.57	0.55	0.72	0.47	0.00	0.47	0.47	0.61	0.76	0.85	0.84	0.83	0.44	0.71
[illegible]	0.62	0.61	0.61	0.65	0.62	0.55	0.45	0.64	0.61	0.59	0.61	0.60	0.72	0.60	0.93	0.79	0.60	0.50	0.65	0.50	0.68	0.70	0.69	0.74	0.65	0.62	0.72	0.49	0.66	0.67	0.59	0.74	0.57	0.57	0.67	0.53	0.47	0.00	0.00	0.62	0.75	0.85	0.85	0.82	0.49	0.70
[illegible]	0.62	0.61	0.62	0.65	0.61	0.55	0.45	0.64	0.62	0.58	0.61	0.60	0.72	0.60	0.93	0.79	0.60	0.50	0.66	0.49	0.68	0.70	0.69	0.74	0.66	0.62	0.72	0.49	0.66	0.66	0.59	0.73	0.57	0.57	0.67	0.53	0.47	0.00	0.00	0.62	0.75	0.85	0.84	0.82	0.49	0.70
[illegible]	0.61	0.62	0.59	0.62	0.61	0.58	0.61	0.59	0.62	0.60	0.59	0.60	0.73	0.60	0.95	0.78	0.59	0.58	0.70	0.59	0.70	0.66	0.67	0.72	0.64	0.43	0.69	0.63	0.69	0.69	0.59	0.72	0.58	0.59	0.74	0.61	0.61	0.62	0.62	0.18	0.74	0.83	0.83	0.82	0.59	0.69
[illegible]	0.75	0.75	0.76	0.75	0.75	0.76	0.76	0.76	0.77	0.75	0.75	0.75	0.55	0.75	0.93	0.83	0.74	0.75	0.80	0.75	0.78	0.59	0.56	0.54	0.76	0.75	0.75	0.77	0.77	0.78	0.75	0.53	0.77	0.76	0.82	0.77	0.76	0.75	0.75	0.74	0.42	0.88	0.88	0.87	0.77	0.58
[illegible]	0.86	0.85	0.85	0.86	0.86	0.83	0.83	0.85	0.84	0.84	0.84	0.85	0.86	0.83	0.95	0.88	0.81	0.82	0.86	0.83	0.86	0.84	0.83	0.86	0.84	0.84	0.87	0.86	0.85	0.86	0.85	0.85	0.85	0.84	0.86	0.83	0.85	0.85	0.85	0.83	0.88	0.35	0.42	0.49	0.83	0.87
[illegible]	0.85	0.84	0.84	0.86	0.85	0.82	0.84	0.84	0.84	0.84	0.84	0.85	0.86	0.83	0.95	0.87	0.83	0.82	0.86	0.83	0.85	0.85	0.84	0.86	0.84	0.83	0.86	0.85	0.84	0.85	0.84	0.85	0.85	0.82	0.85	0.83	0.84	0.85	0.84	0.83	0.88	0.42	0.38	0.51	0.82	0.87
[illegible]	0.82	0.83	0.83	0.83	0.83	0.82	0.80	0.82	0.84	0.81	0.82	0.82	0.85	0.81	0.95	0.86	0.80	0.80	0.84	0.80	0.83	0.83	0.82	0.86	0.84	0.80	0.86	0.83	0.83	0.86	0.84	0.85	0.81	0.81	0.85	0.83	0.83	0.82	0.82	0.82	0.87	0.49	0.51	0.33	0.80	0.84
[illegible]	0.54	0.56	0.57	0.61	0.54	0.55	0.45	0.56	0.60	0.54	0.55	0.58	0.70	0.55	0.95	0.77	0.57	0.00	0.54	0.00	0.67	0.66	0.65	0.73	0.59	0.59	0.68	0.49	0.64	0.68	0.58	0.73	0.60	0.57	0.54	0.00	0.44	0.49	0.49	0.59	0.77	0.83	0.82	0.80	0.00	0.70
[illegible]	0.71	0.71	0.70	0.71	0.71	0.71	0.69	0.71	0.71	0.71	0.71	0.71	0.54	0.70	0.94	0.82	0.70	0.70	0.75	0.70	0.74	0.54	0.54	0.58	0.72	0.73	0.73	0.72	0.72	0.76	0.72	0.52	0.72	0.71	0.77	0.71	0.71	0.70	0.70	0.69	0.58	0.87	0.86	0.84	0.70	0.30

[illegible]	0.49	0.00	0.14	0.24	0.45	0.00	0.53	0.22	0.31	0.34	0.00	0.28	0.52	0.43	0.80	0.81	0.70	0.13	0.56	0.01	0.81	0.51	0.48	0.60	0.74	0.26	0.58	0.54	0.86	0.61	0.13	0.56	0.16	0.23	0.69	0.00	0.67	0.14	0.00	0.58	0.68	0.80	0.83	0.85	0.00	0.82
[illegible]	0.39	0.24	0.00	0.43	0.47	0.03	0.11	0.53	0.06	0.25	0.44	0.22	0.65	0.20	0.66	0.84	0.18	0.00	0.56	0.00	0.56	0.61	0.21	0.53	0.47	0.39	0.52	0.28	0.57	0.48	0.21	0.59	0.20	0.13	0.00	0.00	0.03	0.71	0.60	0.50	0.63	0.65	0.64	0.69	0.29	0.77
[illegible]	0.13	0.00	0.46	0.32	0.26	0.31	0.48	0.47	0.37	0.20	0.26	0.00	0.38	0.36	0.85	0.80	0.09	0.00	0.37	0.00	0.61	0.63	0.51	0.66	0.47	0.18	0.21	0.00	0.76	0.19	0.44	0.58	0.55	0.00	0.06	0.13	0.00	0.13	0.13	0.28	0.66	0.65	0.64	0.87	0.00	0.39
[illegible]	0.46	0.19	0.17	0.10	0.45	0.40	0.18	0.05	0.00	0.25	0.45	0.30	0.62	0.30	0.85	0.69	0.27	0.00	0.78	0.16	0.63	0.57	0.58	0.73	0.40	0.24	0.28	0.09	0.36	0.61	0.28	0.72	0.50	0.61	0.38	0.43	0.27	0.00	0.00	0.17	0.63	0.79	0.75	0.86	0.00	0.67
[illegible]	0.00	0.00	0.00	0.15	0.35	0.06	0.26	0.27	0.48	0.00	0.05	0.35	0.57	0.20	0.53	0.83	0.00	0.00	0.01	0.00	0.56	0.41	0.58	0.83	0.02	0.47	0.22	0.27	0.71	0.42	0.30	0.62	0.45	0.24	0.50	0.00	0.01	0.25	0.00	0.21	0.69	0.56	0.64	0.86	0.00	0.53
[illegible]	0.51	0.28	0.50	0.00	0.49	0.20	0.47	0.24	0.43	0.52	0.11	0.20	0.67	0.00	0.77	0.84	0.52	0.12	0.48	0.00	0.68	0.51	0.29	0.71	0.55	0.27	0.35	0.55	0.70	0.30	0.09	0.65	0.15	0.25	0.56	0.31	0.61	0.21	0.37	0.37	0.56	0.77	0.77	0.89	0.21	0.62
[illegible]	0.65	0.47	0.56	0.17	0.31	0.57	0.25	0.31	0.53	0.17	0.20	0.48	0.47	0.51	0.79	0.68	0.68	0.47	0.71	0.62	0.85	0.45	0.68	0.77	0.63	0.09	0.49	0.15	0.77	0.14	0.31	0.57	0.18	0.00	0.88	0.56	0.29	0.47	0.48	0.43	0.78	0.83	0.72	0.69	0.51	0.74
[illegible]	0.53	0.51	0.34	0.06	0.41	0.14	0.29	0.32	0.18	0.00	0.16	0.49	0.62	0.15	0.84	0.81	0.20	0.00	0.70	0.00	0.21	0.53	0.66	0.62	0.49	0.24	0.27	0.56	0.40	0.43	0.29	0.60	0.50	0.09	0.52	0.00	0.30	0.54	0.54	0.23	0.84	0.51	0.62	0.42	0.34	0.70
[illegible]	0.33	0.29	0.18	0.18	0.44	0.47	0.30	0.40	0.53	0.48	0.03	0.14	0.67	0.40	0.90	0.82	0.22	0.02	0.48	0.00	0.72	0.56	0.54	0.46	0.39	0.24	0.46	0.49	0.73	0.47	0.36	0.36	0.42	0.21	0.38	0.00	0.00	0.00	0.00	0.30	0.75	0.62	0.63	0.80	0.00	0.69
[illegible]	0.42	0.57	0.49	0.36	0.42	0.62	0.52	0.57	0.62	0.68	0.56	0.49	0.23	0.71	0.92	0.47	0.67	0.54	0.44	0.43	0.67	0.08	0.30	0.62	0.77	0.68	0.66	0.72	0.45	0.62	0.02	0.41	0.49	0.62	0.80	0.67	0.50	0.54	0.53	0.69	0.58	0.73	0.74	0.75	0.40	0.21
[illegible]	0.39	0.37	0.15	0.35	0.07	0.34	0.27	0.44	0.26	0.11	0.00	0.29	0.69	0.21	0.81	0.80	0.55	0.10	0.33	0.20	0.78	0.48	0.63	0.57	0.05	0.74	0.48	0.42	0.55	0.67	0.22	0.43	0.31	0.55	0.73	0.48	0.07	0.68	0.09	0.00	0.86	0.80	0.36	0.81	0.34	0.63
[illegible]	0.71	0.67	0.68	0.64	0.72	0.39	0.74	0.62	0.46	0.74	0.79	0.52	0.16	0.62	0.77	0.53	0.78	0.65	0.66	0.27	0.83	0.43	0.35	0.66	0.18	0.63	0.60	0.37	0.83	0.58	0.10	0.59	0.37	0.72	0.80	0.59	0.69	0.43	0.76	0.71	0.65	0.59	0.79	0.80	0.69	0.32
[illegible]	0.31	0.05	0.54	0.73	0.18	0.61	0.28	0.50	0.31	0.25	0.27	0.73	0.05	0.11	0.85	0.40	0.60	0.31	0.55	0.38	0.21	0.30	0.40	0.63	0.50	0.79	0.66	0.51	0.14	0.45	0.38	0.56	0.01	0.18	0.69	0.40	0.42	0.66	0.66	0.55	0.48	0.78	0.81	0.76	0.67	0.43
[illegible]	0.69	0.31	0.13	0.60	0.28	0.51	0.52	0.81	0.15	0.52	0.28	0.08	0.40	0.54	0.57	0.79	0.63	0.00	0.58	0.00	0.40	0.74	0.70	0.53	0.56	0.52	0.45	0.61	0.48	0.49	0.67	0.72	0.72	0.45	0.43	0.00	0.22	0.35	0.02	0.51	0.65	0.36	0.69	0.73	0.00	0.59
[illegible]	0.51	0.01	0.23	0.57	0.39	0.14	0.23	0.67	0.16	0.35	0.34	0.36	0.67	0.26	0.89	0.80	0.42	0.51	0.33	0.25	0.54	0.67	0.61	0.51	0.20	0.50	0.34	0.58	0.73	0.68	0.27	0.82	0.36	0.42	0.64	0.28	0.31	0.31	0.31	0.23	0.46	0.81	0.83	0.89	0.35	0.63
[illegible]	0.09	0.65	0.00	0.08	0.55	0.00	0.55	0.23	0.29	0.03	0.00	0.14	0.39	0.36	0.82	0.75	0.34	0.25	0.59	0.07	0.74	0.58	0.52	0.73	0.35	0.53	0.47	0.07	0.57	0.38	0.06	0.72	0.26	0.24	0.67	0.21	0.00	0.25	0.33	0.24	0.58	0.76	0.66	0.40	0.00	0.38
[illegible]	0.56	0.08	0.75	0.73	0.56	0.65	0.40	0.46	0.59	0.61	0.54	0.70	0.01	0.50	0.91	0.70	0.68	0.67	0.60	0.49	0.84	0.04	0.38	0.19	0.36	0.62	0.83	0.60	0.88	0.45	0.30	0.61	0.00	0.54	0.82	0.74	0.37	0.64	0.63	0.72	0.31	0.89	0.81	0.75	0.73	0.49
[illegible]	0.82	0.57	0.71	0.70	0.83	0.60	0.56	0.57	0.53	0.15	0.66	0.72	0.18	0.36	0.92	0.72	0.74	0.56	0.62	0.56	0.80	0.13	0.43	0.46	0.85	0.63	0.59	0.52	0.74	0.61	0.05	0.53	0.62	0.74	0.63	0.53	0.53	0.78	0.65	0.79	0.22	0.78	0.68	0.71	0.52	0.41
[illegible]	0.00	0.16	0.13	0.58	0.08	0.20	0.42	0.00	0.53	0.10	0.17	0.32	0.58	0.16	0.78	0.72	0.34	0.00	0.44	0.32	0.66	0.70	0.35	0.76	0.09	0.46	0.47	0.52	0.72	0.31	0.66	0.57	0.11	0.00	0.60	0.34	0.52	0.34	0.29	0.49	0.64	0.79	0.86	0.79	0.35	0.56
[illegible]	0.16	0.00	0.41	0.37	0.15	0.41	0.00	0.00	0.57	0.00	0.39	0.13	0.64	0.11	0.88	0.60	0.18	0.00	0.44	0.00	0.65	0.37	0.63	0.86	0.04	0.57	0.58	0.34	0.61	0.56	0.14	0.65	0.32	0.00	0.67	0.00	0.45	0.44	0.56	0.38	0.55	0.42	0.68	0.81	0.41	0.74
[illegible]	0.13	0.41	0.10	0.36	0.00	0.05	0.19	0.48	0.58	0.61	0.37	0.39	0.44	0.37	0.79	0.61	0.14	0.00	0.69	0.18	0.64	0.69	0.64	0.62	0.00	0.48	0.37	0.28	0.53	0.71	0.66	0.69	0.27	0.00	0.52	0.00	0.00	0.17	0.18	0.40	0.56	0.73	0.83	0.69	0.41	0.57
[illegible]	0.58	0.37	0.36	0.17	0.37	0.36	0.33	0.32	0.43	0.34	0.47	0.54	0.72	0.39	0.78	0.66	0.40	0.44	0.48	0.41	0.62	0.51	0.70	0.74	0.14	0.40	0.64	0.47	0.54	0.71	0.41	0.69	0.02	0.30	0.30	0.32	0.10	0.27	0.82	0.34	0.78	0.71	0.63	0.75	0.18	0.72
[illegible]	0.08	0.15	0.00	0.37	0.00	0.42	0.53	0.06	0.64	0.11	0.59	0.32	0.63	0.17	0.79	0.64	0.36	0.55	0.57	0.48	0.66	0.58	0.56	0.82	0.38	0.56	0.29	0.51	0.72	0.12	0.53	0.71	0.42	0.30	0.57	0.00	0.44	0.74	0.48	0.49	0.46	0.65	0.84	0.55	0.47	0.34
[illegible]	0.20	0.41	0.05	0.36	0.42	0.20	0.05	0.58	0.52	0.48	0.68	0.65	0.68	0.45	0.81	0.71	0.55	0.19	0.44	0.15	0.57	0.62	0.64	0.72	0.38	0.13	0.29	0.10	0.53	0.51	0.36	0.66	0.58	0.06	0.44	0.39	0.28	0.56	0.32	0.26	0.72	0.90	0.86	0.76	0.51	0.46
[illegible]	0.42	0.00	0.19	0.33	0.53	0.05	0.03	0.72	0.42	0.15	0.09	0.05	0.40	0.00	0.73	0.67	0.51	0.34	0.66	0.00	0.69	0.34	0.32	0.69	0.00	0.33	0.29	0.65	0.81	0.13	0.00	0.66	0.06	0.12	0.66	0.00	0.45	0.22	0.22	0.15	0.66	0.68	0.81	0.62	0.35	0.63
[illegible]	0.00	0.00	0.48	0.32	0.06	0.58	0.72	0.02	0.51	0.27	0.00	0.46	0.75	0.33	0.73	0.66	0.46	0.24	0.67	0.12	0.56	0.74	0.55	0.71	0.16	0.68	0.07	0.40	0.79	0.25	0.18	0.57	0.34	0.55	0.54	0.26	0.32	0.38	0.50	0.58	0.14	0.63	0.81	0.66	0.07	0.76
[illegible]	0.53	0.57	0.58	0.43	0.64	0.52	0.42	0.51	0.24	0.50	0.77	0.48	0.89	0.32	0.84	0.65	0.56	0.51	0.61	0.43	0.81	0.81	0.77	0.40	0.00	0.40	0.52	0.28	0.67	0.78	0.67	0.71	0.44	0.06	0.62	0.25	0.17	0.52	0.41	0.33	0.69	0.78	0.71	0.61	0.34	0.52
[illegible]	0.10	0.00	0.61	0.34	0.11	0.48	0.15	0.27	0.50	0.02	0.32	0.49	0.58	0.12	0.86	0.78	0.29	0.30	0.60	0.09	0.72	0.59	0.74	0.79	0.00	0.39	0.46	0.30	0.85	0.22	0.37	0.69	0.70	0.36	0.45	0.04	0.06	0.00	0.00	0.47	0.73	0.78	0.83	0.84	0.00	0.26
[illegible]	0.17	0.39	0.37	0.47	0.59	0.68	0.09	0.00	0.77	0.32	0.15	0.51	0.45	0.43	0.90	0.63	0.50	0.07	0.39	0.24	0.73	0.81	0.70	0.64	0.00	0.46	0.33	0.09	0.84	0.70	0.50	0.74	0.36	0.39	0.48	0.29	0.26	0.73	0.73	0.67	0.64	0.69	0.85	0.54	0.36	0.76
[illegible]	0.32	0.13	0.39	0.54	0.32	0.65	0.05	0.46	0.48	0.49	0.51	0.16	0.39	0.48	0.91	0.74	0.14	0.14	0.45	0.00	0.50	0.66	0.71	0.61	0.20	0.31	0.35	0.37	0.62	0.44	0.58	0.53	0.23	0.11	0.47	0.23	0.37	0.44	0.14	0.43	0.66	0.59	0.77	0.81	0.18	0.42
[illegible]	0.58	0.64	0.44	0.72	0.63	0.68	0.40	0.75	0.89	0.58	0.45	0.39	0.09	0.87	0.87	0.39	0.67	0.60	0.58	0.60	0.75	0.03	0.09	0.30	0.40	0.70	0.53	0.63	0.76	0.59	0.29	0.57	0.52	0.61	0.58	0.47	0.39	0.57	0.57	0.42	0.35	0.72	0.91	0.45	0.59	0.01
[illegible]	0.16	0.11	0.37	0.39	0.17	0.45	0.00	0.33	0.32	0.12	0.43	0.48	0.87	0.05	0.86	0.72	0.46	0.05	0.66	0.18	0.61	0.69	0.62	0.15	0.42	0.34	0.39	0.23	0.86	0.60	0.43	0.70	0.00	0.45	0.62	0.13	0.00	0.44	0.43	0.68	0.68	0.76	0.87	0.79	0.32	0.00
[illegible]	0.78	0.88	0.79	0.78	0.79	0.81	0.73	0.73	0.84	0.86	0.90	0.91	0.87	0.86	0.76	0.69	0.80	0.85	0.78	0.83	0.83	0.91	0.93	0.84	0.88	0.77	0.83	0.81	0.91	0.80	0.89	0.94	0.75	0.64	0.80	0.67	0.83	0.79	0.79	0.86	0.90	0.71	0.73	0.78	0.81	0.93
[illegible]	0.72	0.60	0.61	0.66	0.64	0.71	0.67	0.66	0.65	0.78	0.63	0.74	0.39	0.72	0.69	0.60	0.62	0.85	0.62	0.80	0.61	0.65	0.49	0.36	0.70	0.63	0.44	0.67	0.69	0.70	0.71	0.70	0.55	0.76	0.31	0.71	0.63	0.72	0.73	0.76	0.66	0.75	0.75	0.66	0.69	0.72
[illegible]	0.34	0.18	0.14	0.40	0.36	0.55	0.51	0.46	0.56	0.29	0.50	0.14	0.67	0.46	0.80	0.62	0.17	0.19	0.50	0.00	0.77	0.79	0.68	0.58	0.03	0.34	0.51	0.46	0.78	0.52	0.66	0.54	0.14	0.00	0.46	0.10	0.03	0.00	0.01	0.45	0.76	0.68	0.73	0.87	0.15	0.61
[illegible]	0.00	0.00	0.00	0.44	0.55	0.19	0.34	0.24	0.51	0.30	0.07	0.14	0.60	0.05	0.85	0.85	0.19	0.00	0.38	0.00	0.49	0.65	0.66	0.57	0.44	0.17	0.25	0.00	0.39	0.56	0.51	0.59	0.33	0.00	0.12	0.00	0.12	0.25	0.10	0.32	0.47	0.67	0.66	0.78	0.00	0.53
[illegible]	0.44	0.44	0.69	0.48	0.57	0.44	0.66	0.67	0.61	0.60	0.39	0.45	0.58	0.66	0.78	0.62	0.50	0.38	0.00	0.55	0.27	0.75	0.47	0.56	0.61	0.67	0.70	0.50	0.62	0.27	0.28	0.62	0.65	0.46	0.21	0.42	0.56	0.20	0.25	0.64	0.59	0.63	0.74	0.68	0.71	0.56
[illegible]	0.32	0.00	0.18	0.41	0.48	0.15	0.00	0.12	0.43	0.09	0.24	0.00	0.60	0.18	0.83	0.80	0.00	0.00	0.55	0.00	0.64	0.70	0.73	0.62	0.18	0.60	0.50	0.47	0.63	0.38	0.29	0.55	0.43	0.16	0.68	0.00	0.00	0.00	0.00	0.25	0.59	0.57	0.63	0.61	0.00	0.38
[illegible]	0.66	0.65	0.64	0.62	0.66	0.57	0.69	0.56	0.81	0.72	0.73	0.50	0.75	0.61	0.83	0.61	0.77	0.49	0.27	0.64	0.25	0.83	0.80	0.64	0.61	0.62	0.75	0.63	0.44	0.75	0.57	0.84	0.46	0.03	0.63	0.48	0.70	0.56	0.45	0.68	0.70	0.68	0.72	0.78	0.70	0.45
[illegible]	0.70	0.37	0.69	0.51	0.58	0.62	0.34	0.74	0.81	0.59	0.81	0.66	0.03	0.69	0.91	0.65	0.79	0.65	0.75	0.70	0.83	0.29	0.50	0.53	0.63	0.78	0.61	0.76	0.68	0.67	0.41	0.67	0.36	0.73	0.64	0.71	0.52	0.57	0.50	0.75	0.22	0.85	0.75	0.79	0.69	0.36
[illegible]	0.35	0.63	0.64	0.70	0.56	0.64	0.32	0.55	0.77	0.74	0.70	0.71	0.09	0.62	0.93	0.49	0.68	0.66	0.47	0.73	0.80	0.50	0.27	0.49	0.44	0.62	0.39	0.48	0.81	0.46	0.49	0.67	0.49	0.51	0.61	0.59	0.26	0.71	0.65	0.74	0.12	0.51	0.78	0.79	0.61	0.56
[illegible]	0.76	0.86	0.62	0.74	0.82	0.72	0.69	0.71	0.40	0.79	0.64	0.61	0.30	0.15	0.84	0.36	0.58	0.57	0.56	0.62	0.64	0.53	0.49	0.40	0.79	0.64	0.69	0.75	0.50	0.52	0.65	0.61	0.63	0.39	0.75	0.70	0.68	0.70	0.64	0.62	0.23	0.80	0.60	0.67	0.82	0.47
[illegible]	0.09	0.04	0.00	0.14	0.38	0.38	0.00	0.16	0.00	0.00	0.00	0.20	0.40	0.42	0.88	0.70	0.03	0.44	0.61	0.18	0.61	0.63	0.44	0.79	0.16	0.38	0.09	0.34	0.70	0.41	0.32	0.82	0.39	0.43	0.77	0.16	0.52	0.09	0.59	0.30	0.77	0.88	0.68	0.78	0.21	0.61
[illegible]	0.46	0.57	0.48	0.40	0.56	0.13	0.33	0.68	0.40	0.39	0.46	0.31	0.70	0.34	0.77	0.63	0.34	0.17	0.67	0.60	0.62	0.78	0.62	0.64	0.38	0.18	0.13	0.63	0.66	0.54	0.56	0.52	0.52	0.36	0.50	0.49	0.19	0.06	0.33	0.26	0.90	0.83	0.84	0.71	0.45	0.63
[illegible]	0.47	0.58	0.37	0.64	0.29	0.29	0.29	0.07	0.52	0.46	0.33	0.35	0.53	0.39	0.83	0.44	0.51	0.25	0.70	0.50	0.75	0.61	0.39	0.69	0.09	0.13	0.40	0.43	0.82	0.40	0.24	0.78	0.39	0.26	0.28	0.26	0.44	0.61	0.66	0.00	0.60	0.68	0.86	0.88	0.28	0.35
[illegible]	0.52	0.34	0.28	0.47	0.51	0.10	0.65	0.40	0.28	0.30	0.09	0.37	0.63	0.23	0.81	0.67	0.46	0.00	0.50	0.47	0.63	0.76	0.48	0.75	0.34	0.63	0.43	0.09	0.77	0.68	0.24	0.81	0.25	0.29	0.71	0.35	0.66	0.14	0.31	0.32	0.69	0.54	0.61	0.75	0.25	0.52
[illegible]	0.72	0.61	0.53	0.54	0.72	0.53	0.81	0.79	0.67	0.85	0.84	0.62	0.76	0.86	0.91	0.69	0.78	0.39	0.62	0.63	0.44	0.68	0.81	0.50	0.70	0.66	0.82	0.77	0.22	0.53	0.56	0.71	0.53	0.18	0.58	0.73	0.77	0.59	0.49	0.68	0.50	0.66	0.70	0.62	0.53	0.52
[illegible]	0.31	0.56	0.71	0.71	0.12	0.51	0.13	0.25	0.78	0.22	0.70	0.44	0.59	0.60	0.80	0.70	0.52	0.56	0.27	0.38	0.75	0.67	0.46	0.52	0.41	0.54	0.40	0.68	0.53	0.26	0.54	0.69	0.31	0.44	0.47	0.57	0.35	0.49	0.66	0.43	0.65	0.76	0.77	0.69	0.46	0.48
[illegible]	0.66	0.14	0.66	0.41	0.53	0.36	0.00	0.18	0.67	0.37	0.50	0.58	0.29	0.43	0.89	0.71	0.66	0.51	0.28	0.29	0.57	0.41	0.49	0.65	0.32	0.56	0.24	0.24	0.56	0.54	0.10	0.61	0.05	0.27	0.48	0.13	0.00	0.60	0.60	0.12	0.78	0.75	0.75	0.78	0.27	0.48
[illegible]	0.57	0.65	0.69	0.69	0.71	0.66	0.66	0.57	0.71	0.69	0.74	0.53	0.57	0.70	0.94	0.70	0.54	0.59	0.62	0.55	0.84	0.67	0.67	0.61	0.82	0.52	0.78	0.81	0.71	0.69	0.61	0.13	0.55	0.61	0.63	0.57	0.58	0.69	0.72	0.80	0.26	0.75	0.81	0.67	0.67	0.68
[illegible]	0.11	0.32	0.27	0.02	0.42	0.58	0.06	0.34	0.44	0.70	0.36	0.23	0.52	0.00	0.75	0.55	0.14	0.33	0.65	0.43	0.46	0.36	0.49	0.63	0.39	0.52	0.39	0.25	0.53	0.31	0.05	0.55	0.03	0.27	0.72	0.20	0.25	0.31	0.10	0.40	0.74	0.51	0.80	0.79	0.42	0.23
[illegible]	0.00	0.00	0.00	0.30	0.30	0.06	0.12	0.55	0.06	0.36	0.39	0.11	0.61	0.45	0.64	0.76	0.00	0.00	0.46	0.16	0.03	0.73	0.51	0.39	0.43	0.36	0.26	0.29	0.18	0.44	0.27	0.61	0.27	0.00	0.41	0.00	0.07	0.00	0.00	0.18	0.75	0.64	0.64	0.83	0.60	0.58
[illegible]	0.60	0.67	0.52	0.30	0.57	0.44	0.66	0.54	0.62	0.45	0.48	0.47	0.58	0.62	0.80	0.31	0.46	0.12	0.21	0.68	0.63	0.64	0.61	0.75	0.77	0.50	0.28	0.71	0.58	0.47	0.48	0.63	0.72	0.41	0.00	0.42	0.60	0.52	0.74	0.83	0.66	0.85	0.90	0.69	0.76	0.57
[illegible]	0.34	0.00	0.00	0.32	0.00	0.39	0.00	0.26	0.25	0.04	0.29	0.23	0.47	0.13	0.67	0.71	0.10	0.00	0.42	0.00	0.48	0.71	0.59	0.70	0.16	0.49	0.26	0.35	0.73	0.57	0.13	0.57	0.20	0.00	0.42	0.00	0.03	0.30	0.41	0.00	0.61	0.80	0.70	0.81	0.05	0.53
[illegible]	0.52	0.45	0.00	0.10	0.44	0.28	0.45	0.32	0.17	0.06	0.26	0.37	0.39	0.00	0.83	0.63	0.03	0.12	0.56	0.00	0.70	0.52	0.26	0.68	0.52	0.19	0.44	0.66	0.77	0.35	0.00	0.58	0.25	0.07	0.60	0.03	0.00	0.46	0.21	0.52	0.52	0.82	0.82	0.79	0.29	0.42
[illegible]	0.34	0.44	0.17	0.27	0.74	0.56	0.22	0.38	0.52	0.00	0.73	0.44	0.57	0.44	0.79	0.72	0.00	0.25	0.20	0.00	0.56	0.57	0.71	0.70	0.09	0.06	0.61	0.14	0.59	0.49	0.60	0.69	0.31	0.00	0.52	0.30	0.46	0.00	0.00	0.31	0.64	0.83	0.51	0.83	0.00	0.41
[illegible]	0.29	0.56	0.18	0.82	0.48	0.32	0.22	0.50	0.41	0.00	0.73	0.14	0.57	0.43	0.79	0.73	0.01	0.10	0.25	0.00	0.45	0.50	0.65	0.64	0.59	0.33	0.66	0.31	0.49	0.66	0.60	0.72	0.10	0.00	0.74	0.41	0.21	0.00	0.00	0.31	0.81	0.78	0.63	0.46	0.00	0.41
[illegible]	0.49	0.38	0.40	0.34	0.49	0.26	0.15	0.58	0.33	0.47	0.67	0.43	0.42	0.68	0.86	0.76	0.45	0.32	0.64	0.25	0.68	0.75	0.74	0.62	0.30	0.26	0.00	0.32	0.68	0.43	0.12	0.80	0.40	0.18	0.83	0.00	0.52	0.31	0.31	0.18	0.81	0.69	0.84	0.87	0.09	0.73
[illegible]	0.64	0.55	0.56	0.78	0.46	0.72	0.66	0.14	0.69	0.73	0.64	0.66	0.35	0.68	0.90	0.66	0.76	0.47	0.59	0.59	0.70	0.22	0.12	0.23	0.77	0.90	0.60	0.69	0.50	0.65	0.78	0.26	0.74	0.75	0.66	0.61	0.52	0.64	0.81	0.81	0.42	0.80	0.72	0.60	0.76	0.16
[illegible]	0.79	0.42	0.73	0.71	0.65	0.90	0.68	0.63	0.78	0.78	0.69	0.59	0.72	0.76	0.71	0.75	0.68	0.67	0.63	0.57	0.68	0.85	0.51	0.80	0.88	0.83	0.68	0.54	0.66	0.76	0.75	0.75	0.51	0.64	0.85	0.80	0.82	0.83	0.78	0.69	0.80	0.35	0.62	0.64	0.62	0.67
[illegible]	0.86	0.68	0.83	0.63	0.84	0.86	0.81	0.81	0.71	0.83	0.85	0.77	0.91	0.87	0.73	0.75	0.73	0.66	0.74	0.63	0.72	0.75	0.78	0.60	0.68	0.84	0.86	0.61	0.70	0.77	0.75	0.81	0.80	0.64	0.90	0.70	0.82	0.51	0.63	0.84	0.72	0.62	0.36	0.66	0.66	0.80
[illegible]	0.79	0.81	0.69	0.75	0.55	0.76	0.62	0.66	0.61	0.84	0.54	0.81	0.45	0.79	0.78	0.66	0.87	0.78	0.68	0.61	0.78	0.79	0.79	0.67	0.78	0.71	0.88	0.75	0.62	0.69	0.78	0.67	0.79	0.83	0.69	0.81	0.79	0.83	0.46	0.87	0.60	0.64	0.66	0.32	0.82	0.76
[illegible]	0.35	0.41	0.41	0.18	0.47	0.51	0.35	0.07	0.34	0.00	0.36	0.18	0.59	0.32	0.81	0.69	0.15	0.00	0.71	0.00	0.70	0.69	0.61	0.82	0.21	0.45	0.28	0.25	0.53	0.46	0.27	0.67	0.42	0.60	0.76	0.05	0.29	0.00	0.00	0.09	0.76	0.62	0.66	0.82	0.00	0.68
[illegible]	0.56	0.74	0.57	0.72	0.34	0.46	0.63	0.76	0.52	0.26	0.76	0.42	0.01	0.00	0.93	0.72	0.61	0.53	0.56	0.38	0.45	0.36	0.56	0.47	0.61	0.63	0.35	0.52	0.52	0.48	0.48	0.68	0.23	0.58	0.57	0.53	0.42	0.41	0.41	0.73	0.16	0.67	0.80	0.76	0.68	0.28

MEAN																																																																
prAS-TS	0.13	0.47	0.35	0.42	0.45	0.34	0.27	0.36	0.42	0.69	0.53	0.69	0.70	0.55	0.47	0.42	0.64	0.72	0.57	0.41	0.46	0.50	0.56	0.38	0.45	0.50	0.51	0.51	0.42	0.50	0.65	0.54	0.89	0.80	0.63	0.39	0.65	0.35	0.74	0.64	0.62	0.70	0.69	0.49	0.66	0.45	0.75	0.65	0.44	0.67	0.45	0.47	0.70	0.36	0.47	0.41	0.36	0.60	0.73	0.82	0.83	0.82	0.34	0.74
agBAS-TS	0.47	0.00	0.05	0.00	0.00	0.34	0.59	0.00	0.00	0.60	0.50	0.69	0.64	0.00	0.55	0.32	0.73	0.71	0.48	0.43	0.38	0.53	0.50	0.37	0.33	0.53	0.42	0.44	0.51	0.45	0.67	0.42	0.85	0.80	0.44	0.00	0.44	0.00	0.64	0.65	0.52	0.66	0.56	0.53	0.61	0.43	0.63	0.61	0.45	0.67	0.45	0.42	0.24	0.00	0.30	0.56	0.52	0.56	0.72	0.77	0.76	0.76	0.10	0.72
gaCS-TS	0.35	0.05	0.00	0.00	0.03	0.36	0.43	0.08	0.03	0.62	0.57	0.69	0.67	0.18	0.44	0.36	0.73	0.64	0.41	0.37	0.53	0.52	0.46	0.48	0.49	0.54	0.53	0.43	0.46	0.39	0.59	0.49	0.91	0.78	0.42	0.00	0.45	0.00	0.66	0.67	0.61	0.71	0.57	0.45	0.52	0.35	0.69	0.52	0.53	0.67	0.58	0.37	0.36	0.04	0.32	0.38	0.38	0.48	0.73	0.77	0.76	0.82	0.00	0.60
ntEAS-TS	0.42	0.00	0.00	0.00	0.15	0.32	0.48	0.00	0.00	0.68	0.40	0.70	0.61	0.19	0.49	0.33	0.69	0.67	0.51	0.43	0.43	0.44	0.51	0.50	0.36	0.40	0.40	0.44	0.52	0.49	0.67	0.47	0.91	0.74	0.47	0.00	0.60	0.05	0.65	0.63	0.62	0.73	0.54	0.48	0.55	0.34	0.55	0.66	0.48	0.71	0.55	0.58	0.47	0.14	0.39	0.32	0.32	0.45	0.72	0.81	0.80	0.82	0.00	0.69
agADS-TS	0.45	0.00	0.03	0.15	0.00	0.51	0.53	0.00	0.10	0.66	0.49	0.65	0.59	0.00	0.47	0.42	0.68	0.60	0.36	0.36	0.37	0.45	0.47	0.39	0.39	0.45	0.56	0.36	0.38	0.50	0.64	0.42	0.80	0.79	0.37	0.00	0.26	0.00	0.64	0.58	0.64	0.77	0.40	0.55	0.52	0.41	0.68	0.58	0.48	0.68	0.53	0.46	0.42	0.00	0.30	0.41	0.33	0.47	0.73	0.73	0.76	0.82	0.00	0.64
atACS-TS	0.34	0.34	0.36	0.32	0.51	0.10	0.35	0.38	0.35	0.65	0.54	0.65	0.61	0.62	0.48	0.37	0.66	0.64	0.57	0.49	0.57	0.41	0.56	0.45	0.41	0.50	0.55	0.56	0.45	0.47	0.70	0.39	0.89	0.80	0.57	0.38	0.62	0.33	0.70	0.62	0.55	0.73	0.61	0.50	0.57	0.44	0.69	0.54	0.42	0.70	0.43	0.47	0.65	0.45	0.43	0.42	0.47	0.53	0.69	0.81	0.81	0.83	0.39	0.67
atAD-TS	0.27	0.59	0.43	0.48	0.53	0.35	0.13	0.54	0.46	0.71	0.59	0.63	0.70	0.54	0.45	0.32	0.66	0.66	0.62	0.57	0.59	0.47	0.51	0.57	0.35	0.53	0.59	0.45	0.49	0.57	0.63	0.56	0.89	0.75	0.63	0.51	0.69	0.55	0.75	0.61	0.68	0.76	0.64	0.44	0.62	0.32	0.71	0.50	0.50	0.67	0.46	0.40	0.76	0.54	0.34	0.52	0.52	0.55	0.77	0.83	0.79	0.77	0.51	0.71
soBPPS-TS	0.36	0.00	0.08	0.00	0.00	0.38	0.54	0.00	0.00	0.70	0.60	0.64	0.62	0.15	0.43	0.31	0.68	0.64	0.54	0.53	0.50	0.44	0.50	0.43	0.42	0.49	0.47	0.36	0.43	0.55	0.67	0.44	0.91	0.78	0.45	0.00	0.57	0.00	0.53	0.63	0.67	0.70	0.58	0.47	0.52	0.53	0.58	0.60	0.48	0.68	0.57	0.41	0.52	0.00	0.40	0.52	0.52	0.46	0.79	0.72	0.74	0.67	0.11	0.71
sfCS-TS	0.42	0.00	0.03	0.00	0.10	0.35	0.46	0.00	0.00	0.69	0.45	0.60	0.52	0.15	0.48	0.44	0.71	0.59	0.48	0.46	0.44	0.47	0.51	0.54	0.43	0.51	0.58	0.52	0.38	0.43	0.69	0.51	0.93	0.79	0.46	0.01	0.50	0.00	0.70	0.63	0.61	0.64	0.54	0.47	0.59	0.53	0.69	0.60	0.50	0.59	0.54	0.46	0.48	0.00	0.32	0.35	0.36	0.49	0.76	0.75	0.75	0.80	0.00	0.70
acDAF12-NR	0.69	0.60	0.62	0.68	0.66	0.65	0.71	0.70	0.69	0.28	0.67	0.38	0.49	0.72	0.74	0.62	0.54	0.54	0.62	0.66	0.64	0.60	0.62	0.69	0.63	0.67	0.68	0.70	0.67	0.63	0.49	0.71	0.93	0.70	0.69	0.65	0.65	0.62	0.71	0.42	0.50	0.65	0.75	0.69	0.71	0.71	0.63	0.70	0.48	0.55	0.64	0.68	0.78	0.70	0.63	0.65	0.65	0.69	0.64	0.80	0.81	0.80	0.60	0.43
saDHSQS-TS	0.53	0.50	0.57	0.40	0.49	0.54	0.59	0.60	0.45	0.67	0.16	0.60	0.62	0.66	0.63	0.48	0.59	0.52	0.55	0.55	0.46	0.55	0.44	0.53	0.47	0.58	0.51	0.44	0.44	0.51	0.73	0.48	0.90	0.78	0.61	0.44	0.57	0.46	0.74	0.63	0.69	0.70	0.46	0.66	0.64	0.54	0.65	0.49	0.48	0.64	0.42	0.49	0.72	0.56	0.41	0.61	0.42	0.40	0.79	0.83	0.69	0.82	0.52	0.69
hvECDR-NR	0.69	0.69	0.69	0.70	0.65	0.65	0.63	0.64	0.60	0.38	0.60	0.30	0.41	0.66	0.74	0.57	0.48	0.47	0.70	0.68	0.68	0.67	0.70	0.59	0.70	0.66	0.62	0.70	0.72	0.63	0.40	0.67	0.88	0.71	0.72	0.66	0.71	0.54	0.75	0.50	0.47	0.61	0.54	0.67	0.68	0.59	0.75	0.68	0.50	0.53	0.60	0.71	0.77	0.65	0.70	0.60	0.71	0.69	0.61	0.76	0.83	0.82	0.68	0.41
btECDR-NR	0.70	0.64	0.67	0.61	0.59	0.61	0.70	0.62	0.52	0.49	0.62	0.41	0.30	0.72	0.65	0.61	0.45	0.51	0.55	0.47	0.63	0.69	0.51	0.67	0.55	0.62	0.57	0.54	0.54	0.70	0.36	0.50	0.91	0.66	0.66	0.55	0.68	0.58	0.55	0.45	0.48	0.59	0.64	0.72	0.69	0.64	0.52	0.65	0.60	0.51	0.48	0.54	0.73	0.58	0.61	0.68	0.68	0.64	0.55	0.83	0.84	0.81	0.67	0.45
atECPPS-TS	0.55	0.00	0.18	0.19	0.00	0.62	0.54	0.15	0.15	0.72	0.66	0.66	0.72	0.00	0.50	0.55	0.69	0.73	0.63	0.49	0.45	0.62	0.48	0.59	0.57	0.66	0.48	0.55	0.51	0.45	0.60	0.58	0.82	0.79	0.63	0.08	0.45	0.07	0.59	0.71	0.69	0.68	0.61	0.59	0.61	0.60	0.60	0.62	0.64	0.72	0.66	0.55	0.40	0.00	0.47	0.51	0.40	0.59	0.72	0.67	0.78	0.79	0.11	0.66
bjEKS-TS	0.47	0.55	0.44	0.49	0.47	0.48	0.45	0.43	0.48	0.74	0.63	0.74	0.65	0.50	0.20	0.46	0.67	0.66	0.58	0.41	0.49	0.61	0.53	0.44	0.42	0.64	0.48	0.50	0.49	0.54	0.71	0.47	0.92	0.79	0.54	0.49	0.57	0.41	0.66	0.69	0.66	0.67	0.46	0.59	0.59	0.56	0.72	0.70	0.49	0.76	0.55	0.57	0.68	0.44	0.44	0.50	0.50	0.50	0.67	0.82	0.83	0.84	0.45	0.69
scEIZS-TS	0.42	0.32	0.36	0.33	0.42	0.37	0.32	0.31	0.44	0.62	0.48	0.57	0.61	0.55	0.46	0.00	0.53	0.52	0.40	0.60	0.38	0.43	0.56	0.36	0.33	0.45	0.50	0.37	0.36	0.45	0.60	0.46	0.90	0.77	0.51	0.36	0.63	0.30	0.72	0.63	0.61	0.73	0.51	0.57	0.61	0.23	0.65	0.58	0.38	0.71	0.43	0.44	0.67	0.37	0.17	0.39	0.42	0.48	0.69	0.80	0.77	0.67	0.28	0.59
cgESR-NR	0.64	0.73	0.73	0.69	0.68	0.66	0.66	0.68	0.71	0.54	0.59	0.48	0.45	0.69	0.67	0.53	0.33	0.47	0.66	0.50	0.72	0.71	0.66	0.69	0.60	0.63	0.68	0.67	0.65	0.70	0.34	0.64	0.93	0.77	0.69	0.69	0.71	0.63	0.77	0.37	0.47	0.43	0.60	0.68	0.76	0.68	0.78	0.64	0.58	0.52	0.48	0.66	0.79	0.72	0.60	0.69	0.69	0.71	0.48	0.86	0.83	0.80	0.71	0.51
hsESR1-NR	0.72	0.71	0.64	0.67	0.60	0.64	0.66	0.64	0.59	0.54	0.52	0.47	0.51	0.73	0.66	0.52	0.47	0.30	0.75	0.66	0.72	0.70	0.75	0.67	0.65	0.67	0.66	0.52	0.69	0.71	0.41	0.59	0.93	0.78	0.72	0.65	0.70	0.66	0.75	0.41	0.49	0.55	0.77	0.68	0.68	0.65	0.73	0.70	0.49	0.52	0.69	0.72	0.71	0.64	0.65	0.74	0.70	0.73	0.47	0.82	0.78	0.77	0.64	0.50
hsFPPS2-TS	0.57	0.48	0.41	0.51	0.36	0.57	0.62	0.54	0.48	0.62	0.55	0.70	0.55	0.63	0.58	0.40	0.66	0.75	0.00	0.10	0.31	0.50	0.04	0.39	0.53	0.26	0.50	0.21	0.30	0.39	0.66	0.27	0.87	0.75	0.42	0.35	0.58	0.47	0.69	0.70	0.57	0.74	0.32	0.55	0.60	0.58	0.70	0.57	0.50	0.67	0.45	0.41	0.65	0.47	0.57	0.52	0.50	0.57	0.72	0.82	0.84	0.81	0.48	0.66
ggFPPS-TS	0.41	0.43	0.37	0.43	0.36	0.49	0.57	0.53	0.46	0.66	0.55	0.68	0.47	0.49	0.41	0.60	0.50	0.66	0.10	0.00	0.41	0.42	0.10	0.46	0.39	0.26	0.51	0.20	0.40	0.32	0.69	0.27	0.91	0.71	0.36	0.35	0.57	0.35	0.68	0.59	0.66	0.78	0.31	0.59	0.64	0.52	0.65	0.64	0.33	0.70	0.53	0.41	0.67	0.35	0.54	0.55	0.59	0.54	0.68	0.70	0.78	0.82	0.50	0.72
ecFPPS-TS	0.46	0.38	0.53	0.43	0.37	0.57	0.59	0.50	0.44	0.64	0.46	0.68	0.63	0.45	0.49	0.38	0.72	0.72	0.31	0.41	0.11	0.33	0.27	0.34	0.47	0.46	0.45	0.40	0.43	0.32	0.62	0.36	0.88	0.72	0.33	0.37	0.68	0.44	0.68	0.69	0.66	0.70	0.32	0.55	0.58	0.50	0.62	0.69	0.55	0.71	0.51	0.42	0.65	0.38	0.39	0.47	0.47	0.53	0.69	0.80	0.83	0.78	0.52	0.65
saFPPS-TS	0.50	0.53	0.52	0.44	0.45	0.41	0.47	0.44	0.47	0.60	0.55	0.67	0.69	0.62	0.61	0.43	0.71	0.70	0.50	0.42	0.33	0.18	0.43	0.41	0.53	0.42	0.40	0.35	0.50	0.38	0.71	0.40	0.89	0.73	0.43	0.54	0.63	0.54	0.68	0.63	0.69	0.74	0.37	0.54	0.68	0.59	0.63	0.69	0.48	0.71	0.44	0.53	0.58	0.50	0.45	0.52	0.70	0.53	0.76	0.80	0.77	0.79	0.46	0.71
hsFPPS2-TS	0.56	0.50	0.46	0.51	0.47	0.56	0.51	0.50	0.51	0.62	0.44	0.70	0.51	0.48	0.53	0.56	0.66	0.75	0.04	0.10	0.27	0.43	0.01	0.47	0.56	0.27	0.54	0.21	0.44	0.39	0.68	0.27	0.88	0.72	0.43	0.53	0.62	0.52	0.69	0.66	0.64	0.77	0.42	0.58	0.54	0.57	0.70	0.50	0.47	0.72	0.56	0.51	0.64	0.36	0.54	0.65	0.56	0.57	0.66	0.77	0.83	0.73	0.52	0.59
mmGTT-TS	0.38	0.37	0.48	0.50	0.39	0.45	0.57	0.43	0.54	0.69	0.53	0.59	0.67	0.59	0.44	0.36	0.69	0.67	0.39	0.46	0.34	0.41	0.47	0.20	0.40	0.55	0.46	0.41	0.58	0.50	0.70	0.41	0.90	0.74	0.47	0.43	0.60	0.42	0.66	0.66	0.66	0.74	0.47	0.41	0.55	0.42	0.63	0.63	0.43	0.70	0.59	0.42	0.61	0.50	0.46	0.56	0.47	0.47	0.75	0.85	0.83	0.79	0.53	0.62
scGGEOS-TS	0.45	0.33	0.49	0.36	0.39	0.41	0.35	0.42	0.43	0.63	0.47	0.70	0.55	0.57	0.42	0.33	0.60	0.65	0.53	0.39	0.47	0.53	0.56	0.40	0.04	0.64	0.56	0.44	0.42	0.43	0.60	0.38	0.87	0.74	0.57	0.41	0.67	0.29	0.70	0.54	0.54	0.72	0.41	0.51	0.56	0.39	0.73	0.50	0.39	0.68	0.39	0.41	0.67	0.31	0.34	0.37	0.37	0.46	0.73	0.78	0.82	0.73	0.42	0.67
scGGPPS-TS	0.50	0.54	0.54	0.40	0.45	0.50	0.53	0.49	0.51	0.67	0.58	0.66	0.62	0.66	0.64	0.45	0.63	0.67	0.26	0.26	0.46	0.42	0.27	0.55	0.64	0.03	0.55	0.32	0.20	0.47	0.72	0.39	0.87	0.73	0.49	0.46	0.65	0.42	0.66	0.71	0.65	0.74	0.35	0.62	0.48	0.56	0.71	0.55	0.39	0.67	0.53	0.59	0.64	0.47	0.51	0.54	0.58	0.59	0.55	0.77	0.83	0.76	0.40	0.72
saGGPPS-TS	0.51	0.42	0.53	0.40	0.56	0.55	0.59	0.47	0.58	0.68	0.51	0.62	0.57	0.48	0.48	0.50	0.68	0.66	0.50	0.51	0.45	0.40	0.54	0.46	0.56	0.55	0.25	0.40	0.63	0.36	0.78	0.39	0.91	0.73	0.51	0.57	0.67	0.54	0.74	0.75	0.73	0.64	0.36	0.54	0.64	0.52	0.68	0.71	0.56	0.73	0.57	0.43	0.68	0.48	0.46	0.58	0.55	0.52	0.74	0.81	0.79	0.75	0.51	0.64
phGGPPS-TS	0.51	0.44	0.43	0.44	0.36	0.56	0.45	0.36	0.52	0.70	0.44	0.70	0.54	0.55	0.50	0.37	0.67	0.52	0.21	0.20	0.40	0.35	0.21	0.41	0.44	0.32	0.40	0.03	0.34	0.35	0.66	0.17	0.90	0.78	0.35	0.45	0.63	0.39	0.70	0.65	0.71	0.77	0.26	0.52	0.61	0.50	0.72	0.52	0.39	0.71	0.64	0.52	0.60	0.37	0.39	0.39	0.39	0.56	0.74	0.81	0.83	0.81	0.36	0.55
hsGGPPS-TS	0.42	0.51	0.46	0.52	0.38	0.45	0.49	0.43	0.38	0.67	0.44	0.72	0.54	0.51	0.49	0.36	0.65	0.69	0.30	0.40	0.43	0.50	0.44	0.58	0.42	0.20	0.63	0.34	0.16	0.49	0.63	0.39	0.93	0.73	0.50	0.39	0.57	0.45	0.72	0.72	0.68	0.72	0.29	0.54	0.56	0.44	0.75	0.70	0.46	0.73	0.54	0.55	0.63	0.46	0.48	0.65	0.65	0.61	0.72	0.78	0.84	0.72	0.49	0.72
mpGPPS-TS	0.50	0.45	0.39	0.49	0.50	0.47	0.57	0.55	0.43	0.63	0.51	0.63	0.70	0.45	0.54	0.45	0.70	0.71	0.39	0.32	0.32	0.38	0.39	0.50	0.43	0.47	0.36	0.35	0.49	0.18	0.61	0.40	0.93	0.76	0.33	0.42	0.60	0.38	0.62	0.69	0.69	0.70	0.38	0.51	0.57	0.54	0.64	0.59	0.51	0.66	0.49	0.45	0.62	0.47	0.53	0.54	0.45	0.54	0.72	0.76	0.81	0.81	0.44	0.61
hsHNF1-NR	0.65	0.67	0.59	0.67	0.64	0.70	0.63	0.67	0.69	0.49	0.73	0.40	0.36	0.60	0.71	0.59	0.34	0.41	0.66	0.69	0.62	0.71	0.68	0.70	0.60	0.72	0.78	0.66	0.63	0.61	0.11	0.76	0.91	0.67	0.69	0.66	0.69	0.66	0.74	0.37	0.36	0.46	0.61	0.71	0.66	0.68	0.75	0.70	0.58	0.41	0.66	0.69	0.70	0.62	0.61	0.67	0.67	0.62	0.48	0.80	0.87	0.71	0.66	0.37
saHPDS-TS	0.54	0.42	0.49	0.47	0.42	0.39	0.56	0.44	0.51	0.71	0.48	0.67	0.50	0.58	0.47	0.46	0.64	0.59	0.27	0.27	0.36	0.40	0.27	0.41	0.38	0.39	0.39	0.17	0.39	0.40	0.76	0.06	0.91	0.76	0.43	0.38	0.66	0.42	0.69	0.69	0.67	0.53	0.39	0.50	0.59	0.46	0.76	0.65	0.38	0.71	0.38	0.54	0.66	0.41	0.37	0.55	0.54	0.63	0.72	0.80	0.83	0.80	0.47	0.47
saHEPPS-TS	0.89	0.85	0.91	0.91	0.80	0.89	0.89	0.91	0.93	0.93	0.90	0.88	0.91	0.82	0.92	0.90	0.93	0.93	0.87	0.91	0.88	0.89	0.88	0.90	0.87	0.87	0.91	0.90	0.93	0.93	0.91	0.91	0.76	0.86	0.89	0.91	0.89	0.91	0.90	0.92	0.93	0.90	0.92	0.88	0.90	0.89	0.93	0.89	0.92	0.93	0.88	0.84	0.90	0.85	0.90	0.89	0.88	0.91	0.92	0.87	0.88	0.90	0.90	0.93
mlHXDS-TS	0.80	0.80	0.78	0.74	0.79	0.80	0.75	0.78	0.79	0.70	0.78	0.71	0.66	0.79	0.79	0.77	0.77	0.78	0.75	0.71	0.72	0.73	0.72	0.74	0.74	0.73	0.73	0.78	0.73	0.76	0.67	0.76	0.86	0.61	0.71	0.80	0.74	0.78	0.74	0.75	0.70	0.66	0.73	0.72	0.67	0.74	0.77	0.76	0.75	0.78	0.70	0.78	0.65	0.76	0.73	0.76	0.77	0.77	0.77	0.83	0.82	0.78	0.74	0.78
ssHPPS-TS	0.63	0.44	0.42	0.47	0.37	0.57	0.63	0.45	0.46	0.69	0.61	0.72	0.66	0.63	0.54	0.51	0.69	0.72	0.42	0.36	0.33	0.43	0.43	0.47	0.57	0.49	0.51	0.35	0.50	0.33	0.69	0.43	0.90	0.71	0.18	0.44	0.61	0.38	0.72	0.72	0.67	0.69	0.32	0.49	0.63	0.55	0.71	0.62	0.57	0.66	0.46	0.41	0.63	0.42	0.40	0.40	0.40	0.54	0.75	0.77	0.79	0.82	0.43	0.67
pcIPS-TS	0.39	0.00	0.00	0.00	0.00	0.38	0.51	0.00	0.01	0.65	0.44	0.66	0.55	0.08	0.49	0.36	0.69	0.65	0.35	0.35	0.37	0.54	0.53	0.43	0.41	0.46	0.57	0.45	0.39	0.42	0.66	0.38	0.91	0.80	0.44	0.00	0.44	0.00	0.61	0.65	0.65	0.68	0.54	0.44	0.52	0.33	0.56	0.63	0.55	0.67	0.50	0.38	0.38	0.00	0.34	0.41	0.36	0.49	0.65	0.77	0.76	0.79	0.00	0.64
hsLS-TS	0.65	0.44	0.45	0.60	0.26	0.62	0.69	0.57	0.50	0.65	0.57	0.71	0.68	0.45	0.57	0.63	0.71	0.70	0.58	0.57	0.68	0.63	0.62	0.60	0.67	0.65	0.67	0.63	0.57	0.60	0.69	0.66	0.89	0.74	0.61	0.44	0.00	0.52	0.57	0.74	0.64	0.72	0.67	0.69	0.72	0.62	0.68	0.58	0.54	0.72	0.67	0.58	0.07	0.41	0.63	0.49	0.51	0.67	0.72	0.77	0.81	0.78	0.58	0.68
msLS-TS	0.35	0.00	0.00	0.05	0.00	0.33	0.55	0.00	0.00	0.62	0.46	0.54	0.58	0.07	0.41	0.30	0.63	0.66	0.47	0.35	0.44	0.54	0.52	0.42	0.29	0.42	0.54	0.39	0.45	0.38	0.66	0.42	0.91	0.78	0.38	0.00	0.52	0.00	0.66	0.68	0.68	0.70	0.46	0.58	0.60	0.50	0.65	0.58	0.47	0.65	0.54	0.43	0.58	0.00	0.29	0.33	0.33	0.47	0.69	0.74	0.76	0.73	0.00	0.59
ecPGPB-TS	0.74	0.64	0.66	0.65	0.64	0.70	0.75	0.53	0.70	0.71	0.74	0.75	0.55	0.59	0.66	0.72	0.77	0.75	0.69	0.67	0.68	0.68	0.69	0.66	0.70	0.66	0.74	0.70	0.72	0.62	0.74	0.69	0.90	0.74	0.72	0.61	0.57	0.66	0.25	0.76	0.75	0.72	0.69	0.66	0.70	0.68	0.34	0.75	0.67	0.78	0.64	0.49	0.70	0.62	0.71	0.63	0.59	0.69	0.75	0.79	0.80	0.81	0.68	0.63
hsLRH1-NR	0.64	0.65	0.67	0.63	0.58	0.62	0.61	0.63	0.63	0.42	0.63	0.50	0.45	0.71	0.69	0.63	0.37	0.41	0.70	0.59	0.69	0.63	0.66	0.66	0.54	0.71	0.75	0.65	0.72	0.69	0.37	0.69	0.92	0.75	0.72	0.65	0.74	0.68	0.76	0.30	0.39	0.56	0.68	0.72	0.68	0.71	0.71	0.72	0.61	0.56	0.59	0.71	0.72	0.68	0.63	0.65	0.63	0.69	0.47	0.84	0.81	0.81	0.67	0.48
mmLRH1-NR	0.62	0.52	0.61	0.62	0.64	0.55	0.68	0.67	0.61	0.50	0.69	0.47	0.48	0.69	0.66	0.61	0.47	0.49	0.57	0.66	0.66	0.69	0.64	0.66	0.54	0.65	0.73	0.71	0.68	0.69	0.36	0.67	0.93	0.70	0.67	0.65	0.64	0.68	0.75	0.39	0.28	0.54	0.61	0.67	0.60	0.62	0.74	0.65	0.63	0.55	0.63	0.64	0.71	0.64	0.53	0.70	0.68	0.69	0.42	0.72	0.82	0.81	0.64	0.54
bfNR-NR	0.70	0.66	0.71	0.73	0.77	0.73	0.76	0.70	0.64	0.65	0.70	0.61	0.59	0.68	0.67	0.73	0.43	0.55	0.74	0.78	0.70	0.74	0.77	0.74	0.72	0.74	0.64	0.77	0.72	0.70	0.46	0.53	0.90	0.66	0.69	0.68	0.72	0.70	0.72	0.56	0.54	0.42	0.77	0.71	0.72	0.76	0.67	0.69	0.71	0.54	0.71	0.62	0.79	0.71	0.72	0.72	0.70	0.69	0.44	0.84	0.77	0.79	0.76	0.55
tmOPPS-TS	0.69	0.56	0.57	0.54	0.40	0.61	0.64	0.58	0.54	0.75	0.46	0.54	0.64	0.61	0.46	0.51	0.60	0.77	0.32	0.31	0.32	0.37	0.42	0.47	0.41	0.35	0.36	0.26	0.29	0.38	0.61	0.39	0.92	0.73	0.32	0.54	0.67	0.46	0.69	0.68	0.61	0.77	0.18	0.53	0.49	0.54	0.71	0.60	0.42	0.76	0.56	0.57	0.73	0.47	0.58	0.46	0.63	0.52	0.76	0.85	0.78	0.81	0.46	0.68
apOPT-TS	0.49	0.53	0.45	0.48	0.55	0.50	0.44	0.47	0.47	0.69	0.66	0.67	0.72	0.59	0.59	0.57	0.68	0.68	0.55	0.59	0.55	0.54	0.58	0.41	0.51	0.62	0.54	0.52	0.54	0.51	0.71	0.50	0.88	0.72	0.49	0.44	0.69	0.58	0.66	0.72	0.67	0.71	0.53	0.18	0.50	0.61	0.66	0.64	0.56	0.65	0.59	0.54	0.65	0.57	0.47	0.42	0.51	0.37	0.80	0.83	0.83	0.77	0.54	0.69
bsPGP-TS	0.66	0.61	0.52	0.55	0.52	0.57	0.62	0.52	0.59	0.71	0.64	0.68	0.69	0.61	0.59	0.61	0.76	0.68	0.60	0.64	0.58	0.68	0.54	0.55	0.56	0.48	0.64	0.61	0.56	0.57	0.66	0.59	0.90	0.67	0.63	0.52	0.72	0.60	0.70	0.68	0.60	0.72	0.49	0.50	0.40	0.61	0.72	0.63	0.54	0.74	0.61	0.57	0.60	0.53	0.61	0.68	0.69	0.46	0.70	0.80	0.86	0.85	0.54	0.60
stPS-TS	0.45	0.43	0.35	0.34	0.41	0.44	0.32	0.53	0.53	0.71	0.54	0.59	0.64	0.60	0.56	0.23	0.68	0.65	0.58	0.52	0.50	0.59	0.57	0.42	0.39	0.56	0.52	0.50	0.44	0.54	0.68	0.46	0.89	0.74	0.55	0.33	0.62	0.50	0.68	0.71	0.62	0.76	0.54	0.61	0.61	0.10	0.72	0.69	0.46	0.75	0.47	0.48	0.71	0.46	0.43	0.37	0.43	0.52	0.74	0.74	0.76	0.79	0.41	0.65
ecPGP-TS	0.75	0.63	0.69	0.55	0.68	0.69	0.71	0.58	0.69	0.63	0.65	0.75	0.52	0.60	0.72	0.65	0.78	0.73	0.70	0.65	0.62	0.63	0.70	0.63	0.73	0.71	0.68	0.72	0.75	0.64	0.75	0.76	0.93	0.77	0.71	0.56	0.68	0.65	0.34	0.71	0.74	0.67	0.71	0.66	0.72	0.72	0.23	0.67	0.65	0.73	0.65	0.53	0.67	0.68	0.72	0.63	0.60	0.68	0.67	0.78	0.78	0.75	0.60	0.65
bsPSS-TS	0.65	0.61	0.52	0.66	0.58	0.54	0.50	0.60	0.60	0.70	0.49	0.68	0.65	0.62	0.70	0.58	0.64	0.70	0.57	0.64	0.69	0.69	0.50	0.63	0.50	0.55	0.71	0.52	0.70	0.59	0.70	0.65	0.89	0.76	0.62	0.63	0.58	0.58	0.75	0.72	0.65	0.69	0.60	0.64	0.63	0.69	0.67	0.27	0.63	0.74	0.51	0.53	0.66	0.64	0.57	0.61	0.66	0.60	0.74	0.82	0.82	0.79	0.60	0.67
atPPPS-TS	0.44	0.45	0.53	0.48	0.48	0.42	0.50	0.48	0.50	0.48	0.48	0.50	0.60	0.64	0.49	0.38	0.58	0.49	0.50	0.33	0.55	0.48	0.47	0.43	0.39	0.39	0.56	0.39	0.46	0.51	0.59	0.38	0.93	0.75	0.57	0.55	0.54	0.47	0.67	0.61	0.63	0.71	0.42	0.56	0.54	0.46	0.65	0.63	0.11	0.69	0.41	0.49	0.62	0.43	0.37	0.59	0.60	0.43	0.76	0.81	0.81	0.81	0.47	0.64
hsRXRA-NR	0.67	0.67	0.67	0.71	0.68	0.70	0.67	0.68	0.59	0.55	0.64	0.53	0.51	0.72	0.76	0.71	0.52	0.52	0.67	0.70	0.71	0.71	0.72	0.70	0.68	0.67	0.73	0.71	0.73	0.66	0.41	0.71	0.93	0.78	0.66	0.67	0.72	0.65	0.78	0.56	0.55	0.54	0.76	0.65	0.74	0.75	0.73	0.74	0.69	0.14	0.67	0.69	0.72	0.66	0.67	0.71	0.72	0.75	0.44	0.81	0.84	0.78	0.69	0.57
hsSQS-TS	0.45	0.45	0.58	0.55	0.53	0.43	0.46	0.57	0.54	0.64	0.42	0.60	0.48	0.66	0.55	0.43	0.48	0.69	0.45	0.53	0.51	0.44	0.56	0.59	0.39	0.53	0.57	0.64	0.54	0.49	0.66	0.38	0.88	0.70	0.46	0.50	0.67	0.54	0.63	0.59	0.63	0.71	0.56	0.58	0.61	0.47	0.65	0.51	0.41	0.67	0.04	0.15	0.71	0.47	0.46	0.48	0.41	0.52	0.76	0.72	0.82	0.80	0.54	0.56
tcSQS-TS	0.47	0.42	0.37	0.58	0.46	0.47	0.40	0.41	0.46	0.68	0.49	0.71	0.54	0.55	0.57	0.44	0.66	0.72	0.41	0.41	0.42	0.53	0.51	0.42	0.41	0.59	0.43	0.52	0.55	0.45	0.69	0.54	0.84	0.78	0.41	0.38	0.58	0.43	0.49	0.71	0.64	0.62	0.57	0.54	0.57	0.48	0.53	0.53	0.49	0.69	0.15	0.00	0.58	0.39	0.39	0.38	0.38	0.45	0.76	0.76	0.75	0.82	0.58	0.67
aaSQHC-TS	0.70	0.24	0.36	0.47	0.42	0.65	0.76	0.52	0.48	0.78	0.72	0.77	0.73	0.40	0.68	0.67	0.79	0.71	0.65	0.67	0.65	0.58	0.64	0.61	0.67	0.64	0.68	0.60	0.63	0.62	0.70	0.66	0.90	0.65	0.63	0.38	0.07	0.58	0.70	0.72	0.71	0.79	0.73	0.65	0.60	0.71	0.67	0.66	0.62	0.72	0.71	0.58	0.00	0.40	0.67	0.62	0.69	0.76	0.76	0.85	0.86	0.79	0.60	0.70
tbTXD-TS	0.36	0.00	0.04	0.14	0.00	0.45	0.54	0.00	0.00	0.70	0.56	0.65	0.58	0.00	0.44	0.37	0.72	0.64	0.47	0.35	0.38	0.50	0.36	0.50	0.31	0.47	0.48	0.37	0.46	0.47	0.62	0.41	0.85	0.76	0.42	0.00	0.41	0.00	0.62	0.68	0.64	0.71	0.47	0.57	0.53	0.46	0.68	0.64	0.43	0.66	0.47	0.39	0.40	0.00	0.32	0.46	0.49	0.40	0.71	0.81	0.77	0.81	0.02	0.64
spTS-TS	0.47	0.30	0.32	0.39	0.30	0.43	0.34	0.40	0.32	0.63	0.41	0.70	0.61	0.47	0.44	0.17	0.60	0.65	0.57	0.54	0.39	0.45	0.54	0.46	0.34	0.51	0.46	0.39	0.48	0.53	0.61	0.37	0.90	0.73	0.40	0.34	0.63	0.29	0.71	0.63	0.53	0.72	0.58	0.47	0.61	0.43	0.72	0.57	0.37	0.67	0.46	0.39	0.67	0.32	0.00	0.47	0.38	0.58	0.68	0.83	0.83	0.81	0.39	0.61
fsTDS-TS	0.41	0.56	0.38	0.32	0.41	0.42	0.52	0.52	0.35	0.65	0.61	0.60	0.68	0.51	0.50	0.39	0.69	0.74	0.52	0.55	0.47	0.52	0.65	0.56	0.37	0.54	0.58	0.39	0.65	0.54	0.67	0.55	0.89	0.76	0.40	0.41	0.49	0.33	0.63	0.65	0.70	0.72	0.46	0.42	0.68	0.37	0.63	0.61	0.59	0.71	0.48	0.38	0.62	0.46	0.47	0.00	0.00	0.51	0.71	0.83	0.72	0.82	0.32	0.60
fsTDS-TS	0.36	0.52	0.38	0.32	0.33	0.47	0.52	0.52	0.36	0.65	0.42	0.71	0.68	0.40	0.50	0.42	0.69	0.70	0.50	0.59	0.47	0.70	0.56	0.47	0.37	0.58	0.55	0.39	0.65	0.45	0.67	0.54	0.88	0.77	0.40	0.36	0.51	0.33	0.59	0.63	0.68	0.70	0.63	0.51	0.69	0.43	0.60	0.66	0.60	0.72	0.41	0.38	0.69	0.49	0.38	0.00	0.00	0.51	0.77	0.82	0.76	0.70	0.32	0.60
afPT-TS	0.60	0.56	0.48	0.45	0.47	0.53	0.55	0.46	0.49	0.69	0.40	0.69	0.64	0.59	0.50	0.48	0.71	0.73	0.57	0.54	0.53	0.53	0.57	0.47	0.46	0.59	0.52	0.56	0.61	0.54	0.62	0.63	0.92	0.77	0.54	0.49	0.67	0.47	0.69	0.69	0.69	0.69	0.52	0.37	0.46	0.52	0.68	0.60	0.43	0.75	0.52	0.45	0.76	0.40	0.58	0.51	0.51	0.18	0.77	0.78	0.83	0.83	0.42	0.70
btUSP-NR	0.73	0.72	0.73	0.72	0.73	0.69	0.77	0.79	0.76	0.64	0.79	0.61	0.55	0.72	0.67	0.69	0.48	0.47	0.72	0.68	0.69	0.76	0.66	0.75	0.73	0.55	0.74	0.74	0.72	0.72	0.48	0.72	0.92	0.77	0.75	0.65	0.72	0.69	0.75	0.47	0.42	0.44	0.76	0.80	0.70	0.74	0.67	0.74	0.76	0.44	0.76	0.76	0.76	0.71	0.68	0.71	0.77	0.77	0.42	0.84	0.82	0.77	0.76	0.44
mlUDPPS-TS	0.82	0.77	0.77	0.81	0.73	0.81	0.83	0.72	0.75	0.80	0.83	0.76	0.83	0.67	0.82	0.80	0.86	0.82	0.82	0.70	0.80	0.80	0.77	0.85	0.78	0.77	0.81	0.81	0.78	0.76	0.80	0.80	0.87	0.83	0.77	0.77	0.77	0.74	0.79	0.84	0.72	0.84	0.85	0.83	0.80	0.74	0.78	0.82	0.81	0.81	0.72	0.76	0.85	0.81	0.83	0.83	0.82	0.78	0.84	0.35	0.49	0.54	0.75	0.79
ecUDPPS-TS	0.83	0.76	0.76	0.80	0.76	0.81	0.79	0.74	0.75	0.81	0.69	0.83	0.84	0.78	0.83	0.77	0.83	0.78	0.84	0.78	0.83	0.77	0.83	0.83	0.82	0.83	0.79	0.83	0.84	0.81	0.87	0.83	0.88	0.82	0.79	0.76	0.81	0.76	0.80	0.81	0.82	0.77	0.78	0.83	0.86	0.76	0.78	0.82	0.81	0.84	0.82	0.75	0.86	0.77	0.83	0.72	0.76	0.83	0.82	0.49	0.37	0.56	0.76	0.83
mtUDPPS-TS	0.82	0.76	0.82	0.82	0.82	0.83	0.77	0.67	0.80	0.80	0.83	0.82	0.81	0.79	0.84	0.67	0.80	0.77	0.81	0.82	0.78	0.79	0.73	0.79	0.73	0.76	0.75	0.81	0.72	0.81	0.71	0.80	0.90	0.78	0.82	0.79	0.78	0.73	0.81	0.81	0.81	0.79	0.81	0.77	0.86	0.79	0.75	0.79	0.81	0.78	0.80	0.82	0.79	0.81	0.81	0.82	0.70	0.83	0.77	0.54	0.56	0.33	0.80	0.81
hmVSS-TS	0.34	0.10	0.00	0.00	0.00	0.39	0.51	0.11	0.00	0.60	0.52	0.68	0.67	0.11	0.45	0.28	0.71	0.64	0.48	0.50	0.52	0.46	0.52	0.53	0.42	0.40	0.51	0.36	0.49	0.44	0.66	0.47	0.90	0.74	0.43	0.00	0.58	0.00	0.68	0.67	0.64	0.76	0.46	0.54	0.54	0.41	0.60	0.60	0.47	0.69	0.53	0.58	0.60	0.02	0.39	0.32	0.32	0.42	0.76	0.75	0.76	0.80	0.00	0.69
drVDR-NR	0.74	0.72	0.60	0.69	0.64	0.67	0.71	0.71	0.70	0.43	0.69	0.41	0.45	0.66	0.69	0.59	0.51	0.50	0.66	0.72	0.65	0.71	0.59	0.62	0.67	0.72	0.64	0.55	0.72	0.61	0.37	0.47	0.93	0.78	0.67	0.64	0.68	0.59	0.63	0.48	0.54	0.55	0.68	0.69	0.60	0.65	0.65	0.67	0.64	0.57	0.56	0.67	0.70	0.64	0.61	0.60	0.60	0.70	0.44	0.79	0.83	0.81	0.69	0.30
