# Supplementary material for: Evidence that nuclear receptors are related to terpene synthases
Source: J Mol Endocrinol. 2022 Feb 3;68(3):153–66. doi: 10.1530/JME-21-0156 (PMC8942334; doi:10.1530/JME-21-0156)
Supplement: supplementary Table S3 [file supplementary_table_3.pdf]

Table S3. Summary of Ligand Contacts with Nuclear Receptors and Terpene Synthases

A. Nuclear Receptor and Terpene Synthase Ligands

MOLECULE	STRUCTURE	LIGAND	METHOD	CONTACTS									KCAL/MOL
						Glu(Leu)Arg pair							
						E		L	R				
					Asp351	Gu353		Leu387	Arg394	Phe404	His524		
Human steroid receptor ESR1	1QKU	Estradiol (E2)	Crystal/Dock			Glu353		Leu387	Arg394	Phe404	His524		-10.8
	1QKU	Phosphatidylglycerol (PG)	Dock			Glu353		Leu387	Arg394				-4.8
	1QKU	Dafachronic acid (DAFA)	Dock					Leu387	Arg394				-8.4
	1QKU	Farnesyl pyrophosphate (FPP)	Dock			Glu353		Leu387	Arg394		His524		-7.3
	5W9C	4OHTamoxifen	Crystal		Asp351	Glu353			Arg394				NA
	1ERR	Raloxifene	Crystal		Asp351	Glu353		Leu387	Arg394	Phe404			NA
	1ERE	Estradiol (E2)	Crystal			Glu353		Leu387	Arg394	Phe404			
					(Gly) and Lys Asp pair								
					G		L	Y	K	D			
				Gln419	Gly421	Thr423	Leu424	Tyr516	Lys520	Asp525			
Human nuclear receptor LRH1	1YOK	Estradiol (E2)	Dock	Gln419		Thr423							-7.5
	1YOK	Phosphatidylglycerol (PG)	Dock/Crystal	Gln419	Gly421	Thr423	Leu424	Tyr516	Lys520	Asp525			-7.2
	1YOK	Dafachronic acid (DAFA)	Dock	Gln419				Tyr516	Lys520				-9.5
	1YOK	Farnesyl pyrophosphate (FPP)	Dock	Gln419	Gly421			Tyr516	Lys520				-7.1
	4DOS	Diundecylphosphoatidylcholine	Crystal		Gly421		Leu424	Tyr516	Lys520				
						Thr Arg pair							
						T			R				
						Thr562			Arg599	Thr613	Gln637		
Worm nuclear receptor DAF-12	3GYT	Estradiol (E2)	Dock										-10.4
	3GYT	Phosphatidylglycerol (PG)	Dock			Thr562					Gln637		-5.6
	3GYT	Dafachronic acid (DAFA)	Dock/Crystal			Thr562			Arg599		Gln637		-12.5
	3GYT	Farnesyl pyrophosphate (FPP)	Dock			Thr562			Arg599		Gln637		-8.5
	3GYU	Isomer of dafachronic acid/DAFA	Crystal			Thr562			Arg599	Thr613	Gln637		
				AspAsp-Arg(Lys) cluster									
				DDxxD			RR loop		K	DD			
				Asp117	Asp118	Asp121	Arg126	Arg127	Lys214	Asp257	Asp258	Lys280	
Chicken farnesyl pyrophosphosphate synthase (FPPS)	1FPS	Estradiol (E2)	Dock			Asp121	Arg126	Arg127		Asp257	Asp258		-7.2
	1FPS	Phosphatidylglycerol (PG)	Dock			Asp121	Arg126		Lys214				-5.6
	1FPS	Dafachronic acid (DAFA)	Dock					Arg127	Lys214				-8.1
	1FPS	Farnesyl pyrophosphate (FPP)	Dock	Asp117		Asp121	Arg126		Lys214	Asp257	Asp258		-6.6
	1UBY	Dimethylallyl pyrophosphate (DMAPP)	Crystal		Asp118	Asp121	Arg126		Lys214				NA
	1UBW	Geranyl diphosphate (GPP)	Crystal	Asp117		Asp121	Arg126		Lys214			Lys280	NA

				Arg coordinates the DxxD cluster									
				R			DxxxD			DD			
				Arg264	Trp273	Ser298	Asp301	Asp305	Arg441	Asp444	Asp445	Thr448	
**Plant 5-epi-aristolochene synthase (5EAS)**	5EAT	Estradiol (E2)	Dock							Asp444			-8.6
	5EAT	Phosphatidylglycerol (PG)	Dock				Asp301				Asp445		-7.7
	5EAT	Dafachronic acid (DAFA)	Dock			Ser298							-10.3
	5EAT	Farnesyl pyrophosphate (FPP)	Dock	Arg264			Asp301	Asp305		Asp444	Asp445		-8
	4RNQ	Anilogeranyl pyrophosphate	Crystal	Arg264			Asp301	Asp305	Arg441	Asp444		Thr448	NA
				The Args coordinate the DxxD cluster									
				DxxD		R loop		R		R			
				Asp84	Asp88	Arg93	Glu146	Lys170		Lys235			
**Bacterial octaprenyl pyrophosphate synthase (OPPS)**	3WJN	Estradiol (E2)	Dock					Lys170					-7.3
	3WJN	Phosphatidylglycerol (PG)	Dock			Arg93		Lys170		Lys235			-6.3
	3WJN	Dafachronic acid (DAFA)	Dock			Arg93							-8.2
	3WJN	Farnesyl pyrophosphate (FPP)	Dock			(Arg93)							-6.6
	3WJO	Isopentenyl pyrophosphate (IPP)	Crystal	Asp84				Lys170					NA
	5ZE6	BPH-981	Crystal	Asp84		(Arg93)	Glu146						NA
	5ZLF	BPJ-629	Crystal	Asp84	(Asp88)	Arg93							NA

B. Bisphosphates and Nuclear Receptors

MOLECULE	STRUCTURE	LIGAND	METHOD	CONTACTS									KCAL/MOL
						Glu(Leu)Arg pair							
						E		L	R				
					Asp351	Gu353		Leu387	Arg394	His524			
**Human steroid receptor ESR1**	1QKU	Estradiol (E2)	Crystal			Glu353		Leu387	Arg394	His524			NA
	1QKU	Farnesyl pyrophosphate (FPP)	Dock			Glu353		Leu387	Arg394	His524			-7.3
	1QKU	Zoledronic acid	Dock			Glu353		Leu387	Arg394				-4.13
													-5.1
				Asp273	Glu275	Tyr326	Asn346						
**ERR1**	2EWP	Tamoxifen analog GSK5182	Crystal	Asp273	Glu275	Tyr326	Asn346						NA
	2EWP	Zoledronic acid	Dock		Glu275	Tyr326	Asn346						-5.1
