# Supplementary figures and images for: Evidence that nuclear receptors are related to terpene synthases
Source: J Mol Endocrinol. 2022 Feb 3;68(3):153–66. doi: 10.1530/JME-21-0156 (PMC8942334; doi:10.1530/JME-21-0156)

# TREE: FATCATflexible\_Fitch-Margolaish

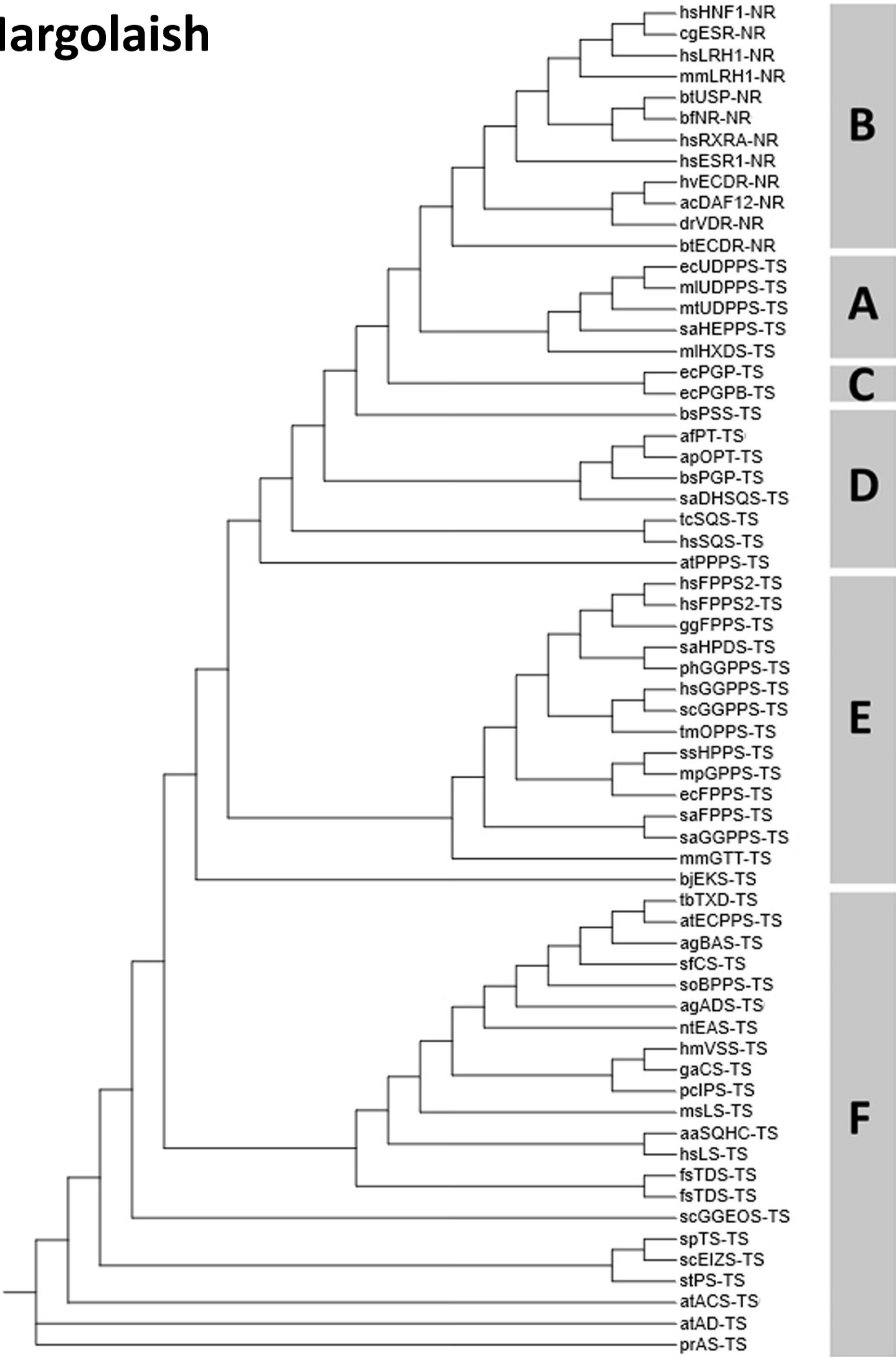

Supplement: Sup fig 1: Phylogenetic tree generated by FATCATflexible + Fitch–Margoliash. [file supplementary_figure_1.pdf]

# TREE:FATCATflexible\_

## UPGMA

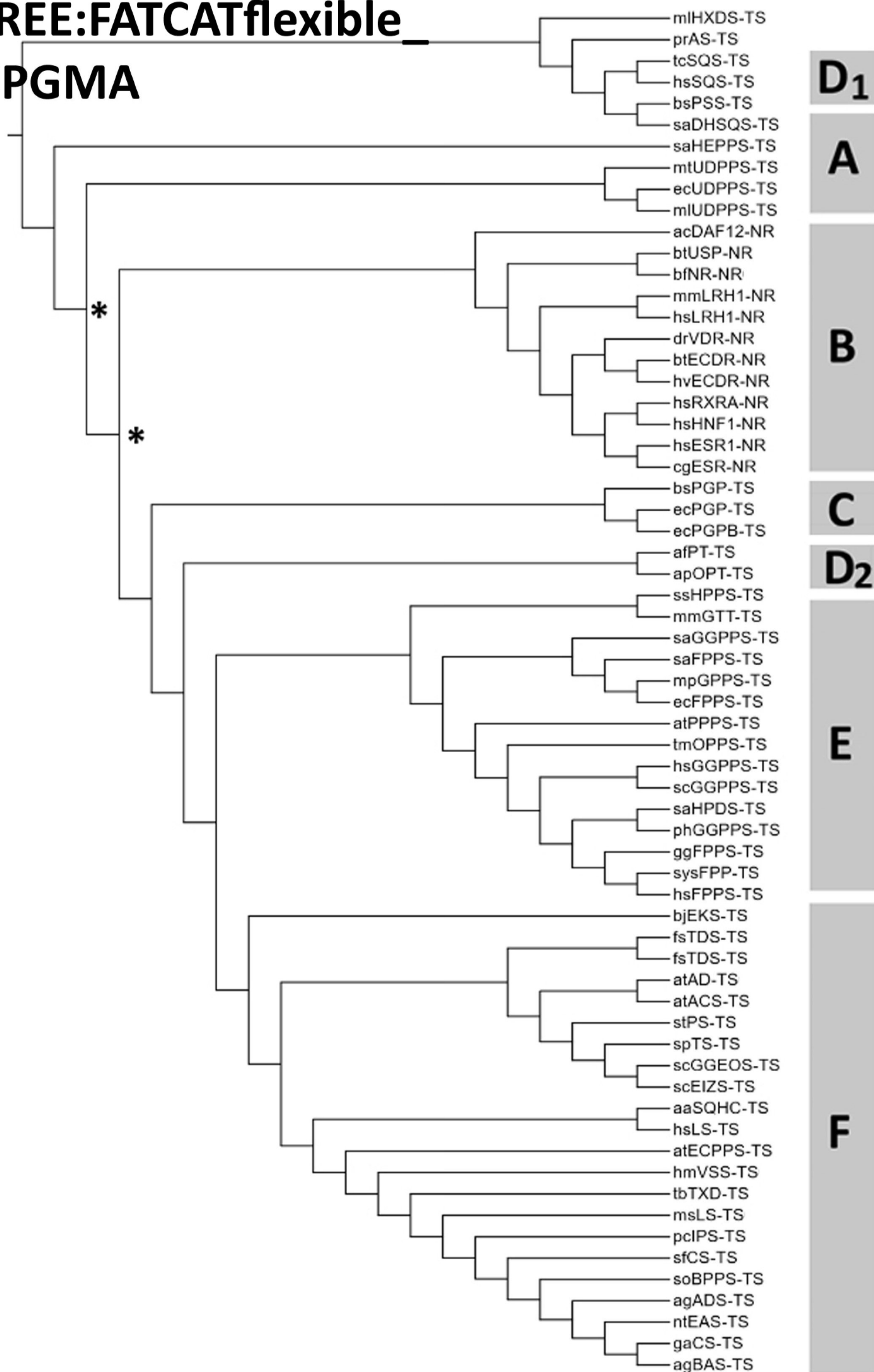

Supplement: Sup fig 2: Tree generated by FATCATflexible + UPGMA. [file supplementary_figure_2.pdf]

# TREE: FATCATflexible\_neighbor-joining

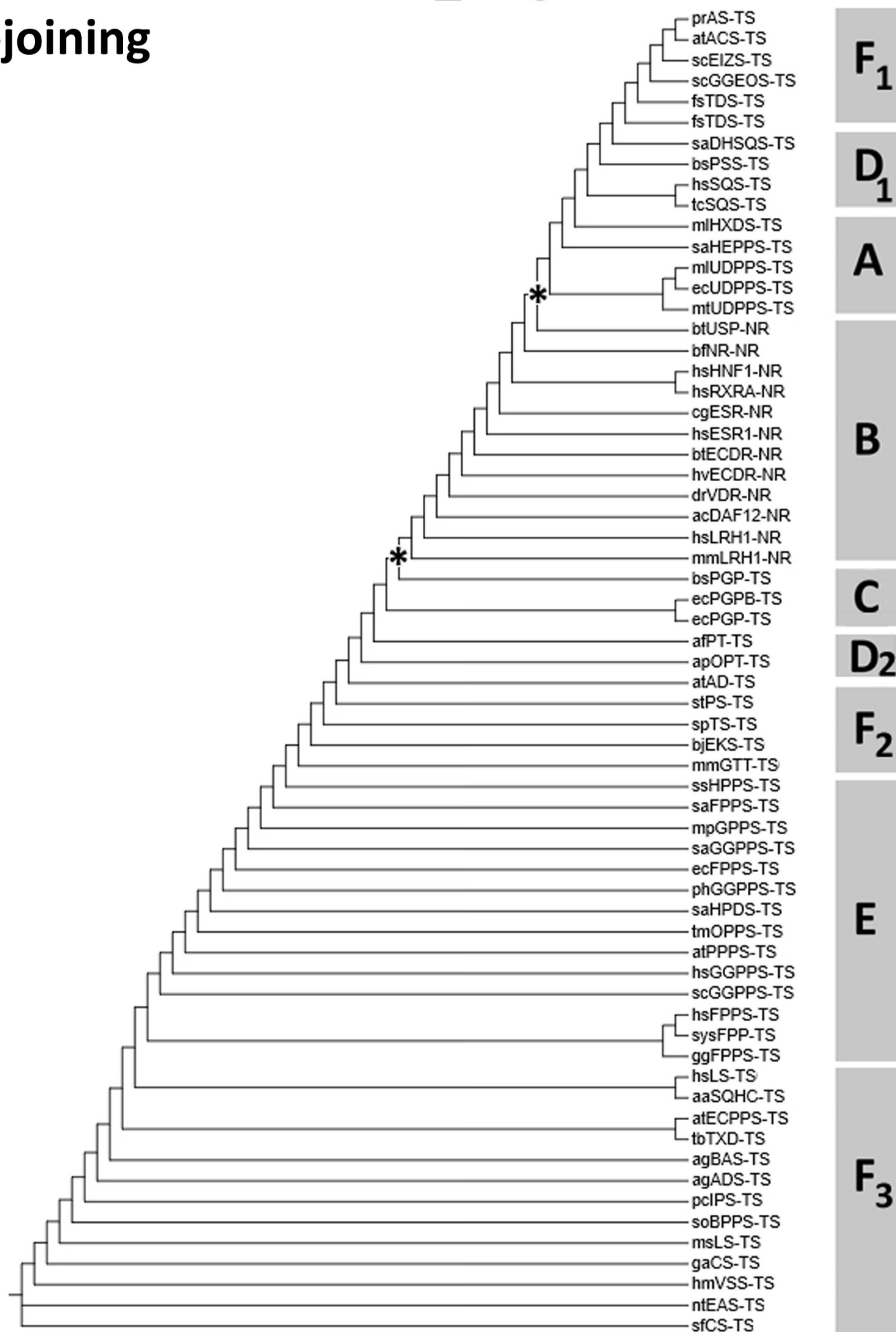

Supplement: Sup fig 3: Tree generated by FATCATflexible + Neighbor-Joining. [file supplementary_figure_3.pdf]

# TREE: FATCATrigid\_UPGMA

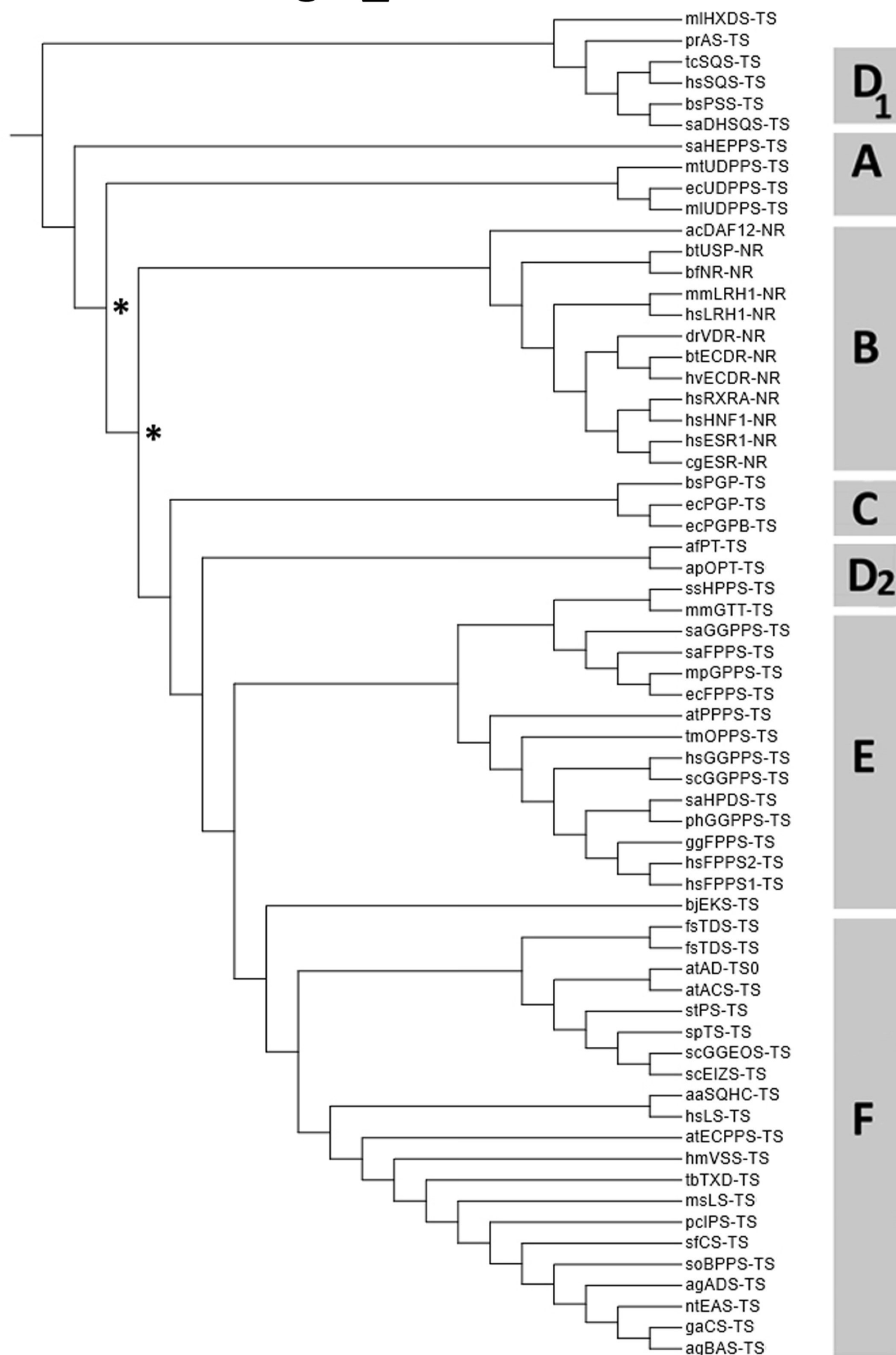

Supplement: Sup fig 4: Tree generated by FATCATrigid + UPGMA. [file supplementary_figure_4.pdf]

# TREE: jCE\_UPGMA

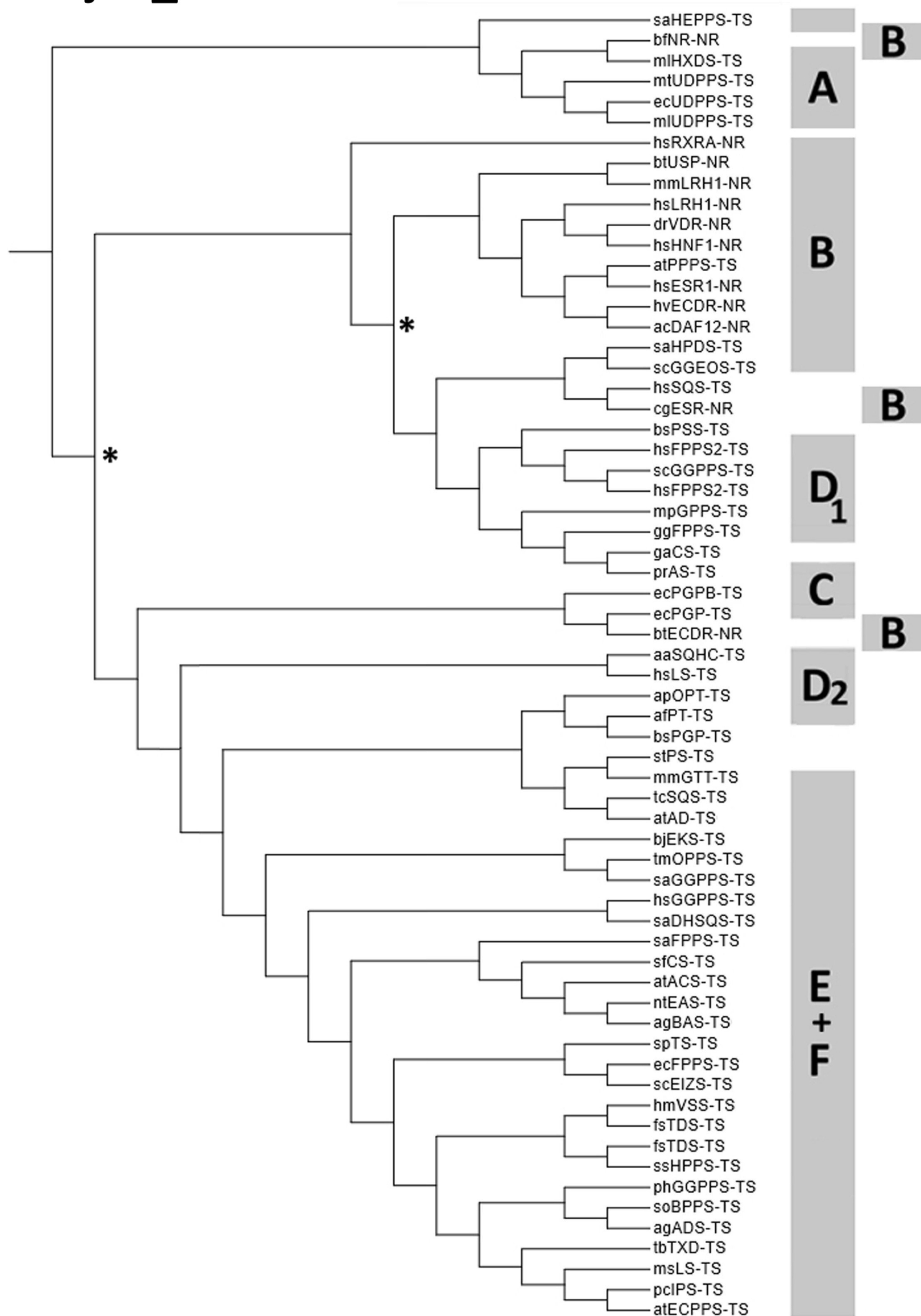

Supplement: Sup fig 5: Tree generated by jCE + UPGMA [file supplementary_figure_5.pdf]

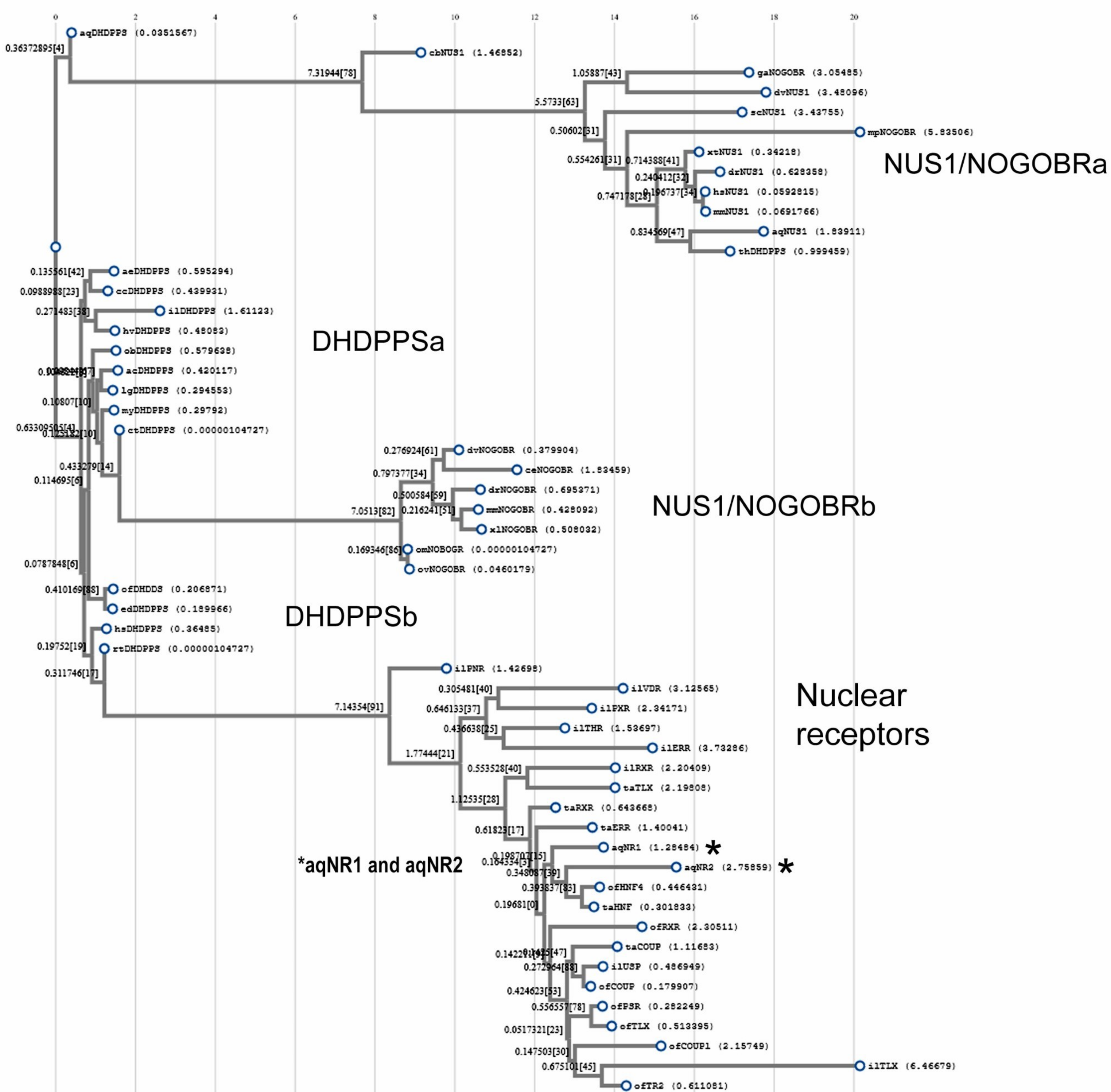

Supplement: Sup Figure 7. Detailed phylogenetic tree of cis-isoprene transferases and nuclear receptors [file supplementary_figure_7.pdf]

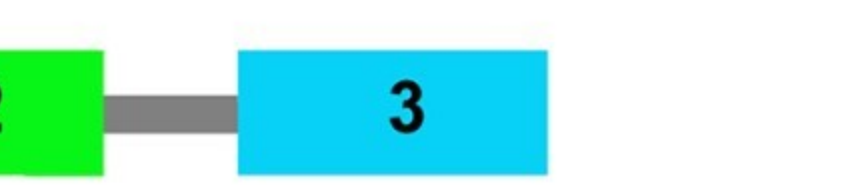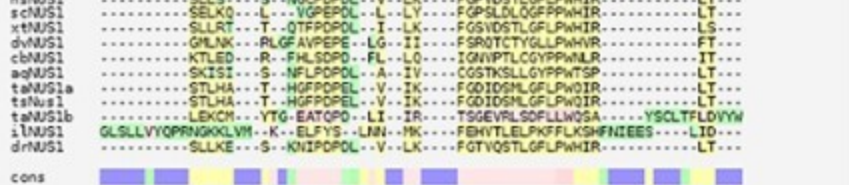

Supplement: Sup Figure 8. Primary sequence alignments of NUS1 proteins and ligand-binding domains (LBDs) using M-coffee. NR LBDs (left), NUS1 sequences (center), and both (right) were aligned using Mcoffee; the three regions of putative conservation (1–3) are highlighted in red, green, and blue, respectively. P [file supplementary_figure_8.pdf]
