# Supplementary material for: Evidence that nuclear receptors are related to terpene synthases
Source: J Mol Endocrinol. 2022 Feb 3;68(3):153–66. doi: 10.1530/JME-21-0156 (PMC8942334; doi:10.1530/JME-21-0156)
Supplement: Sup fig 6: Ligand overlap between Escherichia coli OPPS, PDB 3WJN; and Homo sapiens ESR, PDB 1QKU analyzed by (i) structure overlap, (ii) separate extraction of the overlapped polypeptides, (iii) docking of their respective ligands, and (iv) combining into a single file for visualization [file supplementary_figure_6.pdf]

Supplementary Figure S6

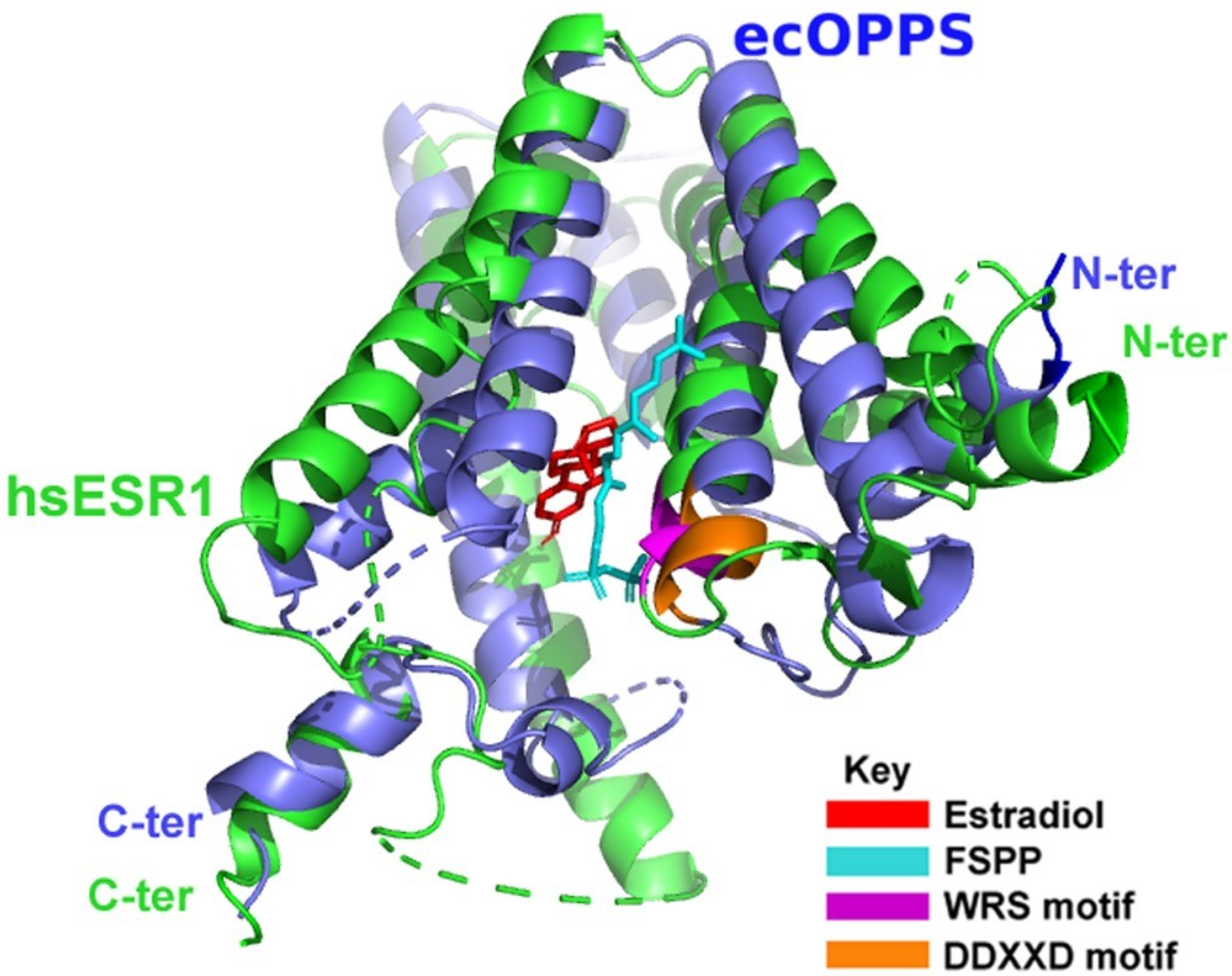

Figure S6. Estradiol and farnesyl-S-thiol pyrophosphate (FSPP) docked into human (hs) ESR1 and *E. coli* (ec) octaprenyl pyrophosphate synthase (OPPS) in a four-step procedure that involved overlap of the two polypeptides, separate extraction of the refolded chains, docking of their respective substrates, and reassembly into a single file for visualization (see Methods for details). The procedure has led to distortion of the structures, but demonstrates overlapping ligand locations within the major pocket.
