# Supplementary material for: Evidence that nuclear receptors are related to terpene synthases
Source: J Mol Endocrinol. 2022 Feb 3;68(3):153–66. doi: 10.1530/JME-21-0156 (PMC8942334; doi:10.1530/JME-21-0156)
Supplement: Sup fig 9: Primary/secondary sequence alignment of key terpene sythase enzymes and nuclear receptors with docking interactions (Sup Table 3). [file supplementary_figure_9.pdf]

Supplementary Figure S9

Consensus Aligment of Terpene Synthases and Nuclear Receptors

: = Conserved catalytic residues
= Contact point
= Contact points in NR2 by inference
ABC = Alpha helix
• = Kink in helix

Numbers in grey give the postions of each residue in the corresponding crystal structure

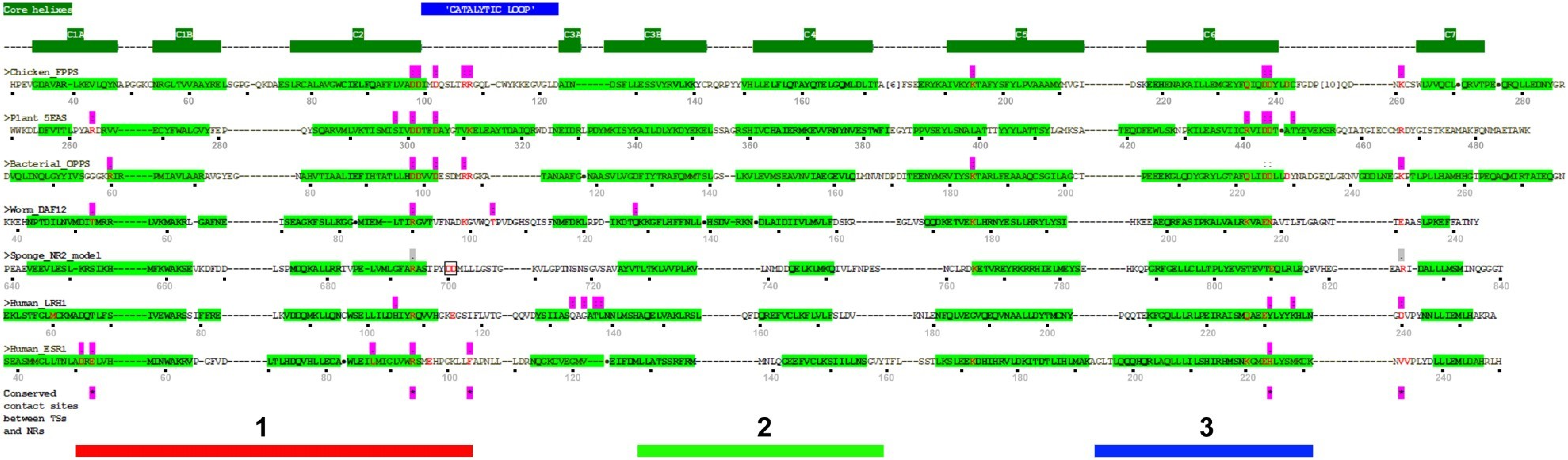

SEQUENCE LIST	PDB
Chicken farnesyl pyrophosphophate synthase (FPPS)	1FPS
Plant 5-epi-aristolochene synthase (5EAS)	5EAT/5EAS
Bacterial octaprenyl pyrophosphate synthase (OPPS)	3WJN
Sponge nuclear receptor NR2	Model (NCBI NP_001266221.1)
Human steroid receptor ESR1	1QKU
Human nuclear receptor LRH1	1YOK
Worm nuclear receptor DAF12	3GYT

ESR1 homology domains with NUS1 — 1 2 3
