# Supplementary material for: Evidence that nuclear receptors are related to terpene synthases
Source: J Mol Endocrinol. 2022 Feb 3;68(3):153–66. doi: 10.1530/JME-21-0156 (PMC8942334; doi:10.1530/JME-21-0156)
Supplement: Supplementary Data S1 [file supplementary_data_s1.pdf]

**Data S1.** Structure comparisons, P values, adjustments, and distribution of values

## *P* values

Each comparison (FATCAT) generates a raw similarity score, a *P* value, and an RMSD. Because the raw score reflects both the extent and the significance of the hsimilarity, this was used for tree building. However, the raw score correlates with the *P* value of the similarity being generated by chance. As shown in the figure below, the *P* values for individual NR versus TS comparisons (FATCATflexible) fell to *P* = 0.004; the likelihood that the similarity between all NR versus TS comparisons might arise by chance could in principle be represented by the mean *P* value multiplied the number of comparisons (i.e., <10e-6).

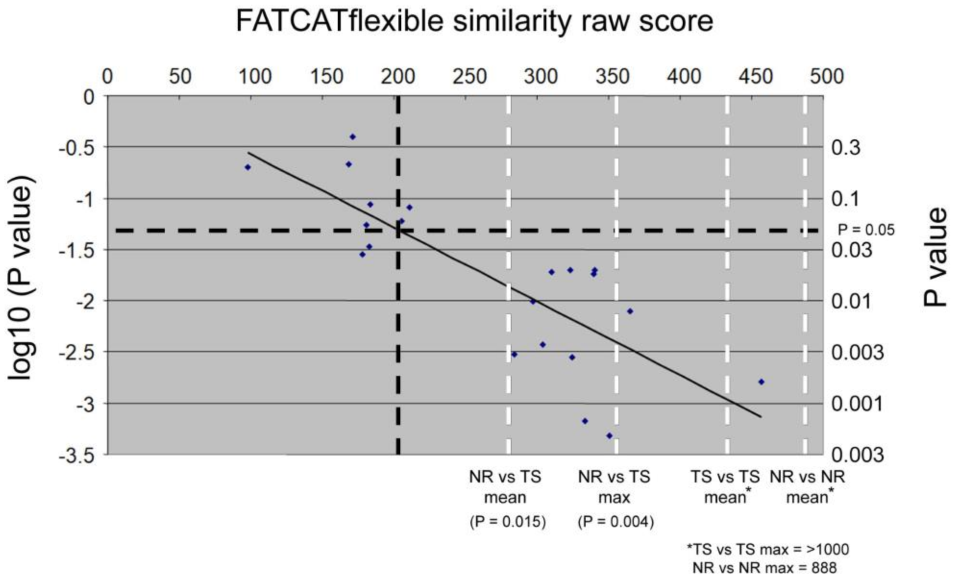


Note: a raw score of 350 (NR vs TS maximum value) equates to a similarity score of 0.35 and a distance score (as in the tables provided) of 0.65.

## Score adjustments

A small number of comparisons (3.1%) generated similarity scores in excess of 1000 (Figure below). Because these are likely to bias the tables of comparisons, and are principally self-

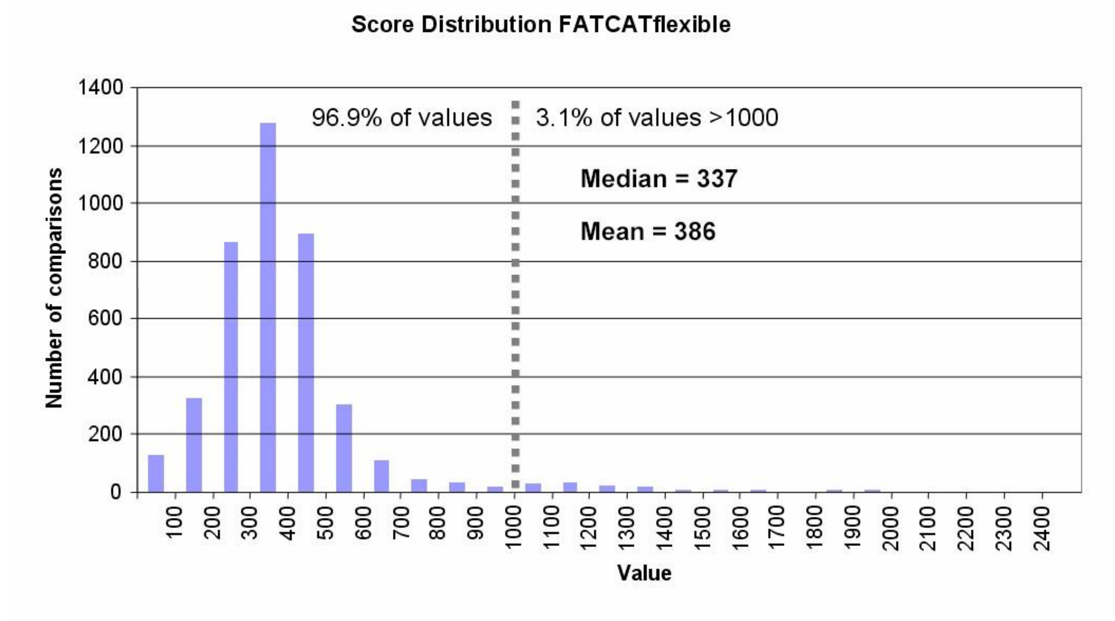

versus self (i.e., identical protein) comparisons (Figure below), scores in excess of 1000 were converted to 1000.00.

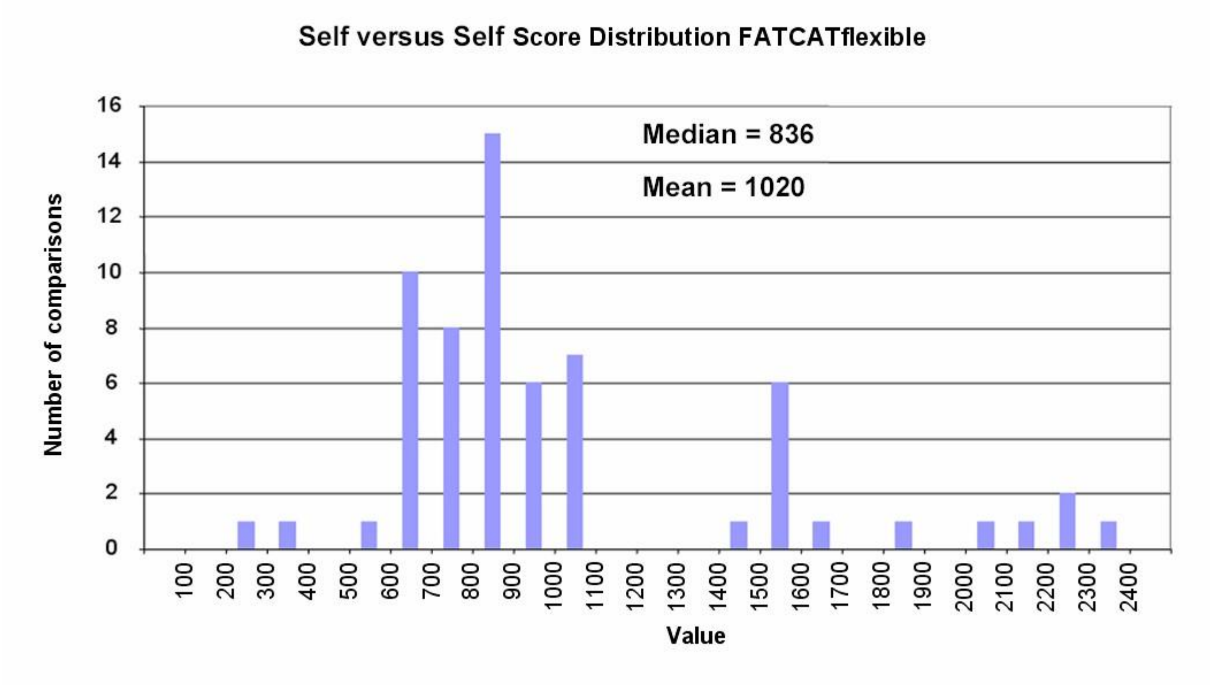


**Distribution of values: minima, maxima, and means**

The table below summarises the range of values for NR/NR, TS/TS, and NR/TS comparisons.

| | MIN | MAX | MEAN |
|---|---|---|---|
| NR VERSUS NR | 331.34 | 888 | 486.1758 |
| NR VERSUS TS | 60.49 | 356.58 | 279.117 |
| TS VERSUS TS | 48.47 | 2328 | 430.5731 |

Some NR vs TS scores therefore exceed TS vs TS scores and approach the mean TS vs TS score.
