# Supplementary material for: Evidence that nuclear receptors are related to terpene synthases
Source: J Mol Endocrinol. 2022 Feb 3;68(3):153–66. doi: 10.1530/JME-21-0156 (PMC8942334; doi:10.1530/JME-21-0156)
Supplement: Supplementary Data S2 [file supplementary_data_s2.pdf]

Supplementary Data S2

Evidence for an Ancestral Internal Duplication within Terpene Synthases

Alignments of vertebrate farnesyl pyrophosphate synthase (FPPS) enzymes from human, chicken, fish (*Danio rerio*), and amphibian (*Xenopus tropicalis*) revealed similarities between the N- and Ctermini of the polypeptides (https://www.ncbi.nlm.nih.gov/tools/cobalt/cobalt.cgi). The N- and Ctermini were therefore separated and aligned using LALIGN (https://embnet.vitalit.ch/software/LALIGN_form.htmland) and displayed using MView (https://www.ebi.ac.uk/Tools /msa/mview/; color = Any) revealing sequence conservation between the two domains, centered on the DDxxD catalytic motif (Figure A; statistical significance computed from LALIGN Z score was P = <0.00001).

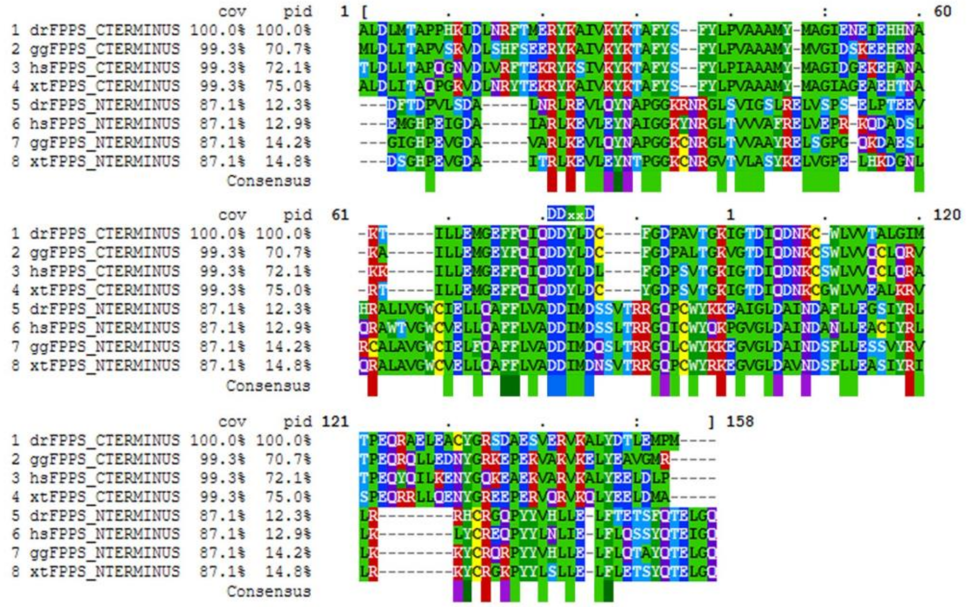

Figure A. Alignment of the N- and C-terminal Domains of Vertebrate FPPS Enzymes

To assess whether this is mirrored in the 3D structure of the protein, the PDB files for chicken FPPS were edited to separate the N- and C-terminal regions, and compared using FATCATflex (http://fatcat.godziklab.org/fatcat/fatcat_pair.html) (Figure B).

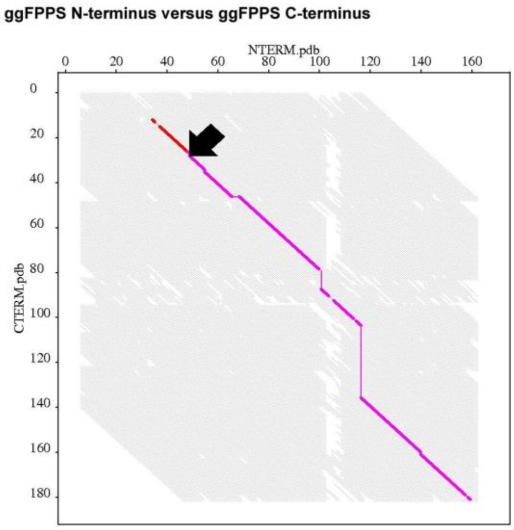

Figure B. Alignment of the tertiary structures of the N- and C-Terminal Domains of Chicken (*Gallus gallus*) FPPS.

The overlapped PDB file of the two domains was downloaded and visualized using PyMol (Figure C), revealing that (in addition to primary structure similarities) the secondary and tertiary structures of the two domains are conserved. Importantly, the dual DDxxD motifs (red and blue in the Figure) in the two domains were found to closely overlap in 3D.

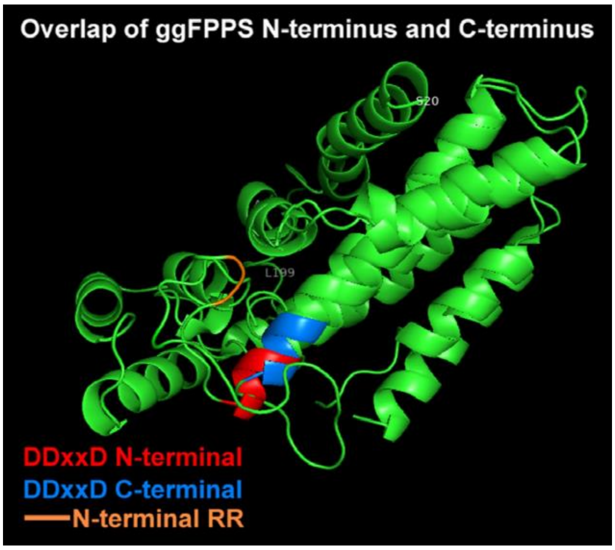

Figure C. 3D Overlap of the N- and C-Terminal Domains of Chicken (*Gallus gallus*) FPPS. The DDxxD motif is highlighted in red/blue.

We conclude that the presence of two DDxxD motifs in vertebrate FPPS enzymes is likely to reflect duplication of a ancestral catalytic domain. Further sequence comparisons revealed that the domain duplication is widely present in terpene synthase (TS) enzymes across species (including Archaea; not presented), as well as in nuclear receptors (NRs; discussed below).

In all TS structures examined, the two domains are arranged in a hip-to-hip conformation such that the two ancestral catalytic sites (DDxxD; representing the extended core helix c2–c3 junction and the duplicated c6–c7 junction) are very close to one another, but do not

occlude ligand access. We surmise that the duplication explains the presence of two ligand-binding motifs in many terpene synthase enzymes: TS enzymes generate product by linking two substrates, and the presence of two ligand-binding pockets and catalytic motifs may reflect dual substrate-binding sites coupled with the need for substrate tunneling into the enzyme followed by product ejection.

In some enzymes the two sites have adopted specialized functions. The C-terminal binding site reported in human FPPS (Jahnke et al. 2010) appears to have evolved into an allosteric site that modulates the activity of the N-terminal binding site (Park et al. 2016): we separately mapped 'orthosteric' (catalytic site) and 'allosteric' contacts in FPPS, and this confirmed that some ligands bound to the allosteric site also directly contact the orthosteric site (Figure D), confirming the 'hipto-hip' configuration.

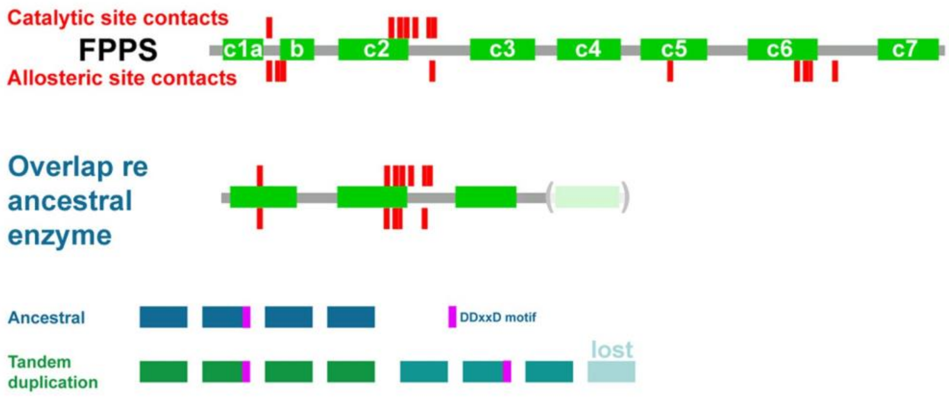

Figure D. Overlap of Contact Points in FPPS for the Catalytic Site and Allosteric Site Supports Ancestral Duplication of a Central Core of 3–4 Helices.

In wider TS enzymes (as in their inferred ancestor) the N-terminal pocket constitutes the major catalytic site, although much diversity is seen (not presented)

Two Domains in Nuclear Receptor Ligand-Binding Domains (LBDs)?

The duplication appears to have taken place early in evolution because it is present in TS enzymes from Archaea, and thus preceded the divergence of NRs. Alignment of NR primary/secondary/tertiary sequences between TS enzymes and NR LBDs (see Main Text) indicates that it may also be present in NR LBDs. However, there was very limited evidence for primary sequence homologies between the inferred N- and C-terminal regions of NR LBDs. However, the secondary and tertiary structures are consistent with the existence of two subdomains in which tandem duplication of core helices c1–c4 (corresponding to H3, H4/5, H6/7, and H8 in NRs) generated a longer peptide containing eight α-helices (c1–c7/8; corresponding to H3–H8 plus H9, H10/11, and H12); the last helix (that would have been c8) seems to have been lost.

In support, mapping of diverse ligand contact sites in NRs reveals a duplicated pattern that corresponds to differential occupancy of two sites according to the ligand and the NR (compare Figure D upper panel with the contact site alignment; Main Text). Selective estrogen receptor modulators (SERMS) compete with E2 for binding to the primary binding site (c2–c3), but because they are larger protrude through into an adjacent pocket constituted by c6–c7 (H11–H12 in NR nomenclature) (e.g., Vajdos et al. 2007; reviewed by Fischer and

Smiesko 2020). In addition, there is evidence that allosteric ligands may occupy a pocket adjacent to the principal ligand-binding cleft. For example, RORγt ligands MRL-871 and T0901317 occupy adjacent binding sites corresponding to c2–c3 and c6–c7 (PDB 4YPQ and 4NB6; this and other examples are reviewed by Meijer et al. 2019) (Figure E).

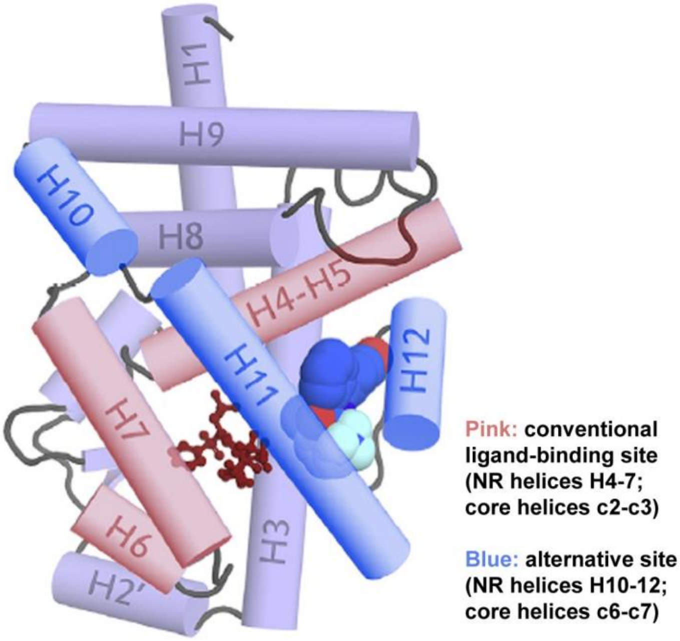

Figure E. Two Binding Sites in Human RORγt (PDB 4YPQ and 4NB6). Note that in TS enzymes the duplicated catalytic sites, centered on the c2–c3 and c6–c7 junctions (Figure D) correspond to the H4/5–H6/7 junction (pink; conventional binding site; ligand in red) and the H10/11–H12 junction (blue; duplicated binding site; space-filling ligand), respectively. Figure adapted from Meijer et al. (2019).

Further investigations will be necessary to address whether the two binding cavities reflect an ancestral duplication within the LBDs of NRs.

Literature cited

Fischer, A. and Smieško, S. Allosteric binding sites on nuclear receptors: focus on drug efficacy and selectivity. Int. J. Mol. Sci. 21, 534.

Jahnke, W. et al. (2010) Allosteric non-bisphosphonate FPPS inhibitors identified by fragment-based discovery. Nat. Chem. Biol. 6, 660–666

Meijer, F.A. et al. (2019) Allosteric small molecule modulators of nuclear receptors. Mol. Cell. Endocrinol. 485, 20–34

Park, J. et al. (2017) Human farnesyl pyrophosphate synthase is allosterically inhibited by its own product. Nat. Commun. 8, 14132

Vajdos, F.F. et al. (2007) The 2.0 A crystal structure of the ERalpha ligand-binding domain complexed with lasofoxifene. Protein Sci. 16, 897–905
