# Supplementary material for: Evidence that nuclear receptors are related to terpene synthases
Source: J Mol Endocrinol. 2022 Feb 3;68(3):153–66. doi: 10.1530/JME-21-0156 (PMC8942334; doi:10.1530/JME-21-0156)
Supplement: Supplementary Data S3 [file supplementary_data_s3.pdf]

Supplementary Data S3

Supplementary Figure: Overlapping docking of ligands and FPP into nuclear receptors

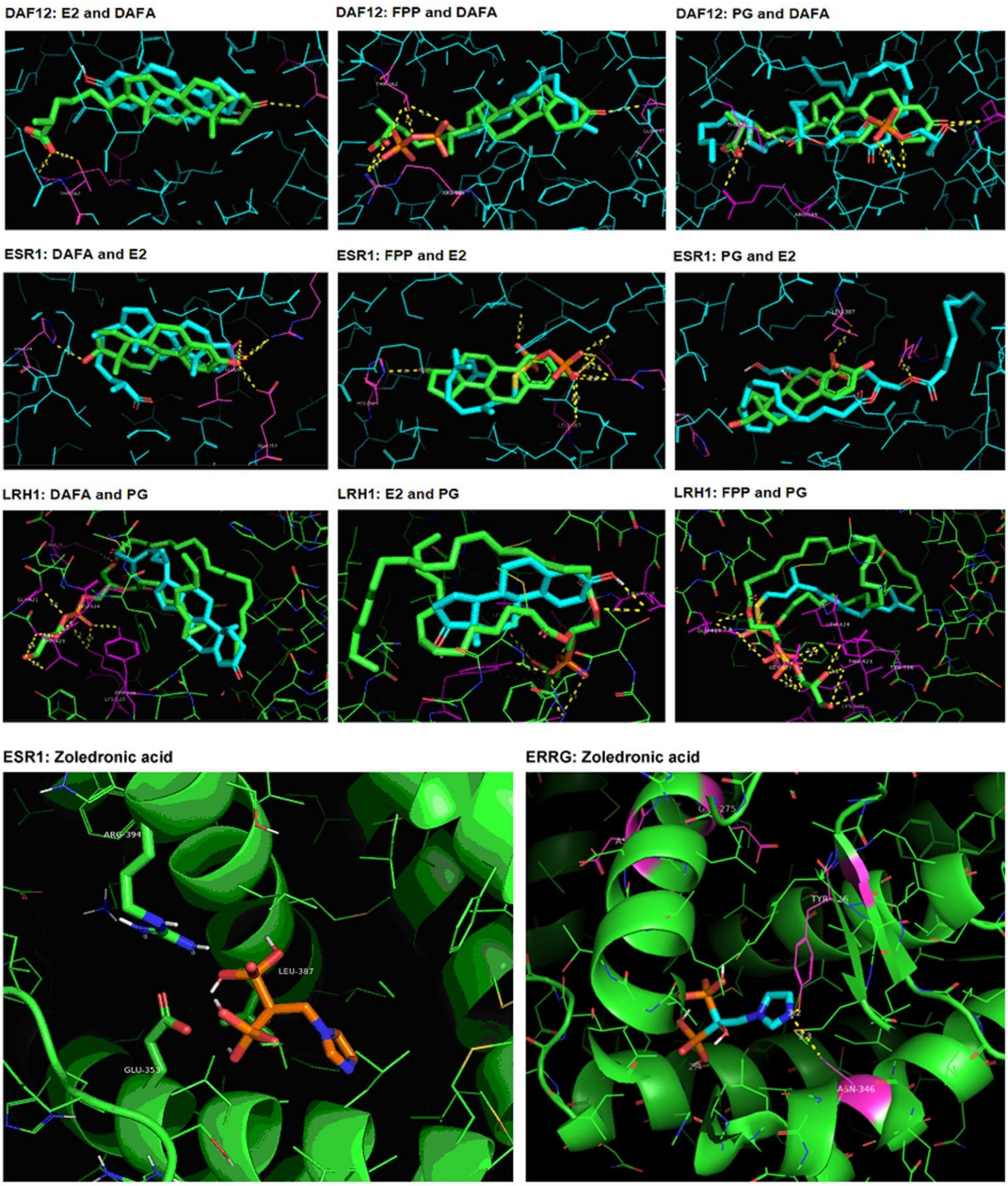

Abbreviations: DAF12, *Caenorhabditis elegans* nuclear receptor (DAF-12); DAFA, dafachronic acid; E2, estradiol; ERRG, human estrogen-related receptor γ, ESR1, human estrogen receptor α,

same site as conventional ligands; the terpene synthase ligand zoledronic acid binds to ESR1 and **DATA S3.** Molecular docking shows that farnesyl pyrophosphate binds to nuclear receptors at the FPP, farnesyl pyrophosphate; LRH1, human liver receptor homolog 1; PG, phosphatidyl glycerol.

ERRG.
